# Interactions Among Expressed MicroRNAs and mRNAs in the Early Stages of Fowl Adenovirus Aerotype 4-Infected Leghorn Male Hepatocellular Cells

**DOI:** 10.3389/fmicb.2020.00831

**Published:** 2020-05-19

**Authors:** Ning Wu, Bo Yang, Bo Wen, Ting Wang, Jiaona Guo, Xuefeng Qi, Jingyu Wang

**Affiliations:** ^1^College of Veterinary Medicine, Northwest A&F University, Xianyang, China; ^2^Shanxi Academy of Advanced Research and Innovation, Taiyuan, China

**Keywords:** fowl adenovirus serotype 4, entry, leghorn male hepatocellular cells, RNA-seq, mRNA–miRNA integrate analysis

## Abstract

Hydropericardium-hepatitis syndrome (HHS) is caused by some strains of fowl adenovirus serotype 4 (FAdV-4). However, the mechanism of FAdV-4 entry is not well understood. Therefore, to investigate the changes in host cellular response at the early stage of FAdV-4 infection, a conjoint analysis of miRNA-seq and mRNA-seq was utilized with leghorn male hepatocellular (LMH) cells at 30, 60, and 120 min after FAdV-4 infection. In total, we identified 785 differentially expressed (DE) miRNAs and 725 DE mRNAs in FAdV-4-infected LMH cells. Most miRNAs and mRNAs, including gga-miR-148a-3p, gga-miR-148a-5p, gga-miR-15c-3p, CRK, SOCS3, and EGR1, have not previously been reported to be associated with FAdV-4 infection. The conjoint analysis of the obtained data identified 856 miRNA–mRNA pairs at three time points. The interaction network analysis showed that gga-miR-128-2-5p, gga-miR-7475-5p, novel_miR205, and TCF7L1 were located in the core of the network. Furthermore, the relationship between gga-miR-128-2-5p and its target OBSL1 was confirmed using a dual-luciferase reporter system and a real-time quantitative polymerase chain reaction assay. *In vitro* experiments revealed that both gga-miR-128-2-5p overexpression and OBSL1 loss of function inhibited FAdV-4 entry. These results suggested that gga-miR-128-2-5p plays an important role in FAdV-4 entry by targeting OBSL1. To the best of our knowledge, the present study is the first to analyze host miRNA and mRNA expression at the early stage of FAdV-4 infection; furthermore, the results of this study help to elucidate the molecular mechanisms of FAdV-4 entry.

## Introduction

Hydropericardium-hepatitis syndrome (HHS) is caused by fowl adenovirus serotype 4 (FAdV-4) (Asthana et al., 2013). HHS, first observed in Pakistan in 1987, causes significant losses to the poultry industry (Afzal et al., 1991; Ojkic et al., 2008). Recently, HHS has been reported in Chile, Japan, Korea, India, Iraq, Russia, and Mexico (Mase et al., 2010; Liu et al., 2016). From 2014, HHShas caused severe losses to the broiler industry in most of North and South China (Liu et al., 2016; Zhao et al., 2019). The clinical manifestations of HHS are decreased feed intake, lethargy, ruffling of neck feathers, and depression (Liu et al., 2016). The predominant gross lesion constitutes hydropericardium, characterized by the accumulation of a clear or jelly-like fluid in the pericardium (Zhao et al., 2019). The HHS agent is highly pathogenic and shows a high affinity toward hepatic cells (Chen et al., 2019). The hepatocellular carcinoma epithelial cell line has been used as a homologous cell line for the study of host gene expression for years, which was established in 1981 (Kolluri et al., 1999; Yamauchi et al., 2005). In recent years, studies have shown that an important aspect of the interaction between FAdV-4 and its host is the change of cell gene transcription (Zhang et al., 2018; Ren et al., 2019). Nevertheless, the role of cellular microRNAs (miRNAs) during FAdV-4 infection has not been determined.

microRNAs have emerged as important regulators of gene expression at the posttranscriptional level (Bartel, 2004; Bushati and Cohen, 2007; Wang et al., 2007). Previous studies have shown that some host cellular miRNAs interact with viruses during infection (Otsuka et al., 2007; Pedersen et al., 2007). Many cellular miRNAs affect viral replication indirectly by targeting the host factors related to replication (Shrivastava et al., 2015). For example, miR-485 increases viral replication by degrading the host’s retinoic acid inducible-gene I gene during avian influenza virus (AIV) or Newcastle disease virus (NDV) infections (Ingle et al., 2015). The host miR-17/92 cluster directly suppressed the expression of some cellular factors, leading to the inhibition of HIV-1 replication (Triboulet et al., 2007). The infection with the adenovirus is also considered to be regulated by certain miRNAs. Previous studies have shown that the upregulation of miR-155 during the early phase of adenovirus infection plays a role in the host antiviral response (Zhao et al., 2015). Previous studies have also shown that some miRNAs degrade mRNAs that interact directly with viral entry. The miR-27-mediated suppression of target mRNA expression led to the inhibition of adenovirus infection at the step of viral entry (Machitani et al., 2017). Furthermore, the adenovirus receptor (CAR) was one of the miRNA targets involved in viral entry (Lam et al., 2015). However, the role of cellular miRNAs in FAdV-4 entry is not well understood.

In our study, we concentrated on FAdV-4-infected leghorn male hepatocellular (LMH) cells at the gene level, particularly during the early stage of infection. According to using the miRNA-seq and mRNA-seq profiles, we analyzed the genes and the pathways related to virus entry. We demonstrate here that gga-miR-128-2-5p suppresses OBSL1 expression *via* posttranscriptional gene silencing, leading to the inhibition of FAdV-4 entry into cells. Taken together, this is the first study of early host interactions in LMH cells, which helps to elucidate the mechanism of FAdV-4 transmission and identifies potential targets for future studies.

## Materials and Methods

### Cells, Viruses, and Antibodies

Leghorn male hepatocellular cells were kindly provided by Prof. Yunfeng Wang (Harbin Veterinary Research Institute, Heilongjiang, China) and cultured in Dulbecco’s modified Eagle’s medium (DMEM; Sigma, MO, United States) supplemented with 10% fetal bovine serum (FBS; Sigma, MO, United States). The FAdV-4 isolate SX17 (GenBank: MF592716.1) used in our study was isolated from a liver sample of a broiler chicken during a recent HHS outbreak in Shaanxi Province in western China. The rabbit polyclonal anti-FAdV-4-fiber antibody was generated by our laboratory. The horseradish peroxidase-conjugated secondary antibodies and the FITC-conjugated anti-rabbit IgG were purchased from Transgen Biotechnology (Beijing, China).

### Kinetics of Viral Internalization

The LMH cells were cultured in 12-well plates (3 × 10^5^ cells/well). To measure the effectiveness of proteinase K treatment, 12-well plates were divided into control group, protease K treatment group, and phosphate-buffered saline (PBS) treatment group of four wells each. The cells were infected with FAdV-4-isolated strain at a multiplicity of infection (MOI) of 10 and shifted to 4°C for 1 h, then the cells were washed with PBS, and then four wells were collected as a control group. The protease K treatment group was treated with proteinase K (2 mg/ml) (Solarbio, China) for 45 min at 4°C to remove the adsorbed but not internalized virus. The PBS treatment group was processed under the same conditions, except that proteinase K was replaced with PBS. Proteinase K was then inactivated with 2 mM phenylmethylsulfonyl fluoride in PBS with 3% bovine serum albumin (BSA), and the cells were washed with PBS–0.4% BSA. Finally, the cells were collected for DNA isolation (Yang et al., 2018). The genomic level of FAdV-4 in the control group was considered as 100%. The genomic levels of FAdV-4 were quantified by absolute quantitative real-time PCR assay and normalized to GAPDH as described previously (Gunes et al., 2012). The FAdV-4 Hexon gene (NCBI GenBank accession number KU569296.1) was used as an indicator for the presence of viral DNA. The primer sets targeting Hexon included forward primer 5′-ACAGGTCCTCAGCTACAAGA-3′ and reverse primer 5′-TGACCCTAACGGTGTCGA-3′.

To determine the rate of virus internalization, the LMH cells were pretreated with FAdV-4 for 1 h at 4°C, which was then shifted to 37°C. At different time points, the cells were washed with PBS and treated with proteinase K for 45 min at 4°C. Proteinase K was then inactivated, and the cells were washed with PBS–0.2% BSA by low-speed centrifugation. Then, the cell pellet was resuspended in DMEM and different serial dilutions of the cell suspensions were plated onto LMH cell monolayers. The LMH cells were grown in 96-well plates containing DMEM with 2% FBS. The cells were then incubated at 37°C and 5% CO_2_ for 4–6 days, and the number of wells with or without CPE was counted. TCID50 was calculated with the Reed–Muench method and used to calculate the infectivity of the viral stocks: infectivity (plaque-forming units/ml) = 0.69 × TCID50. A parallel set of cultures was processed under the same conditions, except that proteinase K was replaced with PBS. Each test was performed in triplicate.

### Confocal Laser Scanning Microscopy

The LMH cells were grown on coverslips to a confluency of 80% and pretreated with FAdV-4 at 4°C for 1 h, which was then shifted to 37°C. The LMH cells were then washed four times with PBS and fixed in 4% paraformaldehyde at 0, 30, 60, and 120 min after the FAdV-4 infection, respectively. The LMH cells were then washed four times again with PBS and treated with 0.1% Triton X-100 for 10 min. The LMH cells were then incubated with 1% BSA and appropriate primary antibodies for 1 h at 37°C. Then, the LMH cells were washed and further incubated with FITC- or TRITC-conjugated secondary antibodies. The actin filaments were stained with TRITC-phalloidin (2 mg/ml) for 30 min at 25°C. Finally, the LMH cells were treated with 1 mg/ml of 4′,6-diamidino-2-phenylindole solution for 15 min and analyzed by confocal laser scanning microscopy (CLSM Leica SP8, Germany).

### Transmission Electron Microscopy

Ultra-thin sections (70 nm) of cells were prepared and examined under a Hitachi HT-7700 transmission electron microscope (Hitachi High Technologies Co., Japan) as described previously (Ishii et al., 2010; Huang et al., 2015). Briefly, monolayers of LMH cells were infected with FAdV-4 at a MOI of 10. After adsorption at 4°C for 1 h, the samples were shifted to 37°C for 0, 30, 60, and 120 min, respectively. The LMH cells were then collected by centrifugation at 800 *g* for 5 min for subsequent sample preparation.

### Virus Inoculation and RNA Isolation

The LMH cells were cultured for 24 h to obtain 80% confluence. Then, the LMH cells were washed twice with PBS and infected with the FAdV-4 isolated strain at a MOI of 10. The FAdV-4-infected cells were harvested at 30, 60, and 120 min. The non-infected LMH cells were used as a mock control. There were four groups: mock, 30-min-infected, 60-min-infected, and 120-min-infected cells. Each group was processed with three independent replicates. Total RNA was extracted from the cells using TRIzol (Ambion, Life Technologies, Carlsbad, CA, United States) according to the manual instructions (Fu et al., 2018). Subsequently, total RNA was qualified and quantified using a Nano Drop and Agilent 2100 Bioanalyzer (Agilent Technologies, Santa Clara, CA, United States).

### miRNA Sequencing and Data Analysis

Twelve RNA libraries were generated with total RNA from the samples. The total RNA was purified by polyacrylamide gel electrophoresis (PAGE), and small RNA regions corresponding to the 18- to 30-nt bands were recovered. Then, small RNAs were ligated to a 5′-adaptor and a 3′-adaptor. The PCR products were generated after reverse-transcription (RT)-PCR and isolated using PAGE. Then, the BGISEQ-500 platform (BGI-Shenzhen, China) was used to sequence the purified cDNAs (Bao et al., 2018). Differentially expressed (DE) miRNAs between the different samples were measured by | log2ratio| ≥ 1 and *q*-value | FDR| ≤ 0.05. RNAhybrid, miRanda, or TargetScan was used for the prediction of the targets of the differentially expressed miRNAs. Finally, we used GO functional enrichment analysis and KEGG pathway enrichment analysis to identify the functions of these miRNAs or their targets (Xie et al., 2011).

### mRNA Sequencing and Data Analysis

Twelve total RNA samples were obtained to generate the cDNA libraries for each sample according to the manufacturer’s instructions (Fu et al., 2018). Next, the samples were used to enrich poly(A) mRNA, which was fragmented into short pieces by oligo (dT) magnetic beads (Invitrogen). These short mRNA and random hexamers were used to generate the first cDNA, and then the second cDNA was synthesized. The cDNA fragments were ligated to sequencing adapters and amplified by PCR to obtain the final paired-end library (Guan et al., 2018). The sequence used BGIseq-500 (BGI-Shenzhen, China). Differentially expressed genes (DEGs) between the different samples were measured by | log2ratio| ≥ 1 and *q*-value | FDR| ≤ 0.05. The biological functions of the selected genes were analyzed using GO and KEGG pathway enrichment.

### miRNA–mRNA Integrative Genomic Analysis

The interactions among the DEs and DEGs were predicted using their Pearson data. Based on the different expression levels of miRNA–mRNA in the same sample, we used the R package to calculate their Pearson data. It is generally considered that the absolute value of the Pearson data is above 0.6, which has interdependency. The biological functions of the negative miRNA–mRNA correlations were analyzed using GO and KEGG pathway enrichment. Furthermore, based on the integrated analysis of negatively correlated DE miRNA–mRNA pairs (Pearson ≥ 0.6), we used Cytoscape software to construct an interaction network of the screened pairs (Shannon et al., 2003). Finally, the relationship between miRNA and mRNA was verified.

### Real-Time qPCR

We employed relative quantification methods to validate the expression of the target miRNAs and mRNAs. The primers for selected miRNAs were designed by RiboBio Inc (GuangZhou. China), and the sequences are covered by a patent. The primers of mRNAs are listed in Table 1. Real-time qPCR was conducted using the miRcute miRNA qPCR Detection Kit (Takara, Otsu, Japan) for miRNAs or 2 × RealStar greenpower mixture (Tiangen, Beijing, China) for mRNAs according to the manufacturer’s instructions. The expression of the genes was calculated using 2^–ΔΔ*CT*^ as previously described (Shannon et al., 2003). For each sample, three-well replicates were used, and the data were expressed as mean ± standard error (*n* = 3).

**TABLE 1:** Primers used in this study.

Primers	Sequences (5′–3′)
TCF7L1	F: CCCACATGCACCCATTGAC
	R: ATCTGACCCACGGCTCCTG
CREBZF	F: AGCTCCTGAGCCGCTTGGC
	R: GCAGAACTCCACCGACACCTGA
LFNG	F: CTGAAGAAGCAAGCACGAAA
	R: CCGTGGCAAACCAGAAAT
FRMD6	F: TGCCCTTCCACAACAACC
	R: TGACGCTGAGCCCAAAGA
SERTAD2	F: GACTTGACCCTGGATGACA
	R: TAAGACCCGACAAGCACC
OBSL1	F: GAGCACGGAGCAGAGCAAC
	R: GGCACAGACTCGGAAGCAGTAG
SOX4	F: GCGGAAGATCATGGAGCAGTCG
	R: CCTGGGCCGGTACTTGTAGTCG
NFATC1	F: CTATCTTCCTGCTAATGTTCC
	R: GCTGGTTGTAGTACGGTTT
AQP11	F: GCACTTTATCTGGAGGTTGG
	R: CTGTAGCTGGGCTTAATGTTT

### Gene Clone and Vector Construction

The gga-miR-128-2-5p mimics, inhibitor, and siRNA target against the OBSL1 were synthesized by Ribobio (Guangzhou, China). The 3′UTR of OBSL1 and its mutation were cloned into psiCHECK2 and named psiCHECK2-OBSL1 (WT-OBSL1) and psiCHECK2-OBSL1-mut (Mut-OBSL1).

### Transfection With siRNA, miRNA Mimics, and Inhibitors

Cells were transfected with the siRNAs, miRNA mimics, or inhibitors by the use of Lipofectamine 2000 transfection reagent (Invitrogen) according to the manufacturer’s instructions. Briefly, the LMH cells grown to 80% confluence in six-well cell culture plates were transfected with 4 mg/well of siRNAs, miRNA mimics, or inhibitors using a transfection reagent. Then, the cells were cultured in 5% CO_2_ and at 37°C for 48 h. The reaction mixture was discarded and the cells were then infected with FAdV-4 at a MOI of 10. At 120 min post-infection, the cells were collected for real-time PCR assay or confocal microscopy analysis.

### Luciferase Assay

The luciferase reporter assay was performed as previously described with slight modifications (Xiao et al., 2015). Briefly, HEK-293T cells were co-transfected with 500 ng of WT-OBSL1/Mut-OBSL1 plasmid and 500 ng gga-miR-128-2-5p of mimics, inhibitor, or miR-NC using Lipofectamine 2000 transfection reagent (Invitrogen) as described above. The cells were lysed 24 h after transfection, and the luciferase activity in the total cell lysates was measured using a dual-luciferase reporter assay system (Beyotime, Shanghai, China) according to the manufacturer’s instructions.

### Statistical Analysis

The data are expressed as mean ± standard deviation (SD). Comparisons were performed using one-way analysis of variance for viral load and Student’s *t*-tests for mRNA levels using GraphPad Prism 6.0 software (Graph Pad Software Inc., San Diego, CA, United States). A *P* value < 0.05 was considered to indicate statistical significance.

## Results

### Kinetics of FAdV-4 Internalization Into LMH Cells

**FIGURE 1:**
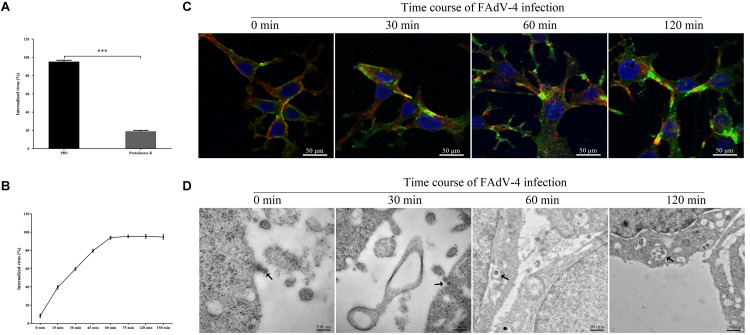
Kinetics of FAdV-4 internalization into leghorn male hepatocellular (LMH) cells. **(A)** The effectiveness of proteinase K treatment; results are shown as a percentage of FAdV-4 copy number of the proteinase K-treated group and the phosphate-buffered saline (PBS)-treated group compared with controls and are presented as the mean ± SD of three independent experiments; ***P < 0.001. **(B)** The rate of FAdV-4 internalization into LMH cells; results are shown as a percentage of internalized viruses compared with controls in which PBS was substituted for proteinase K. **(C)** Confocal laser scanning microscopy (CLSM) analysis of actin filaments (red), viral particles (green), and cell nuclei (blue) in FAdV-4-infected LMH cells at the indicated time points post-infection. **(D)** Microscopic analysis of FAdV-4 entry into LMH cells at specified time points after infection. Transmission electron microscopy image showing the localization of virions. The virions are indicated by black arrows.

Figure 1A shows that proteinase K significantly affected the number of attached virions at the cell membrane, indicating that proteinase K removes the virus attached to the cell membrane. As shown in Figure 1B, at 60 min, most viruses were resistant to proteinase K treatment, indicating the successful internalization of the infectious virions. After 120 min of incubation, almost all the viruses were resistant to proteinase K treatment. To investigate FAdV-4 entry in LMH cells, initial experiments were performed to analyze the uptake of FAdV-4 into LMH cells at the indicated time points using confocal laser scanning microscopy. As shown in Figure 1C, FAdV-4 was adsorbed onto the cell surface but not inside the cells at 0 min. FAdV-4 appeared in the lower layer of the cell membrane at 30 min. Most viral particles were internalized and distributed within the boundaries of the cells at 60 min. Furthermore, FAdV-4 was successfully internalized into the LMH cells at 120 min as shown by the gradual intensification of the green fluorescent signal specific to FAdV-4, which peaked at 120 min. To more accurately access the distribution of FAdV-4, we used transmission electron microscopy (Figure 1D). FAdV-4 virions are pleomorphic in shape and vary in size from 70 to 100 nm (Asthana et al., 2013). In the present work, the enveloped FAdV-4 particles of about 100 nm were initially observed to be attached to the LMH surface at 0 min post-infection. FAdV-4 triggered plasma membrane invagination, forming a cave-like structure wrapped around the bound viruses at 30 min post-infection. The newly formed vesicles can be observed at 60 min post-infection. The vesicle then traveled to the cytoplasm after 120 min. The analysis by CLSM and TEM, along with viral internalization data, suggested that the cycle of FAdV-4 entry is 30–120 min in the cultured LMH cells. Sixty minutes after FAdV-4 infection was considered as the optimal time point to measure viral entry. Therefore, we chose to study FAdV-4–host interactions at 30, 60, and 120 min.

### Construction and Sequencing Data Analysis of miRNA Libraries

**FIGURE 2:**
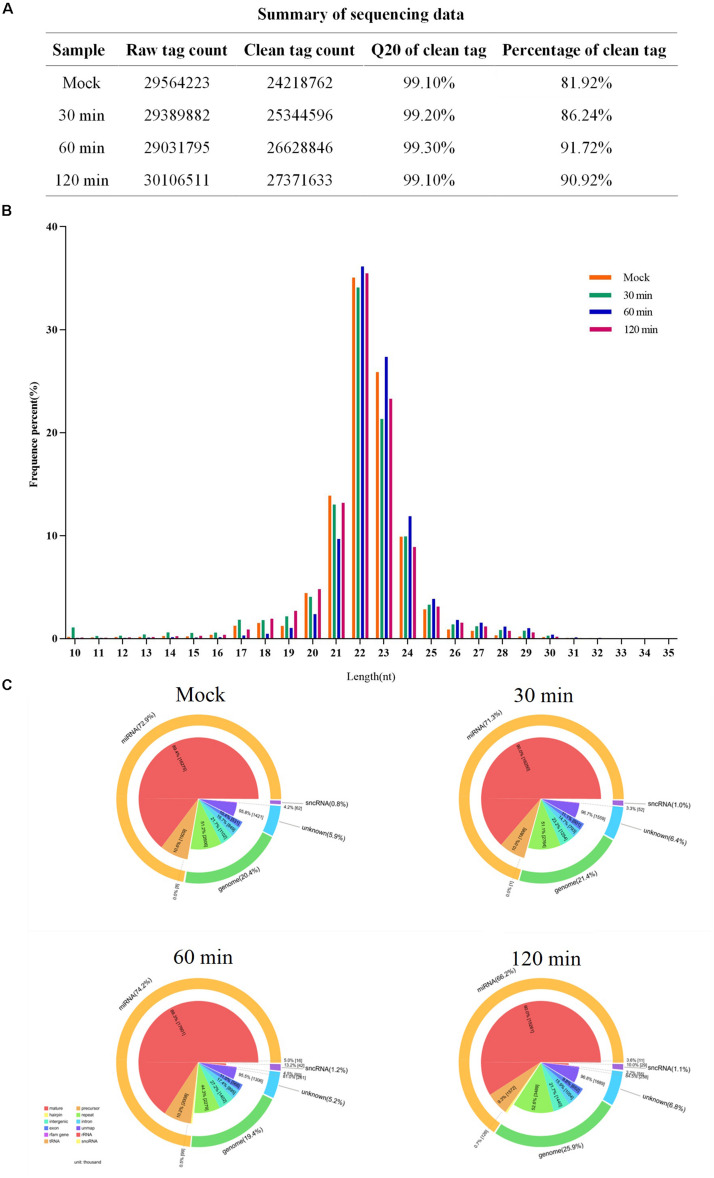
Summary of sequencing data for each sample. **(A)** The sequencing data for each sample. **(B)** Size distribution of sequenced miRNA-seq reads. The majority of miRNAs in FAdV-4-infected and mock LMH cells measure 21–23 nt in length. **(C)** Pie charts of miRNA-seq showing the percentage of miRNA components in FAdV-4-infected and mock LMH cells.

Before data analysis can be carried out, low-quality tags need to be removed. Figure 2A summarizes the sequencing data for each sample. Most clean reads were 21–24 nt in length, and the 22-nt sRNAs were the most abundant (Figure 2B). The proportions of miRNAs were 78.6, 68.4, 74.2, and 66.2% in the mock, 30-min-infected, 60-min-infected, and 120 min-infected groups, respectively (Figure 2C). Our results show that the miRNAs have been enriched successively from the libraries.

### Differentially Expressed miRNAs and Analysis

**FIGURE 3:**
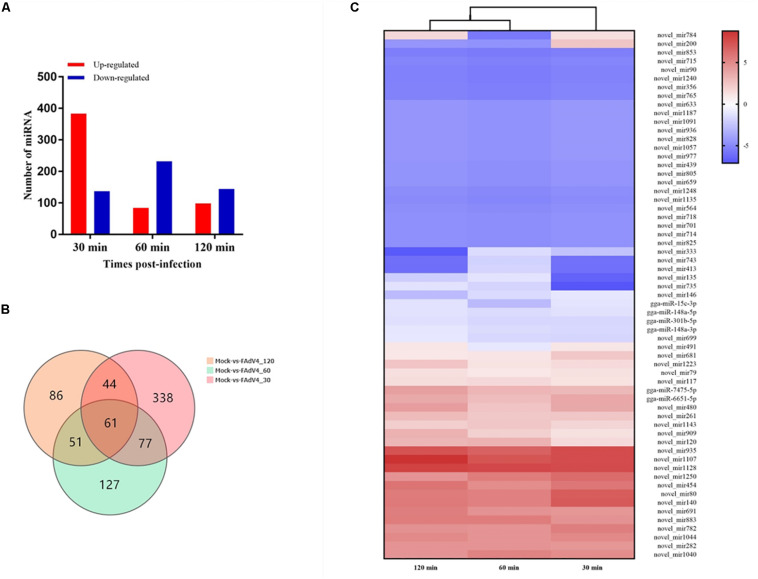
Differential expression profiles of miRNAs in LMH cells. **(A)** Number of differentially expressed miRNAs at three time points after FAdV-4 infection. Red and blue represent the number of upregulated and downregulated miRNAs, respectively. **(B)** Venn diagram showing the unique and co-differentially expressed miRNAs in response to FAdV-4 infection at three time points. **(C)** Hierarchical clustering analysis of 61 differentially expressed miRNAs at all three time points. The color in the heat map represents the gene expression changes. Red indicates the upregulation of gene expression, blue indicates the downregulation of expression, the darker color indicates a notable degree of differential gene expression, and white indicates no activity.

Differentially expressed miRNA screening aims to identify differentially expressed miRNAs between samples and perform further analysis. We used the DEGseq method to perform this analysis. The identification of differentially expressed miRNAs between the mock and the infected groups was based on a *q*-value threshold <0.01 and | log_2_ (fold change)| > 1. In total, 784 miRNAs were identified in the LMH cells after FAdV-4 infection at the three time points (Figure 3A). To make it easier to see the changes in gene expression, three differentially expressed clusters containing these miRNAs were identified (Supplementary Figure S1). Among the miRNAs, 30 min of infection was found to upregulate 383 miRNAs and downregulate 137 miRNAs (Supplementary Table S1), 60 min of infection was found to upregulate 84 miRNAs and downregulate 232 miRNAs (Supplementary Table S2), while 120 min of infection increased the expression of 98 miRNAs and decreased the expression of 144 miRNAs (Supplementary Table S3). Sixty-one miRNAs were differentially expressed at all three time points (Figure 3B). By analyzing their expression, three differentially expressed clusters containing 61 miRNAs were identified (Figure 3C). Among these miRNAs, gga-miR-15c-3p, gga-miR-148a-5p, and gga-miR-148a-3p were downregulated at all three time points. In addition, gga-miR-1662, gga-miR-1454, and gga-miR-3538 were differentially expressed at 120 min (Supplementary Table S3). These results suggested that different miRNA regulation characteristics exist among FAdV-4 infections at different time points and these miRNAs may be involved in virus infection.

### Functional Analyses of Target mRNAs of Differentially Expressed miRNAs During FAdV-4 Infection

**FIGURE 4:**
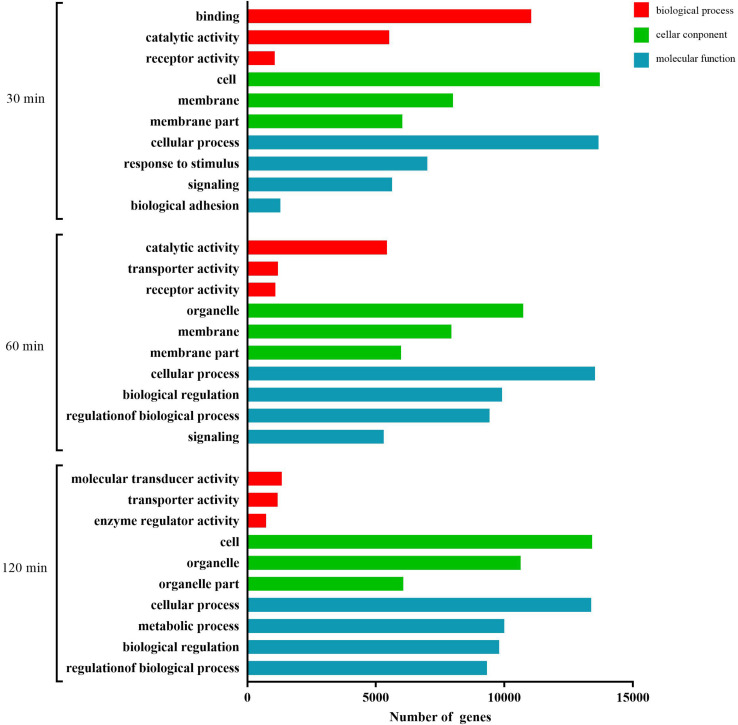
GO analysis of the predicted target genes with differentially expressed miRNAs during the process of FAdV-4 infection at three time points. The *X* axis shows the number of differentially expressed genes, and the *Y* axis shows the GO terms. All GO terms are grouped into three ontologies: red indicates biological processes, green indicates cellular components, and blue indicates molecular functions.

As shown in Figure 4, at 30 min, the most abundant GO terms were binding, catalytic activity, and receptor activity (biological process); cell, membrane, and membrane part (cellular component); and cellular process, response to stimulus, signaling, and biological adhesion (molecular function). At 60 min, the most abundant GO terms were catalytic activity, transporter activity, and receptor activity (biological process); organelle, membrane, and membrane part (cellular component); and molecular function. At 120 min, the most abundant GO terms were molecular transducer activity, transporter activity, and enzyme regulator activity (biological process); cell, organelle, and organelle part (cellular component); and cellular process, metabolic process, biological regulation, and regulation of biological process (molecular function).

**FIGURE 5:**
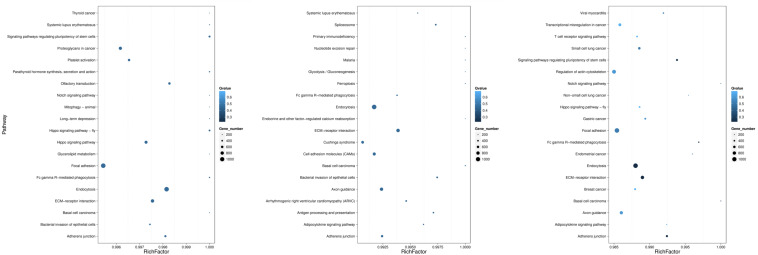
KEGG pathways related to the predicted target genes with differentially expressed miRNAs. The left, middle, and right panels show the KEGG enrichment analysis of differentially expressed miRNAs during the process of FAdV-4 infection at 30, 60, and 120 min. The Rich factor is the ratio of differentially expressed gene target gene numbers annotated in this pathway term to all gene numbers annotated in this pathway term. The greater the Rich factor is, the greater the degree of enrichment is. The *Q*-value is the corrected *P*-value and ranges from 0 to 1; the lower the *Q*-value is, the greater the level of enrichment is.

As shown in Figure 5, the 20 most prominent KEGG pathways were identified during the process of FAdV-4 infection at three time points. Interestingly, our data suggest that the miRNAs that were differentially expressed at all three time points are significantly associated with gene targets found in pathways that involve virus entry. Among the differentially expressed target genes at 30 min, most target genes were enriched in endocytosis, signaling pathways regulating the pluripotency of stem cells and the Hippo signaling pathway-fly. At 60 min, the target genes were enriched in the following pathways: endocytosis, extracellular matrix–receptor interaction, and axon guidance. The target genes that were changed at 120 min were mostly related to focal adhesion, regulation of actin cytoskeleton, and axon guidance.

### Construction and Sequencing Data Analysis of mRNA Libraries

**FIGURE 6:**
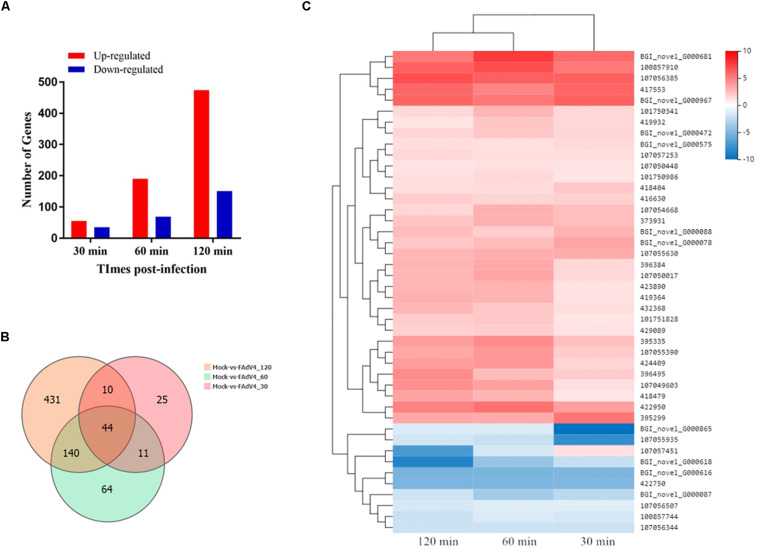
Transcriptome data profile and differential expression analysis. **(A)** Number of differentially expressed genes at three time points after FAdV-4 infection. **(B)** Venn diagram showing the unique and co-differentially expressed genes in response to FAdV-4 infection at three time points. **(C)** Hierarchical clustering analysis of 44 differentially expressed genes at all three time points. The color in the heat map represents the normalized isotig number. The color in the heat map represents the gene expression changes. Red indicates the upregulation of gene expression, blue indicates the downregulation of expression, the darker color indicates a notable degree of differential gene expression, and white indicates no activity.

Approximately, 44–49 million clean reads were sequenced and filtered from 12 cDNA libraries prepared from mock and infected cells. After filtering, the high-quality clean reads to the *Gallus gallus* genomes and all samples had mapping ratios from 85 to 89%. Moreover, 18,313 transcripts were identified in the RNA sequencing analysis. The identification of DEGs between the mock and the infected groups was based on a *q*-value threshold < 0.01 and | log2 (fold change)| > 1. In total, 725 DEGs were identified in the LMH cells after FAdV-4 infection at three time points (Figure 6A). Among these genes, 90, 259, and 625 DEGs were identified at 30, 60, and 120 min, respectively (Supplementary Tables S4–S6). A Venn diagram revealed that 44 genes were differentially expressed at all three time points (Figure 6B). By analyzing their expression, three differentially expressed clusters containing 44 genes were identified (Figure 6C). Gradual changes in gene expression were observed between 30, 60, and 120 min, and the left side of the hierarchical clustering gene tree allowed for the DEGs to be further classified. Thirty-five of the 44 genes were upregulated at all three time points. Among these 35 genes, CRK, SOCS1, SOCS3, EGR1, and CISH have been found to be involved in virus infection (Hrincius et al., 2010; Song et al., 2015; Wang X. et al., 2019).

### Functional Analyses of DEGs During FAdV-4 Infection

**FIGURE 7:**
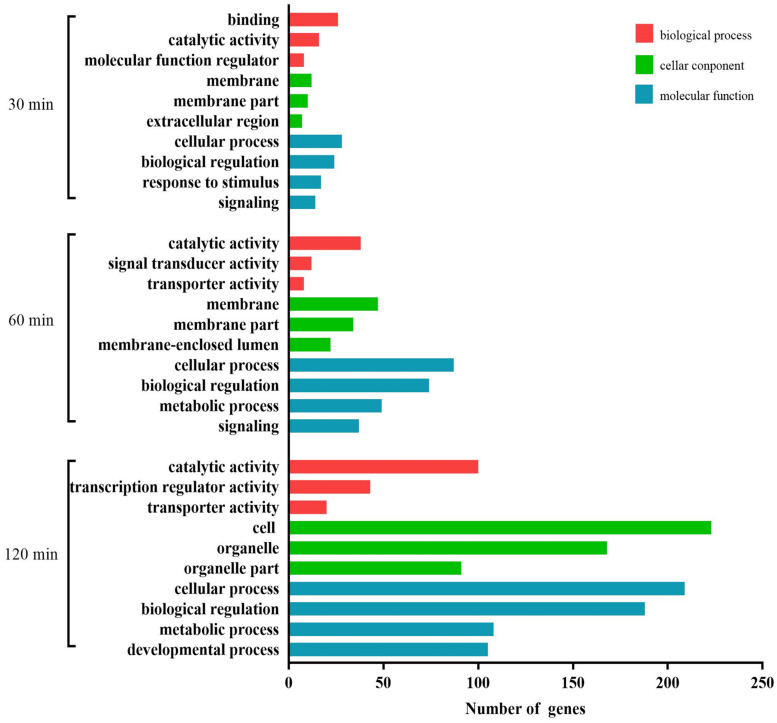
GO analysis of the differentially expressed mRNAs during the process of FAdV-4 infection at three time points. The *X* axis shows the number of differentially expressed genes, and the *Y* axis shows the GO terms. All GO terms are grouped into three ontologies: red indicates biological processes, green indicates cellular components, and blue indicates molecular functions.

A total of 725 DEGs were analyzed using the GO database at the three assay time points. The results of the functional analysis revealed a significant enrichment of GO terms, as shown in Figure 7. Binding, catalytic activity, and molecular function regulator (biological process); membrane, membrane part, and extracellular region (cellular component); and cellular process, biological regulation, response to stimulus, and signaling (molecular function) were the GO terms significantly enriched at 30 min. At 60 min, the most abundant GO terms were catalytic activity, signal transducer activity, and transporter activity (biological process); membrane, membrane part, and membrane-enclosed lumen (cellular component); and cellular process, biological regulation, metabolic process, and signaling (molecular function). At 120 min, the most abundant GO terms were catalytic activity, transcription regulator activity, and transporter activity (biological process); cell, organelle, and organelle part (cellular component); and cellular process, biological regulation, metabolic process, and developmental process (molecular function).

**FIGURE 8:**
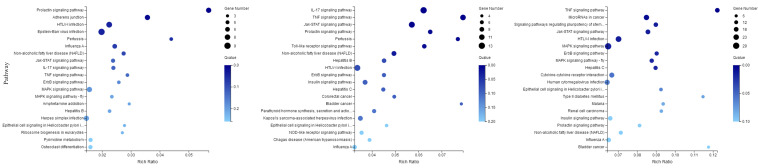
KEGG pathways related to the differentially expressed mRNAs. The left, middle, and right panels show the KEGG enrichment analysis of differentially expressed mRNAs during the process of FAdV-4 infection at 30, 60, and 120 min, respectively. The Rich factor is the ratio of differentially expressed gene numbers annotated in this pathway term to all gene numbers annotated in this pathway term. The greater the Rich factor is, the greater the degree of enrichment is. The *Q*-value is the corrected *P*-value and ranges from 0 to 1; the lower the *Q*-value is, the greater the level of enrichment is.

The DEGs were mapped to KEGG database analysis. As shown in Figure 8, the 20 most prominent KEGG pathways were identified at three time points. Among the differentially expressed genes at 30 min, most genes were enriched in the prolactin signaling pathway, adhered junction, and HTLV-I infection. At 60 min, the genes were enriched in the following pathways: IL-17 signaling pathway, TNF signaling pathway, and Toll-like receptor signaling pathway. The genes that were changed at 120 min were mostly related to the TNF signaling pathway, microRNAs in cancer, and MAPK signaling pathway.

### Integrative Genomic Analysis Associated With Negative miRNA–mRNA

For miRNAs to be functionally active, they need to be coexpressed with their target mRNAs. To further investigate miRNA–mRNA regulatory information after FAdV-4 infection, we calculated the Pearson data of miRNA–mRNA in the same sample. Generally, miRNAs negatively target genes. There were 41 negative miRNA–mRNA interactions with 37 miRNAs, and 11 mRNAs were differentially expressed at 30 min (Supplementary Table S7). A total of 154 negative miRNA–mRNA interactions with 61 miRNAs and 81 mRNAs were found at 60 min (Supplementary Table S8). Moreover, 85 miRNAs and 342 mRNAs with 661 negative miRNA–mRNA interactions were identified at 120 min (Supplementary Table S9). The top 10 miRNA–mRNA pairs at the three assay time points are shown in Table 2. Most miRNAs were associated with more than one mRNA target. For example, gga-miR-7475-5p associated with such mRNAs as TCF7L1, LFNG, ZIC3, and CREBZF. Moreover, most mRNAs are correlated with more than one miRNA, such as the host gene TCF7L1, which was targeted by ga-miR-7475-5p, novel_miR300, and novel_miR1128, and EVA1B, which was targeted by such miRNAs as gga-miR-128-2-5p, novel_miR202, and novel_miR975.

**TABLE 2:** The top 10 negative miRNA–mRNA pairs between mock and infected LMH cells at 30, 60, and 120 min.

miRNA_id	Target mRNA	miRNA fold change	Target mRNA fold change	Correlation (Pearson)	Time
novel_miR271	EGR1	–1.89	2.59	–0.86	30 min
novel_miR271	BTG2	–1.89	1.56	–0.85	
novel_miR872	FOS	–2.36	3.78	–0.82	
gga-miR-6651-5p	LOC100857744	3.75	–1.62	–0.76	
novel_miR735	FOS	–7.09	3.78	–0.72	
novel_miR1262	LOC100859032	–1.07	2.09	–0.71	
gga-miR-7475-5p	TCF7L1	3.03	–1.12	–0.70	
novel_miR1128	TCF7L1	7.67	–1.12	–0.69	
novel_miR898	DUSP1	–1.57	1.12	–0.69	
novel_miR271	IER5	–1.89	1.06	–0.69	
novel_miR271	EGR1	–6.57	3.09	–0.86	60 min
novel_miR271	BTG2	–6.57	2.29	–0.85	
novel_miR276	SERTAD2	–6.29	1.25	–0.82	
novel_miR120	BCL2L11	3.30	–1.49	–0.79	
novel_miR569	LOC107057304	–1.26	2.42	–0.77	
novel_miR820	BTG2	–6.43	2.29	–0.77	
novel_miR585	LOC101747455	5.53	–1.06	–0.77	
novel_miR975	EVA1B	–1.14	1.15	–0.77	
novel_miR261	LOC107056867	2.31	–2.36	–0.76	
gga-miR-6651-5p	LOC100857744	2.83	–1.46	–0.76	
gga-miR-128-2-5p	EVA1B	–1.09	1.40	–0.93	120 min
novel_miR909	CREBZF	3.28	–1.07	–0.89	
novel_miR326	HELZ2	–1.07	1.03	–0.89	
novel_miR1107	HIST1H103	8.76	–1.10	–0.87	
gga-miR-7475-5p	HIST1H103	4.05	–1.10	–0.87	
novel_miR636	LOC107052968	–1.02	2.02	–0.86	
novel_miR142	DENND3	–2.09	1.20	–0.86	
novel_miR326	EVA1A	–1.07	1.28	–0.85	
novel_miR1117	SLC25A25	–1.82	1.26	–0.85	
novel_miR1128	ZIC3	8.05	–1.17	–0.85	

### Functional Annotation and Pathways Affected by Relevant Negative miRNA–mRNA Correlations in LMH Cells

**FIGURE 9:**
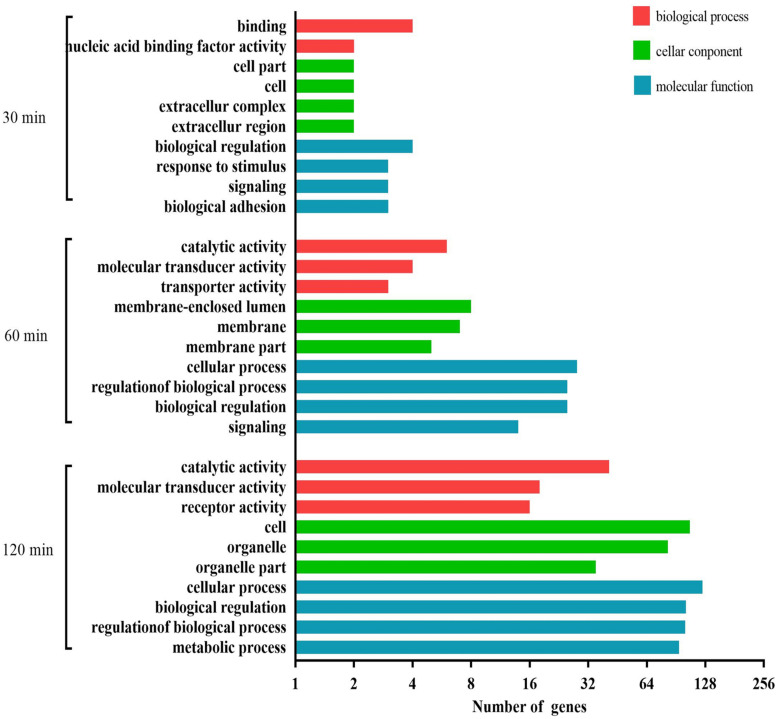
GO analysis for negatively correlated miRNA–mRNA during the process of FAdV-4 infection at three time points. The *X* axis shows the number of differentially expressed genes, and the *Y* axis shows the GO terms. All GO terms are grouped into three ontologies: red indicates biological processes, green indicates cellular components, and blue indicates molecular functions.

We carried out a GO analysis of the significantly negative miRNA–mRNA pairs at all three time points (Figure 9). Binding and nucleic acid binding transcription factor activity (biological process); extracellular region, extracellular complex, cell, and cell part (cellular component); and biological adhesion, signaling, response to stimulus, and biological regulation (molecular function) were the GO terms significantly enriched at 30 min. At 60 min, the most abundant GO terms were catalytic activity, transporter activity, and molecular transducer activity (biological process); membrane-enclosed lumen, membrane, and membrane part (cellular component); and cellular process, regulation of biological process, biological regulation, and signaling (molecular function). At 120 min, the most abundant GO terms were catalytic activity, molecular transducer activity, and receptor activity (biological process); cell, organelle, and organelle part (cellular component); and cellular process, biological regulation, regulation of biological process, and metabolic process (molecular function).

**FIGURE 10:**
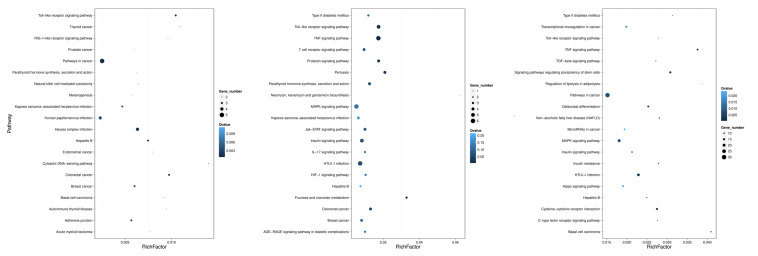
KEGG analysis for negatively correlated miRNA–mRNA. The left, middle, and right panels show the KEGG enrichment analysis of negatively correlated miRNA–mRNA during the process of FAdV-4 infection at 30, 60, and 120 min, respectively. The Rich factor is the ratio of differentially expressed gene numbers annotated in this pathway term to all gene numbers annotated in this pathway term. The greater the Rich factor is, the greater the degree of enrichment is. The *Q*-value is the corrected *P*-value and ranges from 0 to 1; the lower the *Q*-value is, the greater the level of enrichment is.

These miRNA–mRNA pairs were also were mapped to KEGG database analysis. As shown in Figure 10, the 20 most prominent KEGG pathways were identified at three time points. Among the differentially expressed miRNA–mRNA pairs at 30 min, most genes were pathways in cancer, herpes simplex infection, and the Toll-like receptor signaling pathway. At 60 min, the miRNA–mRNA pairs were enriched in the following pathways: the MAPK signaling pathway, the TNF signaling pathway, the Toll-like receptor signaling pathway, and HTLV-I infection. The miRNA–mRNA pairs that were changed at 120 min were mostly related to pathways in cancer, the TNF signaling pathway, HTLV-I infection, and the MAPK signaling pathway.

**FIGURE 11:**
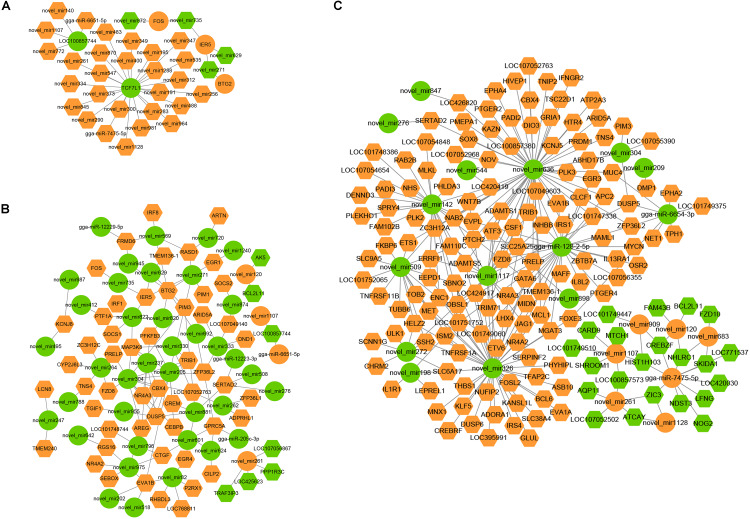
miRNA–mRNA negative correlation network in FAdV-4-infected LMH cells. **(A–C)** The negative miRNA–mRNA interactions from infected LMH cells at 30, 60, and 120 min. Orange indicates upregulation, and green indicates downregulation. Rhombuses denote mRNA, and circulars represent miRNA.

Interaction networks of the miRNAs and the target mRNAs were constructed (Figure 11). We found that several miRNAs and mRNAs played important roles in interaction networks, such as the host gene TCF7L1, which was targeted by 24 miRNAs at 30 min. Novel_miR205 correlated with 11 mRNAs at 60 min. At 120 min, novel_miR 636 correlated with 90 mRNAs, gga-miR-128-2-5p correlated with 46 mRNAs, and gga-miR-7475-5p correlated with 15 mRNAs.

### qPCR Validation of DEGs

Six DE miRNAs and nine DEGs among the negative miRNA–mRNA pairs were selected as target genes for qRT-PCR analysis. The changed patterns by qRT-PCR agreed well with the sequencing results (Table 3).

**TABLE 3:** Validation of the miRNA–mRNA pairs by qPCR.

miRNA_id	MiRNA-seq	Real-time PCR fold change	mRNA symbol	mRNA-seq fold change	Real-time PCR fold change
gga-miR-7475-5p	3.03	5.81	CREBZF	–1.07	–3.21
			TCF7L1	–1.12	–2.02
			LFNG	–1.59	–4.02
gga-miR-128-2-5p	–1.09	–1.51	OBSL1	1.69	1.82
			SOX4	1.08	3.28
gga-miR-205c-3p	–1.04	–2.61	FRMD6	1.85	2.20
gga-miR-12223-3p	–1.12	–2.32	SERTAD2	1.25	1.05
gga-miR-6654-3p	–2.63	–1.04	NFATC1	1.01	2.58
gga-miR-12245-3p	2.22	4.52	AQP11	–1.00	–2.23

### Validation of the miRNA–mRNA Interactions and Related Biological Function in FAdV-4 Entry

**FIGURE 12:**
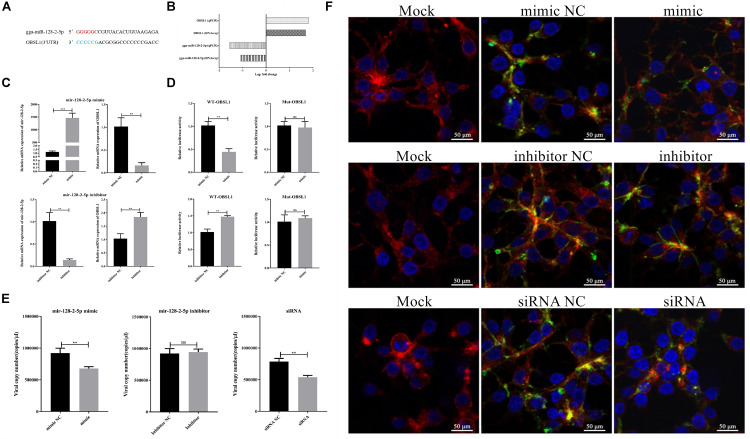
Effects of gga-miR-128-2-5p and OBSL1 on FAdV-4 entry into LMH cells. **(A)** Predicted binding sites of gga-miR-128-2-5p at the 3′UTR region in the OBSL1 sequence. The color-labeled sequence represents the complementary region between gga-miR-128-2-5p and the 3′UTR of OBSL1. **(B)** RNA-seq and RT-qPCR detected the expression changes of gga-miR-128-2-5p and OBSL1 during FAdV-4 infection. **(C)** RT-qPCR detected the expression regulation of OBSL1 by gga-miR-128-2-5p. The statistical analyses were performed in GraphPad Prism using unpaired two-tailed *t*-tests: **p < 0.01, ***p < 0.001. **(D)** A luciferase activity assay detected the expression regulation of OBSL1 by gga-miR-128-2-5p. The statistical analyses were performed in GraphPad Prism using unpaired two-tailed *t*-tests: **p < 0.01, and ns indicates no significant difference. **(E)** Quantitative analysis of the effects of gga-miR-128-2-5p and OBSL1 on FAdV-4 entry. The statistical analyses were performed in GraphPad Prism using unpaired two-tailed *t*-tests: **p < 0.01 and ns indicates no significant difference. **(F)** Confocal microscopy analysis of the effects of gga-miR-128-2-5p and OBSL1 on FAdV-4 entry.

To validate the correlations between the miRNAs and the mRNAs, the predicated bond between gga-miR-128-2-5p and OBSL1 (Figure 12A) was further studied. Both the RNA-seq and RT-qPCR results indicated that gga-miR-128-2-5p and its target gene OBSL1 had an inverse expression tendency in FAdV-4 infection (Figure 12B). The expression of OBSL1 in the LMH cells treated with gga-miR-128-2-5p mimics or inhibitors was quantified using RT-qPCR. Compared with the negative control, the overexpression or the interference with gga-miR-128-2-5p could significantly suppress or increase the expression of OBSL1 (Figure 12C). The luciferase assay confirmed the inverse relationship between gga-miR-128-2-5p and OBSL1 (Figure 12D). To investigate the biological function of gga-miR-128-2-5p in FAdV-4 entry, FAdV-4 proliferation in the LMH cells treated with gga-miR-128-2-5p mimics and inhibitors was detected. The virus copy number analysis indicated that gga-miR-128-2-5p mimics-treated and OBSL1 siRNA-treated cells showed inhibited FAdV-4 entry (Figure 12E). Similarly, CLSM revealed that gga-miR-128-2-5p mimics and OBSL1 siRNA treatment inhibit the expression of green fluorescent signals specific to FAdV-4 in the cell (Figure 12F). Therefore, FAdV-4 entry was inhibited in gga-miR-128-2-5p mimics-treated and OBSL1 siRNA-treated cells. Thus, gga-miR-128-2-5p regulated the expression of OBSL1, which plays a negative role in FAdV-4 entry.

## Discussion

It is important to understand the entry mechanism of FAdV-4 as virus entry into the host cells is the first critical step in the pathogenesis of infection. In this study, we investigated the changes in host miRNAs and mRNAs at the early stage of FAdV-4 infection and analyzed the interaction between miRNAs and mRNAs. As far as we know, this is the first study of host miRNA and mRNA variations in FAdV-4 infection.

In this study, we demonstrated that FAdV-4 uptake occurs within 60 min, which was consistent with the previous study (Bartlett et al., 2000). Furthermore, we identified three characteristic stages (30, 60, and 120 min) of FAdV-4 entry into the LMH cells and analyzed the dynamics of miRNA expression during viral infection. Our results indicate that several miRNAs are differentially regulated by FAdV-4 infection in the LMH cells *in vitro*, suggesting a different host response to FAdV-4. gga-miR-148a interacts with the SOCS3 gene and plays vital roles in the response to *Campylobacter jejuni* inoculation (Wang et al., 2018). gga-miR-15c was downregulated with AIV infection (Wang et al., 2012). Our results showed that gga-miR-148a-3p, gga-miR-148a-5p, and gga-miR-15c-3p were downregulated at all three time points. These miRNAs may be correlated with the host cell response to viral entry. In addition, our results show that several miRNAs were differentially expressed during FAdV-4 infection, such as gga-miR-1662, gga-miR-1454, and gga-miR-3538, which have been reported to play roles in virus and bacterial infection. As previously reported, gga-miR-1662 affects the immune responses in SE infection by targeting IL24 (Wu et al., 2017). It has been reported that gga-miR-1454 and gga-miR-3538 are highly expressed in IBV-infected chickens, and these two miRNAs are considered to play an important role in IBV–host interactions and the differing virulence of IBV strains (Yang et al., 2017). Interestingly, our results showed that these miRNAs are correlated with gene targets found in pathways that involve virus entry. The GO and KEGG pathway analysis of the differentially expressed miRNAs revealed that the target genes were involved in many signaling pathways, including endocytosis, binding, receptor activity, metabolic process, and biological regulation. These results together show the important role of host miRNAs in regulating FAdV-4 entry.

This study represents the first investigation of cell responses induced by infection with FAdV-4 at an early stage. Our study found a large quantity of mRNAs implicated in the early cellular responses to FAdV-4 infection. At all three time points, several genes were upregulated, including CRK, SOCS3, and EGR1. As previously reported, CRK contributes to efficient virus replication during the course of IAV infection (Hrincius et al., 2010). NDV infection activates the expression of SOCS3 through the MEK/ERK signaling pathway, which promotes virus replication (Wang X. et al., 2019). EGR1 facilitates enterovirus 71 replication by directly binding to viral genomic (Song et al., 2015). These genes perhaps correlated with the host cell response to viral entry, which may cause the virus to be recognized by the host immune system (Chovatiya and Medzhitov, 2014). During adenovirus infection, the viruses first adhere to the cell membrane and act as viral binding loci in conjunction with cell surface-specific structures (Wang X. et al., 2019). The GO analysis indicated that the DEGs at 30 min were mainly enriched in such processes as binding, adherence junction, and response to stimulus. In addition, during the entry process of the virus, regulation of cellular gene expression is likely to be triggered by the recognition of viral nucleic acids by Toll-like receptors (Bolduc et al., 2017). Interestingly, our results also showed that the DEGs were enriched in the Toll-like receptor signaling pathway at 60 min. As a matter of fact, the whole viral infection process is closely connected to “transport” (Smith and Helenius, 2004). At 120 min, most of the genes were involved in transporter activity, cellular process, and biological regulation. These results may suggest that FAdV-4 targeted a limited but specific set of host cellular genes during the early stages of infection.

To increase the reliability of the results, the correlations between the miRNA-seq and mRNA-seq were analyzed. The negatively correlated miRNA–mRNA pairs were our focal point (Zhang et al., 2015). Generally, one mRNA was also targeted by several miRNAs, and most miRNAs were correlated with more than one mRNA target. The interaction network analysis showed that several miRNAs and mRNAs played important roles in interaction networks. TCF7l1 is a member of the T cell factor, mediating the Wnt signaling pathway (Yuan et al., 2019). A previous study has shown evidence for the activation of Wnt signaling during adenovirus infection (Zhao et al., 2007). In the current study, such miRNAs as gga-miR-7475-5p, novel_miR1128, novel_miR488, and novel_miR283 were negatively correlated with TCF7l1 during FAdV-4 infection at 30 min, indicating that FAdV-4 may exploit Wnt pathways through TCF7l1 to facilitate virus entry. PIM3 is a family member of the PIM kinase family, and a study has shown that PIM kinases play an important role in the entry step of HCV infection (Park et al., 2015). MAP3K8 is a potential therapeutic target for diverse diseases, and some miRNAs repress HCV production by targeting MAP3K8 (Tsubota et al., 2014). In our study, we found that novel_miR205 was inversely correlated with PIM3 and MAP3K8 during FAdV-4 infection at 60 min. Novel_miR205 may play pivotal roles in FAdV-4 infection. In addition, gga-miR-128-2-5p was negatively correlated with OBSL1 and SOX4 during FAdV-4 infection at 120 min. OBSL1 encodes a cytoskeletal adaptor protein, which is required for human papillomavirus 16 (HPV16) endocytosis (Wustenhagen et al., 2016). SOX4 benefits from HBV replication by directly binding to the viral genome (Shang et al., 2015). Furthermore, gga-miR-7475-5p was negatively correlated with CREBZF and LFNG during FAdV-4 infection at 120 min; studies showed that these genes were all associated with the host cell response to viral infection (Smith et al., 2010; Mukherjee et al., 2014).

We focused on gga-miR-128-2-5p partly because its potential target gene OBSL1 is known to play a role in virus entry. In the current study, OBSL1 was predicted to be a potential gga-miR-128-2-5p target by the miRNA–target interaction network, and opposing expression patterns for gga-miR-128-2-5p and OBSL were identified. OBSL1 was first discovered in 2007; it is thought to act as a cytoskeletal adaptor protein, for example, in linking the internal cytoskeleton to the plasma membrane (Geisler et al., 2007). Moreover, OBSL1 was discovered as an interaction partner of the minor capsid protein L2 and was identified as a proviral host factor required for HPV16 endocytosis into target cells. In this study, OBSL1 siRNA-treated cells showed inhibited FAdV-4 entry. According to their correlation, gga-miR-128-2-5p may suppress OBSL1 expression *via* posttranscriptional gene silencing. Furthermore, the adenovirus enters target cells by receptor-mediated endocytosis (Graham et al., 1977); for example, EDSV enters DEF cells through clathrin-mediated endocytosis (Huang et al., 2015). These facts revealed that gga-miR-128-2-5p targets against FAdV-4 endocytosis by downregulating its target OBSL1 during virus invasion.

In conclusion, this report describes the first comprehensive genome-wide study during FAdV-4 entry. Our work suggests that analyzing the coexpression of miRNA and mRNA pairs enhances the power of RNA analysis. Unveiling differential miRNA–mRNA coexpression properties may help to understand the molecular mechanisms underlying the pathogenesis of FAdV-4.

## Data Availability Statement

The datasets generated for this study can be found in the gene expression omnibus (GEO). The accession code is PRJNA603161 (ID: 603161).

## Author Contributions

NW carried out the experiments, collected the data, and wrote the manuscript. BY performed the data analysis. BW, TW, and JG contributed to the experiments. JW and XQ conceived the study and participated in its design and coordination. All the authors reviewed the manuscript.

## Conflict of Interest

The authors declare that the research was conducted in the absence of any commercial or financial relationships that could be construed as a potential conflict of interest.

## Supplementary Material

FIGURE S1 Hierarchical clustering analysis of 784 differentially expressed miRNAs at three time points. The color in the heat map represents the gene expression changes. Red indicates the upregulation of gene expression, blue indicates the downregulation of expression, the darker color indicates a notable degree of differential gene expression, and white indicates no activity.

TABLE S1 Differentially expressed miRNAs between mock and infected LMHs at 30 min.

TABLE S2 Differentially expressed miRNAs between mock and infected LMHs at 60 min.

TABLE S3 Differentially expressed miRNAs between mock and infected LMHs at 120 min.

TABLE S4 Differentially expressed mRNAs between mock and infected LMHs at 30 min.

TABLE S5 Differentially expressed mRNAs between mock and infected LMHs at 60 min.

TABLE S6 Differentially expressed mRNAs between mock and infected LMHs at 120 min.

TABLE S7 The negative miRNA-mRNA pairs between mock and infected LMHs at 30 min.

TABLE S8 The negative miRNA-mRNA pairs between mock and infected LMHs at 60 min.

TABLE S9 The negative miRNA-mRNA pairs between mock and infected LMHs at 120 min.
