# Supplementary figures and images for: Interactions Among Expressed MicroRNAs and mRNAs in the Early Stages of Fowl Adenovirus Aerotype 4-Infected Leghorn Male Hepatocellular Cells
Source: Front Microbiol. 2020 May 19;11:831. doi: 10.3389/fmicb.2020.00831 (PMC7248314; doi:10.3389/fmicb.2020.00831)

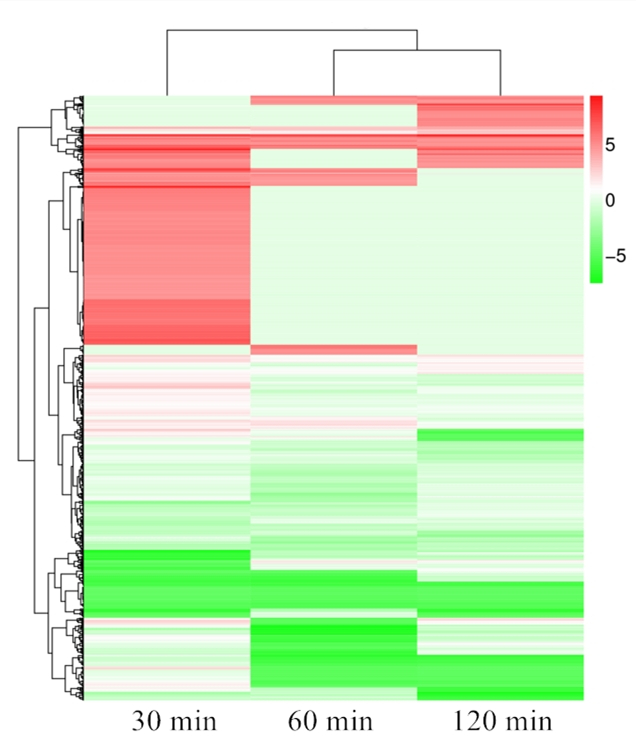

Supplement: FIGURE S1 — Hierarchical clustering analysis of 784 differentially expressed miRNAs at three time points. The color in the heat map represents the gene expression changes. Red indicates the upregulation of gene expression, blue indicates the downregulation of expression, the darker color indicates a notable degree of differential gene expression, and white indicates no activity. [file Data_Sheet_1.ZIP › supplementary files/S1.jpg]
