# Supplementary material for: Interactions Among Expressed MicroRNAs and mRNAs in the Early Stages of Fowl Adenovirus Aerotype 4-Infected Leghorn Male Hepatocellular Cells
Source: Front Microbiol. 2020 May 19;11:831. doi: 10.3389/fmicb.2020.00831 (PMC7248314; doi:10.3389/fmicb.2020.00831)
Supplement: FIGURE S1 — Hierarchical clustering analysis of 784 differentially expressed miRNAs at three time points. The color in the heat map represents the gene expression changes. Red indicates the upregulation of gene expression, blue indicates the downregulation of expression, the darker color indicates a notable degree of differential gene expression, and white indicates no activity. [file Data_Sheet_1.ZIP › supplementary files/Table S1.doc]

TABLE S1 Differentially expressed miRNAs between mock and infected LMHs at 30 min

| **miRNA id** | **Read count**  **(Mock)** | **Read count**  **(FAdV4_30)** | **log2 Ratio**  **(FAdV4_30/Mock)** | **Up down regulation** |
| --- | --- | --- | --- | --- |
| gga-miR-196-5p | 397740 | 708468 | 1.002009102 | UP |
| gga-miR-148b-3p | 381740 | 114741 | -1.565077299 | DOWN |
| gga-miR-148a-3p | 251503 | 69807 | -1.679999922 | DOWN |
| novel_mir1 | 28232 | 69001 | 1.458418005 | UP |
| novel_mir261 | 5956 | 28302 | 2.417620417 | UP |
| gga-miR-130a-3p | 40217 | 12557 | -1.510181567 | DOWN |
| gga-miR-1662 | 26619 | 10503 | -1.172522837 | DOWN |
| gga-miR-3532-3p | 10492 | 19755 | 1.082060203 | UP |
| novel_mir935 | 22 | 3797 | 7.600344735 | UP |
| novel_mir1223 | 4061 | 10462 | 1.534383812 | UP |
| gga-miR-301a-3p | 10354 | 4418 | -1.05959083 | DOWN |
| gga-miR-138-1-3p | 3555 | 6522 | 1.044596951 | UP |
| gga-miR-34b-5p | 1199 | 33 | -5.014089754 | DOWN |
| gga-miR-3534 | 1614 | 3612 | 1.331289384 | UP |
| novel_mir1186 | 1808 | 3805 | 1.242633846 | UP |
| gga-miR-138-2-3p | 1759 | 3344 | 1.095951434 | UP |
| novel_mir29 | 473 | 1390 | 1.724304864 | UP |
| gga-miR-365-3p | 2798 | 1184 | -1.071594812 | DOWN |
| novel_mir732 | 610 | 1490 | 1.457563252 | UP |
| gga-miR-12266-3p | 1118 | 285 | -1.802754294 | DOWN |
| gga-miR-1467-3p | 2141 | 890 | -1.097275485 | DOWN |
| gga-miR-34c-5p | 647 | 1413 | 1.296055918 | UP |
| gga-miR-12238-3p | 735 | 1450 | 1.149368815 | UP |
| novel_mir21 | 43 | 345 | 3.173319867 | UP |
| gga-miR-7475-5p | 49 | 356 | 3.030155656 | UP |
| novel_mir250 | 525 | 1117 | 1.258371928 | UP |
| novel_mir559 | 822 | 1499 | 1.035922154 | UP |
| novel_mir762 | 306 | 787 | 1.531964052 | UP |
| novel_mir1126 | 0 | 290 | 9.34904116 | UP |
| novel_mir1261 | 317 | 768 | 1.44575554 | UP |
| novel_mir395 | 0 | 252 | 9.146411993 | UP |
| novel_mir681 | 70 | 323 | 2.375239407 | UP |
| gga-miR-1731-5p | 765 | 285 | -1.255365759 | DOWN |
| novel_mir480 | 15 | 174 | 3.70518497 | UP |
| novel_mir149 | 309 | 58 | -2.244349963 | DOWN |
| novel_mir887 | 439 | 784 | 1.005764784 | UP |
| novel_mir780 | 135 | 369 | 1.619793479 | UP |
| gga-miR-20b-3p | 738 | 308 | -1.091558396 | DOWN |
| gga-miR-1813 | 258 | 536 | 1.223994005 | UP |
| gga-miR-212-5p | 282 | 567 | 1.176785642 | UP |
| novel_mir491 | 335 | 633 | 1.087176474 | UP |
| novel_mir339 | 33 | 174 | 2.567681446 | UP |
| gga-miR-1454 | 400 | 127 | -1.486039433 | DOWN |
| novel_mir45 | 319 | 594 | 1.066038577 | UP |
| gga-miR-301b-5p | 290 | 79 | -1.706996272 | DOWN |
| novel_mir909 | 171 | 375 | 1.30202634 | UP |
| novel_mir499 | 27 | 144 | 2.584169569 | UP |
| novel_mir1128 | 0 | 91 | 7.67692671 | UP |
| novel_mir199 | 38 | 161 | 2.252121434 | UP |
| gga-miR-6651-5p | 8 | 96 | 3.75409457 | UP |
| novel_mir873 | 0 | 89 | 7.644865501 | UP |
| novel_mir1107 | 0 | 87 | 7.612075565 | UP |
| novel_mir207 | 0 | 86 | 7.595396824 | UP |
| novel_mir618 | 66 | 203 | 1.790073867 | UP |
| novel_mir915 | 0 | 82 | 7.526684074 | UP |
| novel_mir1185 | 96 | 246 | 1.526684074 | UP |
| gga-miR-190a-3p | 466 | 195 | -1.087723761 | DOWN |
| novel_mir1071 | 101 | 251 | 1.482464141 | UP |
| novel_mir62 | 198 | 381 | 1.113422637 | UP |
| novel_mir47 | 153 | 319 | 1.229156841 | UP |
| novel_mir273 | 122 | 275 | 1.34168254 | UP |
| gga-miR-34c-3p | 113 | 262 | 1.382376109 | UP |
| novel_mir1026 | 0 | 72 | 7.339057071 | UP |
| novel_mir735 | 77 | 0 | -7.097654471 | DOWN |
| novel_mir284 | 0 | 69 | 7.277656526 | UP |
| novel_mir537 | 26 | 118 | 2.351335401 | UP |
| novel_mir1219 | 97 | 228 | 1.402109242 | UP |
| gga-miR-1612 | 213 | 381 | 1.008069637 | UP |
| novel_mir772 | 87 | 212 | 1.454109028 | UP |
| novel_mir257 | 10 | 79 | 3.150984723 | UP |
| novel_mir654 | 24 | 108 | 2.339057071 | UP |
| novel_mir800 | 25 | 109 | 2.293460205 | UP |
| novel_mir140 | 0 | 58 | 7.027113065 | UP |
| gga-miR-1754-3p | 146 | 286 | 1.139178847 | UP |
| gga-miR-12288-3p | 86 | 202 | 1.401078798 | UP |
| gga-miR-148a-5p | 248 | 89 | -1.30933081 | DOWN |
| novel_mir80 | 0 | 55 | 6.950491783 | UP |
| novel_mir1145 | 81 | 193 | 1.421739104 | UP |
| novel_mir1064 | 0 | 53 | 6.897052524 | UP |
| novel_mir684 | 11 | 74 | 2.919153817 | UP |
| novel_mir431 | 89 | 12 | -2.721638861 | DOWN |
| novel_mir836 | 57 | 0 | -6.663757945 | DOWN |
| novel_mir129 | 81 | 10 | -2.848789838 | DOWN |
| novel_mir135 | 55 | 0 | -6.612227644 | DOWN |
| novel_mir288 | 11 | 68 | 2.797163292 | UP |
| novel_mir204 | 0 | 45 | 6.660985166 | UP |
| novel_mir1115 | 10 | 65 | 2.869571788 | UP |
| novel_mir1100 | 0 | 44 | 6.628563688 | UP |
| novel_mir1016 | 0 | 44 | 6.628563688 | UP |
| novel_mir888 | 0 | 43 | 6.595396824 | UP |
| novel_mir1094 | 33 | 105 | 1.838983468 | UP |
| novel_mir91 | 60 | 146 | 1.452066033 | UP |
| novel_mir206 | 10 | 62 | 2.801400285 | UP |
| novel_mir933 | 12 | 65 | 2.606537382 | UP |
| gga-miR-1689-5p | 86 | 179 | 1.226683092 | UP |
| novel_mir570 | 44 | 0 | -6.290299549 | DOWN |
| novel_mir452 | 0 | 39 | 6.454534288 | UP |
| novel_mir1005 | 43 | 0 | -6.257132685 | DOWN |
| novel_mir1205 | 12 | 63 | 2.561449492 | UP |
| novel_mir333 | 69 | 10 | -2.617464292 | DOWN |
| novel_mir1021 | 0 | 38 | 6.417059583 | UP |
| novel_mir87 | 82 | 171 | 1.22943258 | UP |
| novel_mir561 | 52 | 128 | 1.468692351 | UP |
| novel_mir767 | 0 | 37 | 6.378585435 | UP |
| novel_mir536 | 0 | 37 | 6.378585435 | UP |
| novel_mir99 | 23 | 82 | 2.003122118 | UP |
| novel_mir497 | 0 | 36 | 6.339057071 | UP |
| novel_mir1230 | 0 | 36 | 6.339057071 | UP |
| novel_mir1070 | 0 | 36 | 6.339057071 | UP |
| novel_mir1238 | 10 | 56 | 2.654558897 | UP |
| novel_mir200 | 12 | 59 | 2.466812618 | UP |
| novel_mir1250 | 0 | 34 | 6.256594911 | UP |
| novel_mir413 | 38 | 0 | -6.078795444 | DOWN |
| novel_mir488 | 80 | 161 | 1.178120853 | UP |
| novel_mir743 | 37 | 0 | -6.040321296 | DOWN |
| novel_mir530 | 0 | 33 | 6.213526189 | UP |
| novel_mir321 | 0 | 33 | 6.213526189 | UP |
| novel_mir946 | 0 | 33 | 6.213526189 | UP |
| novel_mir279 | 0 | 32 | 6.16913207 | UP |
| novel_mir283 | 0 | 32 | 6.16913207 | UP |
| gga-miR-1739 | 100 | 185 | 1.05665734 | UP |
| novel_mir213 | 16 | 64 | 2.16913207 | UP |
| novel_mir533 | 82 | 161 | 1.142496943 | UP |
| novel_mir920 | 35 | 0 | -5.960150947 | DOWN |
| novel_mir1172 | 0 | 31 | 6.12332838 | UP |
| novel_mir471 | 0 | 31 | 6.12332838 | UP |
| novel_mir812 | 0 | 31 | 6.12332838 | UP |
| novel_mir175 | 0 | 31 | 6.12332838 | UP |
| novel_mir664 | 0 | 31 | 6.12332838 | UP |
| gga-miR-12284-5p | 26 | 80 | 1.790620446 | UP |
| novel_mir764 | 34 | 0 | -5.918330772 | DOWN |
| novel_mir194 | 13 | 57 | 2.301582366 | UP |
| novel_mir707 | 11 | 53 | 2.437620906 | UP |
| novel_mir1160 | 0 | 30 | 6.076022665 | UP |
| novel_mir1184 | 0 | 30 | 6.076022665 | UP |
| novel_mir626 | 0 | 29 | 6.027113065 | UP |
| novel_mir783 | 0 | 29 | 6.027113065 | UP |
| novel_mir676 | 32 | 0 | -5.83086793 | DOWN |
| novel_mir872 | 58 | 10 | -2.366920831 | DOWN |
| novel_mir192 | 0 | 28 | 5.976486992 | UP |
| novel_mir285 | 0 | 28 | 5.976486992 | UP |
| novel_mir214 | 0 | 28 | 5.976486992 | UP |
| novel_mir428 | 0 | 28 | 5.976486992 | UP |
| novel_mir1006 | 31 | 0 | -5.785064241 | DOWN |
| novel_mir653 | 31 | 0 | -5.785064241 | DOWN |
| novel_mir699 | 79 | 20 | -1.812720584 | DOWN |
| novel_mir1208 | 150 | 59 | -1.177043572 | DOWN |
| novel_mir459 | 10 | 48 | 2.432166475 | UP |
| novel_mir165 | 0 | 27 | 5.924019572 | UP |
| novel_mir267 | 0 | 27 | 5.924019572 | UP |
| novel_mir647 | 0 | 27 | 5.924019572 | UP |
| novel_mir1141 | 0 | 27 | 5.924019572 | UP |
| novel_mir270 | 0 | 27 | 5.924019572 | UP |
| novel_mir487 | 30 | 0 | -5.737758526 | DOWN |
| novel_mir151 | 29 | 0 | -5.688848926 | DOWN |
| novel_mir218 | 0 | 25 | 5.812988259 | UP |
| novel_mir454 | 0 | 25 | 5.812988259 | UP |
| novel_mir232 | 0 | 25 | 5.812988259 | UP |
| novel_mir242 | 28 | 0 | -5.638222852 | DOWN |
| novel_mir1081 | 28 | 0 | -5.638222852 | DOWN |
| novel_mir437 | 10 | 45 | 2.339057071 | UP |
| novel_mir789 | 18 | 59 | 1.881850118 | UP |
| novel_mir115 | 0 | 24 | 5.75409457 | UP |
| novel_mir866 | 0 | 24 | 5.75409457 | UP |
| novel_mir966 | 0 | 24 | 5.75409457 | UP |
| novel_mir1054 | 0 | 24 | 5.75409457 | UP |
| novel_mir851 | 0 | 24 | 5.75409457 | UP |
| novel_mir993 | 0 | 24 | 5.75409457 | UP |
| novel_mir1251 | 0 | 24 | 5.75409457 | UP |
| novel_mir162 | 0 | 24 | 5.75409457 | UP |
| novel_mir221 | 27 | 0 | -5.585755433 | DOWN |
| novel_mir884 | 27 | 0 | -5.585755433 | DOWN |
| novel_mir892 | 70 | 18 | -1.790225946 | DOWN |
| novel_mir136 | 59 | 13 | -2.013071262 | DOWN |
| novel_mir770 | 52 | 10 | -2.209379554 | DOWN |
| novel_mir970 | 0 | 23 | 5.692694026 | UP |
| novel_mir222 | 0 | 23 | 5.692694026 | UP |
| novel_mir860 | 0 | 23 | 5.692694026 | UP |
| novel_mir1031 | 38 | 87 | 1.364148052 | UP |
| novel_mir643 | 0 | 22 | 5.628563688 | UP |
| novel_mir260 | 0 | 22 | 5.628563688 | UP |
| novel_mir834 | 0 | 22 | 5.628563688 | UP |
| novel_mir816 | 0 | 22 | 5.628563688 | UP |
| novel_mir172 | 0 | 22 | 5.628563688 | UP |
| novel_mir956 | 0 | 22 | 5.628563688 | UP |
| novel_mir895 | 0 | 22 | 5.628563688 | UP |
| novel_mir176 | 0 | 22 | 5.628563688 | UP |
| novel_mir693 | 24 | 0 | -5.415830431 | DOWN |
| novel_mir939 | 24 | 0 | -5.415830431 | DOWN |
| novel_mir853 | 24 | 0 | -5.415830431 | DOWN |
| novel_mir324 | 24 | 0 | -5.415830431 | DOWN |
| novel_mir154 | 58 | 14 | -1.881494003 | DOWN |
| gga-miR-6572-3p | 1 | 22 | 4.628563688 | UP |
| novel_mir902 | 0 | 21 | 5.561449492 | UP |
| novel_mir584 | 0 | 21 | 5.561449492 | UP |
| novel_mir514 | 0 | 21 | 5.561449492 | UP |
| novel_mir230 | 0 | 21 | 5.561449492 | UP |
| novel_mir578 | 12 | 45 | 2.076022665 | UP |
| novel_mir351 | 41 | 89 | 1.287313496 | UP |
| novel_mir1284 | 23 | 0 | -5.354429886 | DOWN |
| novel_mir72 | 23 | 0 | -5.354429886 | DOWN |
| novel_mir470 | 23 | 62 | 1.599766424 | UP |
| novel_mir981 | 0 | 20 | 5.491060164 | UP |
| novel_mir782 | 0 | 20 | 5.491060164 | UP |
| novel_mir531 | 0 | 20 | 5.491060164 | UP |
| novel_mir476 | 0 | 20 | 5.491060164 | UP |
| novel_mir845 | 0 | 20 | 5.491060164 | UP |
| novel_mir990 | 17 | 52 | 1.782108946 | UP |
| novel_mir590 | 34 | 77 | 1.348455769 | UP |
| novel_mir90 | 22 | 0 | -5.290299549 | DOWN |
| novel_mir962 | 22 | 0 | -5.290299549 | DOWN |
| novel_mir1240 | 22 | 0 | -5.290299549 | DOWN |
| novel_mir779 | 22 | 0 | -5.290299549 | DOWN |
| novel_mir210 | 22 | 0 | -5.290299549 | DOWN |
| novel_mir164 | 22 | 0 | -5.290299549 | DOWN |
| novel_mir663 | 45 | 92 | 1.200840929 | UP |
| gga-miR-1591-3p | 52 | 101 | 1.126903834 | UP |
| novel_mir256 | 0 | 19 | 5.417059583 | UP |
| novel_mir696 | 0 | 19 | 5.417059583 | UP |
| novel_mir1242 | 0 | 19 | 5.417059583 | UP |
| novel_mir686 | 0 | 19 | 5.417059583 | UP |
| novel_mir1137 | 0 | 19 | 5.417059583 | UP |
| novel_mir629 | 46 | 10 | -2.032501792 | DOWN |
| novel_mir765 | 21 | 0 | -5.223185353 | DOWN |
| novel_mir356 | 21 | 0 | -5.223185353 | DOWN |
| novel_mir183 | 11 | 40 | 2.031628546 | UP |
| novel_mir678 | 11 | 40 | 2.031628546 | UP |
| novel_mir400 | 14 | 45 | 1.853630244 | UP |
| novel_mir940 | 0 | 18 | 5.339057071 | UP |
| novel_mir416 | 0 | 18 | 5.339057071 | UP |
| novel_mir1014 | 0 | 18 | 5.339057071 | UP |
| novel_mir1255 | 0 | 18 | 5.339057071 | UP |
| novel_mir429 | 0 | 18 | 5.339057071 | UP |
| novel_mir815 | 0 | 18 | 5.339057071 | UP |
| novel_mir481 | 0 | 18 | 5.339057071 | UP |
| novel_mir641 | 20 | 0 | -5.152796025 | DOWN |
| novel_mir715 | 20 | 0 | -5.152796025 | DOWN |
| gga-miR-1722-5p | 98 | 39 | -1.160175556 | DOWN |
| novel_mir79 | 32 | 71 | 1.318879189 | UP |
| gga-miR-7438-5p | 30 | 4 | -2.737758526 | DOWN |
| novel_mir89 | 102 | 42 | -1.11097585 | DOWN |
| novel_mir271 | 46 | 11 | -1.894998268 | DOWN |
| novel_mir1007 | 0 | 17 | 5.256594911 | UP |
| novel_mir182 | 0 | 17 | 5.256594911 | UP |
| novel_mir462 | 0 | 17 | 5.256594911 | UP |
| novel_mir528 | 0 | 17 | 5.256594911 | UP |
| novel_mir573 | 0 | 17 | 5.256594911 | UP |
| novel_mir430 | 0 | 17 | 5.256594911 | UP |
| novel_mir858 | 0 | 17 | 5.256594911 | UP |
| novel_mir563 | 0 | 17 | 5.256594911 | UP |
| novel_mir1157 | 14 | 43 | 1.788041902 | UP |
| novel_mir224 | 11 | 38 | 1.957627964 | UP |
| novel_mir466 | 0 | 16 | 5.16913207 | UP |
| novel_mir227 | 0 | 16 | 5.16913207 | UP |
| novel_mir1118 | 0 | 16 | 5.16913207 | UP |
| novel_mir1155 | 0 | 16 | 5.16913207 | UP |
| novel_mir833 | 0 | 16 | 5.16913207 | UP |
| novel_mir571 | 0 | 16 | 5.16913207 | UP |
| novel_mir307 | 0 | 16 | 5.16913207 | UP |
| novel_mir591 | 0 | 16 | 5.16913207 | UP |
| novel_mir1008 | 0 | 16 | 5.16913207 | UP |
| gga-miR-1354 | 55 | 98 | 1.0024822 | UP |
| novel_mir168 | 10 | 35 | 1.976486992 | UP |
| novel_mir181 | 10 | 35 | 1.976486992 | UP |
| novel_mir277 | 0 | 15 | 5.076022665 | UP |
| novel_mir961 | 0 | 15 | 5.076022665 | UP |
| novel_mir697 | 0 | 15 | 5.076022665 | UP |
| novel_mir954 | 0 | 15 | 5.076022665 | UP |
| novel_mir1228 | 0 | 15 | 5.076022665 | UP |
| novel_mir574 | 0 | 15 | 5.076022665 | UP |
| novel_mir823 | 0 | 15 | 5.076022665 | UP |
| novel_mir1119 | 0 | 15 | 5.076022665 | UP |
| novel_mir1175 | 0 | 15 | 5.076022665 | UP |
| novel_mir1181 | 0 | 15 | 5.076022665 | UP |
| novel_mir785 | 0 | 15 | 5.076022665 | UP |
| novel_mir1135 | 17 | 0 | -4.918330772 | DOWN |
| gga-miR-15c-3p | 81 | 32 | -1.170717933 | DOWN |
| novel_mir116 | 77 | 30 | -1.190763875 | DOWN |
| novel_mir784 | 25 | 56 | 1.332630802 | UP |
| novel_mir619 | 16 | 0 | -4.83086793 | DOWN |
| novel_mir1248 | 16 | 0 | -4.83086793 | DOWN |
| novel_mir868 | 16 | 0 | -4.83086793 | DOWN |
| novel_mir161 | 16 | 0 | -4.83086793 | DOWN |
| novel_mir365 | 64 | 23 | -1.307305974 | DOWN |
| novel_mir995 | 0 | 14 | 4.976486992 | UP |
| novel_mir1044 | 0 | 14 | 4.976486992 | UP |
| novel_mir1010 | 0 | 14 | 4.976486992 | UP |
| novel_mir680 | 0 | 14 | 4.976486992 | UP |
| novel_mir191 | 0 | 14 | 4.976486992 | UP |
| novel_mir507 | 0 | 14 | 4.976486992 | UP |
| novel_mir504 | 0 | 14 | 4.976486992 | UP |
| novel_mir746 | 0 | 14 | 4.976486992 | UP |
| novel_mir1165 | 0 | 14 | 4.976486992 | UP |
| novel_mir824 | 0 | 14 | 4.976486992 | UP |
| novel_mir535 | 0 | 14 | 4.976486992 | UP |
| novel_mir433 | 0 | 14 | 4.976486992 | UP |
| gga-miR-1673 | 13 | 38 | 1.716619865 | UP |
| novel_mir456 | 55 | 18 | -1.442302642 | DOWN |
| novel_mir911 | 12 | 36 | 1.75409457 | UP |
| novel_mir898 | 47 | 14 | -1.57810186 | DOWN |
| novel_mir512 | 78 | 32 | -1.116270149 | DOWN |
| novel_mir146 | 81 | 34 | -1.083255092 | DOWN |
| novel_mir564 | 15 | 0 | -4.737758526 | DOWN |
| novel_mir901 | 15 | 0 | -4.737758526 | DOWN |
| novel_mir446 | 15 | 0 | -4.737758526 | DOWN |
| novel_mir568 | 10 | 32 | 1.847203975 | UP |
| gga-miR-12223-3p | 74 | 30 | -1.1334307 | DOWN |
| novel_mir513 | 0 | 13 | 4.869571788 | UP |
| novel_mir421 | 0 | 13 | 4.869571788 | UP |
| novel_mir885 | 0 | 13 | 4.869571788 | UP |
| novel_mir1040 | 0 | 13 | 4.869571788 | UP |
| novel_mir612 | 0 | 13 | 4.869571788 | UP |
| novel_mir567 | 0 | 13 | 4.869571788 | UP |
| novel_mir870 | 0 | 13 | 4.869571788 | UP |
| novel_mir774 | 0 | 13 | 4.869571788 | UP |
| novel_mir453 | 0 | 13 | 4.869571788 | UP |
| novel_mir1283 | 0 | 13 | 4.869571788 | UP |
| novel_mir1020 | 0 | 13 | 4.869571788 | UP |
| novel_mir827 | 0 | 13 | 4.869571788 | UP |
| novel_mir1060 | 0 | 13 | 4.869571788 | UP |
| novel_mir875 | 0 | 13 | 4.869571788 | UP |
| novel_mir556 | 0 | 13 | 4.869571788 | UP |
| novel_mir1234 | 0 | 13 | 4.869571788 | UP |
| novel_mir904 | 0 | 13 | 4.869571788 | UP |
| novel_mir547 | 0 | 13 | 4.869571788 | UP |
| novel_mir996 | 0 | 13 | 4.869571788 | UP |
| novel_mir760 | 0 | 13 | 4.869571788 | UP |
| novel_mir620 | 0 | 13 | 4.869571788 | UP |
| novel_mir336 | 0 | 13 | 4.869571788 | UP |
| novel_mir189 | 0 | 13 | 4.869571788 | UP |
| gga-miR-1669 | 4 | 21 | 2.561449492 | UP |
| novel_mir667 | 43 | 77 | 1.009653856 | UP |
| novel_mir1143 | 11 | 33 | 1.75409457 | UP |
| novel_mir825 | 14 | 0 | -4.638222852 | DOWN |
| novel_mir394 | 14 | 0 | -4.638222852 | DOWN |
| novel_mir1222 | 14 | 0 | -4.638222852 | DOWN |
| novel_mir718 | 14 | 0 | -4.638222852 | DOWN |
| novel_mir714 | 14 | 0 | -4.638222852 | DOWN |
| novel_mir523 | 14 | 0 | -4.638222852 | DOWN |
| novel_mir701 | 14 | 0 | -4.638222852 | DOWN |
| novel_mir581 | 38 | 70 | 1.050487573 | UP |
| gga-miR-6677-5p | 50 | 17 | -1.387261279 | DOWN |
| novel_mir117 | 26 | 54 | 1.223579854 | UP |
| novel_mir483 | 0 | 12 | 4.75409457 | UP |
| novel_mir740 | 0 | 12 | 4.75409457 | UP |
| novel_mir1202 | 0 | 12 | 4.75409457 | UP |
| novel_mir773 | 0 | 12 | 4.75409457 | UP |
| novel_mir700 | 0 | 12 | 4.75409457 | UP |
| novel_mir754 | 0 | 12 | 4.75409457 | UP |
| novel_mir187 | 0 | 12 | 4.75409457 | UP |
| novel_mir622 | 0 | 12 | 4.75409457 | UP |
| novel_mir634 | 0 | 12 | 4.75409457 | UP |
| novel_mir265 | 0 | 12 | 4.75409457 | UP |
| novel_mir713 | 0 | 12 | 4.75409457 | UP |
| novel_mir925 | 0 | 12 | 4.75409457 | UP |
| novel_mir719 | 0 | 12 | 4.75409457 | UP |
| novel_mir617 | 0 | 12 | 4.75409457 | UP |
| novel_mir742 | 0 | 12 | 4.75409457 | UP |
| novel_mir408 | 0 | 12 | 4.75409457 | UP |
| novel_mir1200 | 0 | 12 | 4.75409457 | UP |
| novel_mir193 | 0 | 12 | 4.75409457 | UP |
| novel_mir223 | 0 | 12 | 4.75409457 | UP |
| novel_mir778 | 0 | 12 | 4.75409457 | UP |
| novel_mir857 | 0 | 12 | 4.75409457 | UP |
| novel_mir562 | 0 | 12 | 4.75409457 | UP |
| novel_mir610 | 0 | 12 | 4.75409457 | UP |
| novel_mir795 | 0 | 12 | 4.75409457 | UP |
| novel_mir238 | 0 | 12 | 4.75409457 | UP |
| novel_mir1173 | 0 | 12 | 4.75409457 | UP |
| novel_mir724 | 0 | 12 | 4.75409457 | UP |
| novel_mir1194 | 0 | 12 | 4.75409457 | UP |
| novel_mir410 | 0 | 12 | 4.75409457 | UP |
| novel_mir119 | 41 | 73 | 1.001404624 | UP |
| novel_mir1236 | 44 | 14 | -1.482944627 | DOWN |
| gga-miR-132b-5p | 28 | 56 | 1.16913207 | UP |
| novel_mir1146 | 38 | 11 | -1.619363825 | DOWN |
| novel_mir1274 | 10 | 30 | 1.75409457 | UP |
| gga-miR-6542-5p | 7 | 25 | 2.005633337 | UP |
| novel_mir999 | 71 | 30 | -1.073724454 | DOWN |
| novel_mir1036 | 66 | 27 | -1.120374548 | DOWN |
| gga-miR-6649-5p | 12 | 33 | 1.628563688 | UP |
| novel_mir659 | 13 | 0 | -4.531307649 | DOWN |
| novel_mir838 | 13 | 0 | -4.531307649 | DOWN |
| novel_mir293 | 13 | 0 | -4.531307649 | DOWN |
| novel_mir805 | 13 | 0 | -4.531307649 | DOWN |
| novel_mir439 | 13 | 0 | -4.531307649 | DOWN |
| novel_mir1256 | 24 | 50 | 1.228025759 | UP |
| novel_mir1220 | 25 | 51 | 1.197701222 | UP |
| novel_mir310 | 0 | 11 | 4.628563688 | UP |
| novel_mir572 | 0 | 11 | 4.628563688 | UP |
| novel_mir731 | 0 | 11 | 4.628563688 | UP |
| novel_mir1182 | 0 | 11 | 4.628563688 | UP |
| novel_mir444 | 0 | 11 | 4.628563688 | UP |
| novel_mir281 | 0 | 11 | 4.628563688 | UP |
| novel_mir621 | 0 | 11 | 4.628563688 | UP |
| novel_mir1206 | 0 | 11 | 4.628563688 | UP |
| novel_mir545 | 0 | 11 | 4.628563688 | UP |
| novel_mir1224 | 0 | 11 | 4.628563688 | UP |
| novel_mir1221 | 0 | 11 | 4.628563688 | UP |
| novel_mir253 | 0 | 11 | 4.628563688 | UP |
| novel_mir905 | 0 | 11 | 4.628563688 | UP |
| novel_mir597 | 0 | 11 | 4.628563688 | UP |
| novel_mir1063 | 0 | 11 | 4.628563688 | UP |
| novel_mir134 | 0 | 11 | 4.628563688 | UP |
| novel_mir318 | 0 | 11 | 4.628563688 | UP |
| novel_mir173 | 0 | 11 | 4.628563688 | UP |
| novel_mir366 | 0 | 11 | 4.628563688 | UP |
| novel_mir726 | 0 | 11 | 4.628563688 | UP |
| novel_mir290 | 0 | 11 | 4.628563688 | UP |
| novel_mir644 | 0 | 11 | 4.628563688 | UP |
| novel_mir360 | 0 | 11 | 4.628563688 | UP |
| novel_mir1140 | 0 | 11 | 4.628563688 | UP |
| novel_mir1111 | 0 | 11 | 4.628563688 | UP |
| novel_mir775 | 0 | 11 | 4.628563688 | UP |
| novel_mir968 | 0 | 11 | 4.628563688 | UP |
| novel_mir1189 | 0 | 11 | 4.628563688 | UP |
| novel_mir638 | 0 | 11 | 4.628563688 | UP |
| novel_mir308 | 0 | 11 | 4.628563688 | UP |
| novel_mir669 | 0 | 11 | 4.628563688 | UP |
| novel_mir1169 | 0 | 11 | 4.628563688 | UP |
| novel_mir942 | 0 | 11 | 4.628563688 | UP |
| novel_mir171 | 0 | 11 | 4.628563688 | UP |
| novel_mir883 | 0 | 11 | 4.628563688 | UP |
| novel_mir983 | 0 | 11 | 4.628563688 | UP |
| novel_mir352 | 0 | 11 | 4.628563688 | UP |
| novel_mir399 | 0 | 11 | 4.628563688 | UP |
| novel_mir910 | 0 | 11 | 4.628563688 | UP |
| novel_mir369 | 0 | 11 | 4.628563688 | UP |
| novel_mir393 | 0 | 11 | 4.628563688 | UP |
| novel_mir258 | 0 | 11 | 4.628563688 | UP |
| novel_mir964 | 0 | 11 | 4.628563688 | UP |
| novel_mir1120 | 0 | 11 | 4.628563688 | UP |
| novel_mir932 | 35 | 10 | -1.638222852 | DOWN |
| gga-miR-7467-3p | 33 | 9 | -1.705337048 | DOWN |
| novel_mir198 | 27 | 53 | 1.142165022 | UP |
| novel_mir1187 | 12 | 0 | -4.415830431 | DOWN |
| novel_mir633 | 12 | 0 | -4.415830431 | DOWN |
| novel_mir977 | 12 | 0 | -4.415830431 | DOWN |
| novel_mir576 | 12 | 0 | -4.415830431 | DOWN |
| novel_mir177 | 12 | 0 | -4.415830431 | DOWN |
| novel_mir1037 | 12 | 0 | -4.415830431 | DOWN |
| novel_mir1102 | 12 | 0 | -4.415830431 | DOWN |
| novel_mir1057 | 12 | 0 | -4.415830431 | DOWN |
| novel_mir828 | 12 | 0 | -4.415830431 | DOWN |
| novel_mir792 | 12 | 0 | -4.415830431 | DOWN |
| novel_mir936 | 12 | 0 | -4.415830431 | DOWN |
| novel_mir1091 | 12 | 0 | -4.415830431 | DOWN |
| novel_mir1030 | 45 | 16 | -1.322721027 | DOWN |
| gga-miR-205c-5p | 50 | 19 | -1.226796607 | DOWN |
| novel_mir992 | 25 | 49 | 1.139985724 | UP |
| novel_mir914 | 25 | 49 | 1.139985724 | UP |
| novel_mir532 | 0 | 10 | 4.491060164 | UP |
| novel_mir862 | 0 | 10 | 4.491060164 | UP |
| novel_mir1281 | 0 | 10 | 4.491060164 | UP |
| novel_mir355 | 0 | 10 | 4.491060164 | UP |
| novel_mir347 | 0 | 10 | 4.491060164 | UP |
| novel_mir1244 | 0 | 10 | 4.491060164 | UP |
| novel_mir195 | 0 | 10 | 4.491060164 | UP |
| novel_mir349 | 0 | 10 | 4.491060164 | UP |
| novel_mir338 | 0 | 10 | 4.491060164 | UP |
| novel_mir474 | 0 | 10 | 4.491060164 | UP |
| novel_mir373 | 0 | 10 | 4.491060164 | UP |
| novel_mir378 | 0 | 10 | 4.491060164 | UP |
| novel_mir691 | 0 | 10 | 4.491060164 | UP |
| novel_mir354 | 0 | 10 | 4.491060164 | UP |
| novel_mir282 | 0 | 10 | 4.491060164 | UP |
| novel_mir334 | 0 | 10 | 4.491060164 | UP |
| novel_mir300 | 0 | 10 | 4.491060164 | UP |
| novel_mir1151 | 0 | 10 | 4.491060164 | UP |
| novel_mir332 | 0 | 10 | 4.491060164 | UP |
| novel_mir906 | 0 | 10 | 4.491060164 | UP |
| novel_mir1092 | 0 | 10 | 4.491060164 | UP |
| novel_mir346 | 0 | 10 | 4.491060164 | UP |
| novel_mir606 | 0 | 10 | 4.491060164 | UP |
| novel_mir166 | 0 | 10 | 4.491060164 | UP |
| novel_mir312 | 0 | 10 | 4.491060164 | UP |
| novel_mir1288 | 0 | 10 | 4.491060164 | UP |
| novel_mir534 | 0 | 10 | 4.491060164 | UP |
| novel_mir1183 | 0 | 10 | 4.491060164 | UP |
| novel_mir358 | 0 | 10 | 4.491060164 | UP |
| novel_mir1229 | 0 | 10 | 4.491060164 | UP |
| novel_mir382 | 0 | 10 | 4.491060164 | UP |
| novel_mir328 | 0 | 10 | 4.491060164 | UP |
| novel_mir392 | 0 | 10 | 4.491060164 | UP |
| novel_mir1068 | 0 | 10 | 4.491060164 | UP |
| novel_mir389 | 0 | 10 | 4.491060164 | UP |
| novel_mir1052 | 0 | 10 | 4.491060164 | UP |
| novel_mir1257 | 0 | 10 | 4.491060164 | UP |
| novel_mir1080 | 0 | 10 | 4.491060164 | UP |
| novel_mir345 | 0 | 10 | 4.491060164 | UP |
| novel_mir871 | 0 | 10 | 4.491060164 | UP |
| novel_mir371 | 0 | 10 | 4.491060164 | UP |
| novel_mir1170 | 0 | 10 | 4.491060164 | UP |
| novel_mir180 | 0 | 10 | 4.491060164 | UP |
| novel_mir1282 | 0 | 10 | 4.491060164 | UP |
| novel_mir120 | 11 | 29 | 1.567681446 | UP |
| gga-miR-3528 | 6 | 21 | 1.976486992 | UP |
| novel_mir672 | 33 | 10 | -1.553333955 | DOWN |
| novel_mir203 | 33 | 10 | -1.553333955 | DOWN |
| novel_mir1027 | 11 | 0 | -4.290299549 | DOWN |
| novel_mir794 | 11 | 0 | -4.290299549 | DOWN |
| novel_mir506 | 11 | 0 | -4.290299549 | DOWN |
| novel_mir1163 | 11 | 0 | -4.290299549 | DOWN |
| novel_mir758 | 11 | 0 | -4.290299549 | DOWN |
| novel_mir818 | 11 | 0 | -4.290299549 | DOWN |
| novel_mir1013 | 11 | 0 | -4.290299549 | DOWN |
| novel_mir766 | 11 | 0 | -4.290299549 | DOWN |
| novel_mir322 | 11 | 0 | -4.290299549 | DOWN |
| novel_mir679 | 11 | 0 | -4.290299549 | DOWN |
| novel_mir361 | 11 | 0 | -4.290299549 | DOWN |
| novel_mir494 | 11 | 0 | -4.290299549 | DOWN |
| novel_mir368 | 11 | 0 | -4.290299549 | DOWN |
| novel_mir485 | 11 | 0 | -4.290299549 | DOWN |
| novel_mir645 | 11 | 0 | -4.290299549 | DOWN |
| novel_mir139 | 11 | 0 | -4.290299549 | DOWN |
| novel_mir1033 | 11 | 0 | -4.290299549 | DOWN |
| novel_mir982 | 11 | 0 | -4.290299549 | DOWN |
| novel_mir1002 | 11 | 0 | -4.290299549 | DOWN |
| novel_mir179 | 11 | 0 | -4.290299549 | DOWN |
| novel_mir1262 | 57 | 24 | -1.078795444 | DOWN |
