# Supplementary material for: Interactions Among Expressed MicroRNAs and mRNAs in the Early Stages of Fowl Adenovirus Aerotype 4-Infected Leghorn Male Hepatocellular Cells
Source: Front Microbiol. 2020 May 19;11:831. doi: 10.3389/fmicb.2020.00831 (PMC7248314; doi:10.3389/fmicb.2020.00831)
Supplement: FIGURE S1 — Hierarchical clustering analysis of 784 differentially expressed miRNAs at three time points. The color in the heat map represents the gene expression changes. Red indicates the upregulation of gene expression, blue indicates the downregulation of expression, the darker color indicates a notable degree of differential gene expression, and white indicates no activity. [file Data_Sheet_1.ZIP › supplementary files/Table S2.doc]

TABLE S2 Differentially expressed miRNAs between mock and infected LMHs at 60 min

| **miRNA id** | **Read count(Mock)** | **Read count(FAdV4_60)** | **log2Ratio**  **(FAdV4_60/Mock)** | **Up down regulation** |
| --- | --- | --- | --- | --- |
| gga-let-7l-5p | 111703 | 28903 | -1.997253278 | DOWN |
| gga-miR-130a-3p | 40217 | 16279 | -1.351669885 | DOWN |
| gga-miR-148a-3p | 251503 | 88321 | -1.556623693 | DOWN |
| gga-miR-148b-3p | 381740 | 151903 | -1.376316499 | DOWN |
| gga-miR-1662 | 26619 | 13071 | -1.072963352 | DOWN |
| gga-miR-1674 | 17196 | 8493 | -1.064603357 | DOWN |
| gga-miR-21-3p | 13208 | 6465 | -1.077566247 | DOWN |
| novel_mir1223 | 4061 | 9611 | 1.195975024 | UP |
| novel_mir261 | 5956 | 30573 | 2.312965969 | UP |
| novel_mir935 | 22 | 2349 | 6.691522883 | UP |
| novel_mir984 | 2649 | 941 | -1.540057712 | DOWN |
| gga-miR-106-3p | 4218 | 1993 | -1.128493881 | DOWN |
| novel_mir9 | 3160 | 1435 | -1.185750318 | DOWN |
| gga-miR-1467-3p | 2141 | 991 | -1.15820433 | DOWN |
| gga-miR-429-5p | 2191 | 1041 | -1.120495912 | DOWN |
| gga-miR-7475-5p | 49 | 448 | 3.145768582 | UP |
| novel_mir909 | 171 | 705 | 1.996750436 | UP |
| gga-let-7j-3p | 322 | 16 | -4.377793375 | DOWN |
| novel_mir21 | 43 | 397 | 3.159853946 | UP |
| gga-miR-12266-3p | 1118 | 455 | -1.343858234 | DOWN |
| gga-miR-12229-3p | 757 | 286 | -1.45115465 | DOWN |
| gga-miR-1454 | 400 | 87 | -2.24778919 | DOWN |
| gga-miR-212-5p | 282 | 716 | 1.297387928 | UP |
| gga-miR-20b-3p | 738 | 324 | -1.2345035 | DOWN |
| novel_mir149 | 309 | 81 | -1.978489521 | DOWN |
| novel_mir49 | 283 | 85 | -1.782143803 | DOWN |
| gga-miR-301b-5p | 290 | 90 | -1.73493249 | DOWN |
| novel_mir1128 | 0 | 117 | 7.823488223 | UP |
| novel_mir94 | 159 | 28 | -2.55240453 | DOWN |
| novel_mir120 | 11 | 112 | 3.301046807 | UP |
| novel_mir236 | 88 | 0 | -7.506308115 | DOWN |
| gga-miR-12273-5p | 358 | 156 | -1.245290055 | DOWN |
| gga-miR-148a-5p | 248 | 87 | -1.558129311 | DOWN |
| novel_mir546 | 105 | 13 | -3.060682296 | DOWN |
| novel_mir1107 | 0 | 80 | 7.275051598 | UP |
| novel_mir269 | 108 | 19 | -2.553836485 | DOWN |
| novel_mir46 | 166 | 49 | -1.807206084 | DOWN |
| novel_mir489 | 63 | 0 | -7.02415642 | DOWN |
| novel_mir569 | 245 | 105 | -1.269268918 | DOWN |
| novel_mir491 | 335 | 168 | -1.042576359 | DOWN |
| gga-miR-15c-3p | 81 | 11 | -2.927294881 | DOWN |
| novel_mir440 | 223 | 92 | -1.32421444 | DOWN |
| novel_mir480 | 15 | 88 | 2.505664527 | UP |
| novel_mir196 | 49 | 0 | -6.661586341 | DOWN |
| novel_mir692 | 49 | 0 | -6.661586341 | DOWN |
| gga-miR-132b-5p | 28 | 113 | 1.965947544 | UP |
| novel_mir763 | 87 | 18 | -2.319894991 | DOWN |
| novel_mir500 | 92 | 21 | -2.17812103 | DOWN |
| novel_mir271 | 46 | 0 | -6.570438452 | DOWN |
| novel_mir629 | 46 | 0 | -6.570438452 | DOWN |
| novel_mir237 | 44 | 0 | -6.506308115 | DOWN |
| novel_mir431 | 89 | 21 | -2.130292505 | DOWN |
| novel_mir262 | 111 | 33 | -1.796898243 | DOWN |
| gga-miR-1737 | 78 | 16 | -2.332278715 | DOWN |
| novel_mir945 | 99 | 27 | -1.921345614 | DOWN |
| novel_mir550 | 229 | 111 | -1.091664418 | DOWN |
| novel_mir820 | 42 | 0 | -6.439193919 | DOWN |
| gga-miR-1557 | 108 | 33 | -1.757369879 | DOWN |
| novel_mir52 | 139 | 52 | -1.465377851 | DOWN |
| novel_mir881 | 240 | 122 | -1.023029754 | DOWN |
| novel_mir790 | 39 | 0 | -6.332278715 | DOWN |
| novel_mir955 | 202 | 96 | -1.120125478 | DOWN |
| gga-miR-6651-5p | 8 | 59 | 2.835766553 | UP |
| novel_mir132 | 38 | 0 | -6.29480401 | DOWN |
| novel_mir276 | 38 | 0 | -6.29480401 | DOWN |
| novel_mir304 | 38 | 0 | -6.29480401 | DOWN |
| novel_mir1070 | 0 | 39 | 6.238525722 | UP |
| novel_mir220 | 118 | 41 | -1.571967541 | DOWN |
| novel_mir637 | 62 | 11 | -2.541641188 | DOWN |
| novel_mir264 | 35 | 0 | -6.176159513 | DOWN |
| novel_mir335 | 212 | 108 | -1.019909449 | DOWN |
| novel_mir609 | 34 | 0 | -6.134339338 | DOWN |
| novel_mir788 | 34 | 0 | -6.134339338 | DOWN |
| novel_mir1190 | 69 | 16 | -2.155400953 | DOWN |
| gga-miR-1788-3p | 148 | 64 | -1.256329862 | DOWN |
| novel_mir748 | 139 | 58 | -1.307836574 | DOWN |
| novel_mir1143 | 11 | 61 | 2.424429223 | UP |
| novel_mir203 | 33 | 0 | -6.091270616 | DOWN |
| novel_mir682 | 127 | 51 | -1.363135841 | DOWN |
| gga-miR-12229-5p | 181 | 88 | -1.087290765 | DOWN |
| novel_mir205 | 86 | 26 | -1.772701533 | DOWN |
| novel_mir709 | 125 | 51 | -1.340235439 | DOWN |
| novel_mir653 | 31 | 0 | -6.001072807 | DOWN |
| novel_mir735 | 77 | 22 | -1.854231418 | DOWN |
| novel_mir681 | 70 | 159 | 1.136723442 | UP |
| novel_mir1004 | 52 | 10 | -2.42538812 | DOWN |
| novel_mir286 | 29 | 0 | -5.904857492 | DOWN |
| novel_mir661 | 29 | 0 | -5.904857492 | DOWN |
| novel_mir685 | 79 | 25 | -1.706801055 | DOWN |
| novel_mir1059 | 105 | 41 | -1.403570009 | DOWN |
| novel_mir624 | 28 | 0 | -5.854231418 | DOWN |
| novel_mir412 | 78 | 25 | -1.688422526 | DOWN |
| novel_mir1018 | 60 | 15 | -2.046876496 | DOWN |
| novel_mir699 | 79 | 26 | -1.650217526 | DOWN |
| novel_mir822 | 104 | 42 | -1.354998792 | DOWN |
| novel_mir781 | 10 | 51 | 2.303620751 | UP |
| novel_mir960 | 0 | 27 | 5.708011006 | UP |
| novel_mir82 | 71 | 22 | -1.737191997 | DOWN |
| novel_mir468 | 26 | 0 | -5.747316215 | DOWN |
| novel_mir705 | 26 | 0 | -5.747316215 | DOWN |
| novel_mir146 | 81 | 28 | -1.579371577 | DOWN |
| novel_mir117 | 26 | 81 | 1.592533788 | UP |
| novel_mir1171 | 0 | 26 | 5.653563222 | UP |
| novel_mir265 | 0 | 26 | 5.653563222 | UP |
| novel_mir443 | 0 | 26 | 5.653563222 | UP |
| novel_mir784 | 25 | 0 | -5.690732686 | DOWN |
| novel_mir914 | 25 | 0 | -5.690732686 | DOWN |
| novel_mir666 | 59 | 16 | -1.929519546 | DOWN |
| novel_mir213 | 16 | 61 | 1.883860841 | UP |
| novel_mir578 | 12 | 53 | 2.096081457 | UP |
| novel_mir1047 | 24 | 0 | -5.631838997 | DOWN |
| novel_mir1098 | 24 | 0 | -5.631838997 | DOWN |
| novel_mir324 | 24 | 0 | -5.631838997 | DOWN |
| novel_mir601 | 24 | 0 | -5.631838997 | DOWN |
| novel_mir853 | 24 | 0 | -5.631838997 | DOWN |
| novel_mir1250 | 0 | 24 | 5.538086004 | UP |
| novel_mir174 | 0 | 24 | 5.538086004 | UP |
| novel_mir585 | 0 | 24 | 5.538086004 | UP |
| novel_mir883 | 0 | 24 | 5.538086004 | UP |
| novel_mir1284 | 23 | 0 | -5.570438452 | DOWN |
| novel_mir291 | 23 | 0 | -5.570438452 | DOWN |
| novel_mir391 | 23 | 0 | -5.570438452 | DOWN |
| novel_mir796 | 23 | 0 | -5.570438452 | DOWN |
| novel_mir921 | 23 | 0 | -5.570438452 | DOWN |
| novel_mir1084 | 116 | 54 | -1.149969989 | DOWN |
| novel_mir839 | 58 | 17 | -1.81739465 | DOWN |
| novel_mir1252 | 0 | 23 | 5.47668546 | UP |
| novel_mir1240 | 22 | 0 | -5.506308115 | DOWN |
| novel_mir210 | 22 | 0 | -5.506308115 | DOWN |
| novel_mir425 | 22 | 0 | -5.506308115 | DOWN |
| novel_mir779 | 22 | 0 | -5.506308115 | DOWN |
| novel_mir90 | 22 | 0 | -5.506308115 | DOWN |
| novel_mir924 | 22 | 0 | -5.506308115 | DOWN |
| novel_mir962 | 22 | 0 | -5.506308115 | DOWN |
| novel_mir1016 | 0 | 22 | 5.412555122 | UP |
| novel_mir1141 | 0 | 22 | 5.412555122 | UP |
| novel_mir140 | 0 | 22 | 5.412555122 | UP |
| novel_mir401 | 0 | 22 | 5.412555122 | UP |
| novel_mir80 | 0 | 22 | 5.412555122 | UP |
| novel_mir239 | 21 | 0 | -5.439193919 | DOWN |
| novel_mir356 | 21 | 0 | -5.439193919 | DOWN |
| novel_mir482 | 21 | 0 | -5.439193919 | DOWN |
| novel_mir765 | 21 | 0 | -5.439193919 | DOWN |
| novel_mir333 | 69 | 25 | -1.511544763 | DOWN |
| gga-miR-144-3p | 50 | 14 | -1.883377764 | DOWN |
| gga-miR-12268-5p | 46 | 12 | -1.985475952 | DOWN |
| novel_mir508 | 73 | 28 | -1.429346133 | DOWN |
| novel_mir1268 | 10 | 44 | 2.090627027 | UP |
| novel_mir1274 | 10 | 44 | 2.090627027 | UP |
| novel_mir1075 | 20 | 0 | -5.368804591 | DOWN |
| novel_mir641 | 20 | 0 | -5.368804591 | DOWN |
| novel_mir715 | 20 | 0 | -5.368804591 | DOWN |
| novel_mir867 | 20 | 0 | -5.368804591 | DOWN |
| novel_mir874 | 20 | 0 | -5.368804591 | DOWN |
| novel_mir654 | 24 | 69 | 1.47668546 | UP |
| novel_mir317 | 43 | 11 | -2.013709632 | DOWN |
| novel_mir642 | 43 | 11 | -2.013709632 | DOWN |
| novel_mir1040 | 0 | 20 | 5.275051598 | UP |
| novel_mir538 | 19 | 0 | -5.29480401 | DOWN |
| novel_mir837 | 19 | 0 | -5.29480401 | DOWN |
| gga-miR-6544-5p | 37 | 89 | 1.219403569 | UP |
| novel_mir975 | 94 | 44 | -1.142033729 | DOWN |
| novel_mir507 | 0 | 19 | 5.201051017 | UP |
| novel_mir1149 | 47 | 14 | -1.794110426 | DOWN |
| novel_mir712 | 18 | 0 | -5.216801498 | DOWN |
| gga-miR-1592 | 87 | 40 | -1.167891897 | DOWN |
| novel_mir963 | 10 | 41 | 1.988747413 | UP |
| gga-miR-2184b-3p | 30 | 76 | 1.294160421 | UP |
| novel_mir130 | 39 | 90 | 1.159574381 | UP |
| novel_mir533 | 82 | 37 | -1.194975135 | DOWN |
| novel_mir720 | 93 | 45 | -1.094182211 | DOWN |
| novel_mir1135 | 17 | 0 | -5.134339338 | DOWN |
| novel_mir450 | 17 | 0 | -5.134339338 | DOWN |
| novel_mir542 | 17 | 0 | -5.134339338 | DOWN |
| novel_mir1035 | 10 | 40 | 1.953123504 | UP |
| novel_mir1156 | 10 | 40 | 1.953123504 | UP |
| novel_mir519 | 0 | 17 | 5.040586345 | UP |
| novel_mir871 | 0 | 17 | 5.040586345 | UP |
| novel_mir158 | 41 | 12 | -1.819466 | DOWN |
| novel_mir1031 | 38 | 86 | 1.131460745 | UP |
| novel_mir1095 | 16 | 0 | -5.046876496 | DOWN |
| novel_mir1248 | 16 | 0 | -5.046876496 | DOWN |
| novel_mir133 | 16 | 0 | -5.046876496 | DOWN |
| novel_mir161 | 16 | 0 | -5.046876496 | DOWN |
| novel_mir505 | 16 | 0 | -5.046876496 | DOWN |
| novel_mir555 | 16 | 0 | -5.046876496 | DOWN |
| novel_mir861 | 16 | 0 | -5.046876496 | DOWN |
| novel_mir868 | 16 | 0 | -5.046876496 | DOWN |
| novel_mir743 | 37 | 10 | -1.934401767 | DOWN |
| novel_mir247 | 60 | 24 | -1.368804591 | DOWN |
| gga-miR-490-5p | 70 | 31 | -1.221963203 | DOWN |
| novel_mir284 | 0 | 16 | 4.953123504 | UP |
| novel_mir316 | 0 | 16 | 4.953123504 | UP |
| novel_mir626 | 0 | 16 | 4.953123504 | UP |
| novel_mir413 | 38 | 11 | -1.835372391 | DOWN |
| novel_mir1237 | 15 | 0 | -4.953767092 | DOWN |
| novel_mir564 | 15 | 0 | -4.953767092 | DOWN |
| novel_mir673 | 15 | 0 | -4.953767092 | DOWN |
| novel_mir901 | 15 | 0 | -4.953767092 | DOWN |
| novel_mir330 | 66 | 29 | -1.233289621 | DOWN |
| novel_mir595 | 11 | 39 | 1.779094104 | UP |
| gga-miR-205c-3p | 84 | 42 | -1.046876496 | DOWN |
| novel_mir454 | 0 | 15 | 4.860014099 | UP |
| novel_mir486 | 0 | 15 | 4.860014099 | UP |
| gga-miR-12223-3p | 74 | 35 | -1.127046845 | DOWN |
| novel_mir695 | 65 | 29 | -1.211263314 | DOWN |
| gga-miR-6572-3p | 1 | 16 | 3.953123504 | UP |
| novel_mir987 | 38 | 12 | -1.709841509 | DOWN |
| novel_mir1009 | 14 | 0 | -4.854231418 | DOWN |
| novel_mir1029 | 14 | 0 | -4.854231418 | DOWN |
| novel_mir1157 | 14 | 0 | -4.854231418 | DOWN |
| novel_mir1222 | 14 | 0 | -4.854231418 | DOWN |
| novel_mir460 | 14 | 0 | -4.854231418 | DOWN |
| novel_mir648 | 14 | 0 | -4.854231418 | DOWN |
| novel_mir701 | 14 | 0 | -4.854231418 | DOWN |
| novel_mir714 | 14 | 0 | -4.854231418 | DOWN |
| novel_mir718 | 14 | 0 | -4.854231418 | DOWN |
| novel_mir825 | 14 | 0 | -4.854231418 | DOWN |
| novel_mir972 | 14 | 0 | -4.854231418 | DOWN |
| gga-miR-1765 | 71 | 34 | -1.109160775 | DOWN |
| novel_mir363 | 44 | 16 | -1.506308115 | DOWN |
| gga-miR-1463 | 20 | 3 | -2.783842091 | DOWN |
| novel_mir208 | 0 | 14 | 4.760478426 | UP |
| novel_mir227 | 0 | 14 | 4.760478426 | UP |
| novel_mir856 | 0 | 14 | 4.760478426 | UP |
| novel_mir961 | 0 | 14 | 4.760478426 | UP |
| novel_mir843 | 72 | 35 | -1.087518481 | DOWN |
| novel_mir766 | 11 | 37 | 1.703145251 | UP |
| novel_mir818 | 11 | 37 | 1.703145251 | UP |
| novel_mir144 | 13 | 0 | -4.747316215 | DOWN |
| novel_mir194 | 13 | 0 | -4.747316215 | DOWN |
| novel_mir298 | 13 | 0 | -4.747316215 | DOWN |
| novel_mir439 | 13 | 0 | -4.747316215 | DOWN |
| novel_mir659 | 13 | 0 | -4.747316215 | DOWN |
| novel_mir801 | 13 | 0 | -4.747316215 | DOWN |
| novel_mir805 | 13 | 0 | -4.747316215 | DOWN |
| novel_mir842 | 13 | 0 | -4.747316215 | DOWN |
| novel_mir587 | 33 | 10 | -1.769342521 | DOWN |
| novel_mir672 | 33 | 10 | -1.769342521 | DOWN |
| novel_mir1064 | 0 | 13 | 4.653563222 | UP |
| novel_mir1068 | 0 | 13 | 4.653563222 | UP |
| novel_mir1082 | 0 | 13 | 4.653563222 | UP |
| novel_mir1083 | 0 | 13 | 4.653563222 | UP |
| novel_mir359 | 0 | 13 | 4.653563222 | UP |
| novel_mir691 | 0 | 13 | 4.653563222 | UP |
| novel_mir759 | 0 | 13 | 4.653563222 | UP |
| novel_mir873 | 0 | 13 | 4.653563222 | UP |
| gga-miR-12241-3p | 27 | 7 | -1.994409077 | DOWN |
| novel_mir151 | 29 | 65 | 1.117510321 | UP |
| novel_mir1057 | 12 | 0 | -4.631838997 | DOWN |
| novel_mir1091 | 12 | 0 | -4.631838997 | DOWN |
| novel_mir1101 | 12 | 0 | -4.631838997 | DOWN |
| novel_mir1105 | 12 | 0 | -4.631838997 | DOWN |
| novel_mir1179 | 12 | 0 | -4.631838997 | DOWN |
| novel_mir1187 | 12 | 0 | -4.631838997 | DOWN |
| novel_mir200 | 12 | 0 | -4.631838997 | DOWN |
| novel_mir202 | 12 | 0 | -4.631838997 | DOWN |
| novel_mir296 | 12 | 0 | -4.631838997 | DOWN |
| novel_mir518 | 12 | 0 | -4.631838997 | DOWN |
| novel_mir529 | 12 | 0 | -4.631838997 | DOWN |
| novel_mir576 | 12 | 0 | -4.631838997 | DOWN |
| novel_mir633 | 12 | 0 | -4.631838997 | DOWN |
| novel_mir828 | 12 | 0 | -4.631838997 | DOWN |
| novel_mir911 | 12 | 0 | -4.631838997 | DOWN |
| novel_mir936 | 12 | 0 | -4.631838997 | DOWN |
| novel_mir977 | 12 | 0 | -4.631838997 | DOWN |
| novel_mir135 | 55 | 25 | -1.18438002 | DOWN |
| gga-miR-12236-3p | 33 | 11 | -1.631838997 | DOWN |
| gga-miR-7467-3p | 33 | 11 | -1.631838997 | DOWN |
| novel_mir1079 | 33 | 11 | -1.631838997 | DOWN |
| novel_mir79 | 32 | 68 | 1.040586345 | UP |
| novel_mir1044 | 0 | 12 | 4.538086004 | UP |
| novel_mir1203 | 0 | 12 | 4.538086004 | UP |
| novel_mir282 | 0 | 12 | 4.538086004 | UP |
| novel_mir496 | 0 | 12 | 4.538086004 | UP |
| novel_mir534 | 0 | 12 | 4.538086004 | UP |
| novel_mir608 | 0 | 12 | 4.538086004 | UP |
| novel_mir621 | 0 | 12 | 4.538086004 | UP |
| novel_mir683 | 0 | 12 | 4.538086004 | UP |
| novel_mir782 | 0 | 12 | 4.538086004 | UP |
| novel_mir903 | 0 | 12 | 4.538086004 | UP |
| novel_mir950 | 0 | 12 | 4.538086004 | UP |
| novel_mir1006 | 31 | 10 | -1.679144712 | DOWN |
| novel_mir878 | 61 | 30 | -1.070723238 | DOWN |
| novel_mir446 | 15 | 41 | 1.403784913 | UP |
| novel_mir589 | 30 | 64 | 1.046232908 | UP |
| novel_mir1002 | 11 | 0 | -4.506308115 | DOWN |
| novel_mir1013 | 11 | 0 | -4.506308115 | DOWN |
| novel_mir1027 | 11 | 0 | -4.506308115 | DOWN |
| novel_mir1034 | 11 | 0 | -4.506308115 | DOWN |
| novel_mir1041 | 11 | 0 | -4.506308115 | DOWN |
| novel_mir1103 | 11 | 0 | -4.506308115 | DOWN |
| novel_mir1163 | 11 | 0 | -4.506308115 | DOWN |
| novel_mir1180 | 11 | 0 | -4.506308115 | DOWN |
| novel_mir1253 | 11 | 0 | -4.506308115 | DOWN |
| novel_mir1278 | 11 | 0 | -4.506308115 | DOWN |
| novel_mir139 | 11 | 0 | -4.506308115 | DOWN |
| novel_mir179 | 11 | 0 | -4.506308115 | DOWN |
| novel_mir183 | 11 | 0 | -4.506308115 | DOWN |
| novel_mir197 | 11 | 0 | -4.506308115 | DOWN |
| novel_mir201 | 11 | 0 | -4.506308115 | DOWN |
| novel_mir280 | 11 | 0 | -4.506308115 | DOWN |
| novel_mir322 | 11 | 0 | -4.506308115 | DOWN |
| novel_mir361 | 11 | 0 | -4.506308115 | DOWN |
| novel_mir447 | 11 | 0 | -4.506308115 | DOWN |
| novel_mir485 | 11 | 0 | -4.506308115 | DOWN |
| novel_mir494 | 11 | 0 | -4.506308115 | DOWN |
| novel_mir495 | 11 | 0 | -4.506308115 | DOWN |
| novel_mir506 | 11 | 0 | -4.506308115 | DOWN |
| novel_mir645 | 11 | 0 | -4.506308115 | DOWN |
| novel_mir668 | 11 | 0 | -4.506308115 | DOWN |
| novel_mir758 | 11 | 0 | -4.506308115 | DOWN |
| novel_mir794 | 11 | 0 | -4.506308115 | DOWN |
| novel_mir880 | 11 | 0 | -4.506308115 | DOWN |
| novel_mir959 | 11 | 0 | -4.506308115 | DOWN |
| novel_mir982 | 11 | 0 | -4.506308115 | DOWN |
