# Supplementary material for: Interactions Among Expressed MicroRNAs and mRNAs in the Early Stages of Fowl Adenovirus Aerotype 4-Infected Leghorn Male Hepatocellular Cells
Source: Front Microbiol. 2020 May 19;11:831. doi: 10.3389/fmicb.2020.00831 (PMC7248314; doi:10.3389/fmicb.2020.00831)
Supplement: FIGURE S1 — Hierarchical clustering analysis of 784 differentially expressed miRNAs at three time points. The color in the heat map represents the gene expression changes. Red indicates the upregulation of gene expression, blue indicates the downregulation of expression, the darker color indicates a notable degree of differential gene expression, and white indicates no activity. [file Data_Sheet_1.ZIP › supplementary files/Table S3.doc]

TABLE S3 Differentially expressed miRNAs between mock and infected LMHs at 120 min

| **miRNA id** | **Read count**  **(Mock)** | **Read count**  **(FAdV4_120)** | **log2Ratio**  **(FAdV4_120/Mock)** | **Up down regulation** |
| --- | --- | --- | --- | --- |
| gga-miR-148a-3p | 251503 | 111958 | -1.063094829 | DOWN |
| gga-miR-1674 | 17196 | 7859 | -1.025132198 | DOWN |
| novel_mir1223 | 4061 | 20475 | 2.43847968 | UP |
| novel_mir261 | 5956 | 42486 | 2.939095023 | UP |
| novel_mir935 | 22 | 3319 | 7.341624456 | UP |
| novel_mir909 | 171 | 1547 | 3.281928127 | UP |
| gga-miR-7475-5p | 49 | 757 | 4.053962806 | UP |
| gga-miR-34c-5p | 647 | 18 | -5.06317374 | DOWN |
| novel_mir20 | 845 | 2092 | 1.412382765 | UP |
| novel_mir480 | 15 | 249 | 4.157634497 | UP |
| gga-miR-128-2-5p | 1147 | 498 | -1.099124584 | DOWN |
| gga-miR-3538 | 298 | 719 | 1.3752026 | UP |
| novel_mir1209 | 13 | 198 | 4.033440062 | UP |
| novel_mir1107 | 0 | 203 | 8.769859077 | UP |
| gga-miR-1434 | 463 | 909 | 1.077791261 | UP |
| novel_mir395 | 0 | 185 | 8.635904621 | UP |
| novel_mir491 | 335 | 664 | 1.091545306 | UP |
| novel_mir1128 | 0 | 124 | 8.05871947 | UP |
| gga-miR-100-3p | 673 | 308 | -1.023152994 | DOWN |
| novel_mir509 | 432 | 158 | -1.346583594 | DOWN |
| novel_mir683 | 0 | 111 | 7.898939026 | UP |
| novel_mir326 | 583 | 257 | -1.077204364 | DOWN |
| gga-miR-12245-3p | 43 | 187 | 2.225152865 | UP |
| gga-miR-6651-5p | 8 | 93 | 3.643681971 | UP |
| novel_mir1154 | 0 | 79 | 7.408303908 | UP |
| gga-miR-301b-5p | 290 | 106 | -1.347465475 | DOWN |
| novel_mir120 | 11 | 88 | 3.10452316 | UP |
| gga-miR-96-3p | 12 | 90 | 3.011413756 | UP |
| novel_mir333 | 69 | 0 | -7.004001297 | DOWN |
| gga-miR-148a-5p | 248 | 96 | -1.26471065 | DOWN |
| novel_mir146 | 81 | 10 | -2.913398748 | DOWN |
| novel_mir770 | 52 | 0 | -6.595916558 | DOWN |
| novel_mir898 | 47 | 0 | -6.450065692 | DOWN |
| novel_mir642 | 43 | 0 | -6.321741595 | DOWN |
| novel_mir430 | 0 | 37 | 6.313976526 | UP |
| novel_mir790 | 39 | 0 | -6.180879059 | DOWN |
| novel_mir512 | 78 | 16 | -2.180879059 | DOWN |
| novel_mir166 | 0 | 36 | 6.274448162 | UP |
| novel_mir413 | 38 | 0 | -6.143404353 | DOWN |
| novel_mir544 | 38 | 0 | -6.143404353 | DOWN |
| novel_mir167 | 0 | 35 | 6.233806177 | UP |
| novel_mir743 | 37 | 0 | -6.104930206 | DOWN |
| novel_mir264 | 35 | 0 | -6.024759857 | DOWN |
| novel_mir932 | 35 | 0 | -6.024759857 | DOWN |
| novel_mir764 | 34 | 0 | -5.982939681 | DOWN |
| novel_mir344 | 144 | 55 | -1.284042128 | DOWN |
| novel_mir1263 | 33 | 0 | -5.939870959 | DOWN |
| novel_mir154 | 58 | 10 | -2.43152974 | DOWN |
| novel_mir623 | 59 | 11 | -2.318688271 | DOWN |
| novel_mir934 | 0 | 29 | 5.962504155 | UP |
| novel_mir704 | 11 | 52 | 2.34553126 | UP |
| novel_mir959 | 11 | 52 | 2.34553126 | UP |
| novel_mir454 | 0 | 28 | 5.911878082 | UP |
| novel_mir846 | 0 | 28 | 5.911878082 | UP |
| novel_mir427 | 30 | 0 | -5.802367436 | DOWN |
| novel_mir1117 | 76 | 20 | -1.821476258 | DOWN |
| novel_mir1115 | 10 | 48 | 2.367557566 | UP |
| novel_mir1081 | 28 | 0 | -5.702831762 | DOWN |
| novel_mir624 | 28 | 0 | -5.702831762 | DOWN |
| novel_mir682 | 127 | 51 | -1.211736185 | DOWN |
| novel_mir198 | 27 | 0 | -5.650364342 | DOWN |
| novel_mir1073 | 0 | 25 | 5.74837935 | UP |
| novel_mir664 | 0 | 25 | 5.74837935 | UP |
| novel_mir784 | 25 | 73 | 1.650491529 | UP |
| novel_mir948 | 26 | 0 | -5.595916558 | DOWN |
| novel_mir135 | 55 | 12 | -2.091874053 | DOWN |
| novel_mir140 | 0 | 24 | 5.689485661 | UP |
| novel_mir488 | 80 | 25 | -1.573548745 | DOWN |
| novel_mir212 | 78 | 24 | -1.595916558 | DOWN |
| novel_mir1152 | 0 | 23 | 5.628085116 | UP |
| novel_mir1227 | 0 | 23 | 5.628085116 | UP |
| novel_mir80 | 0 | 23 | 5.628085116 | UP |
| novel_mir1256 | 24 | 0 | -5.480439341 | DOWN |
| novel_mir188 | 24 | 0 | -5.480439341 | DOWN |
| novel_mir229 | 24 | 0 | -5.480439341 | DOWN |
| novel_mir601 | 24 | 0 | -5.480439341 | DOWN |
| novel_mir853 | 24 | 0 | -5.480439341 | DOWN |
| novel_mir883 | 0 | 22 | 5.563954779 | UP |
| novel_mir1004 | 52 | 12 | -2.010954057 | DOWN |
| novel_mir681 | 70 | 132 | 1.019634263 | UP |
| novel_mir391 | 23 | 0 | -5.419038796 | DOWN |
| novel_mir470 | 23 | 0 | -5.419038796 | DOWN |
| novel_mir655 | 23 | 0 | -5.419038796 | DOWN |
| novel_mir72 | 23 | 0 | -5.419038796 | DOWN |
| novel_mir796 | 23 | 0 | -5.419038796 | DOWN |
| novel_mir636 | 140 | 64 | -1.024759857 | DOWN |
| novel_mir1045 | 0 | 21 | 5.496840583 | UP |
| novel_mir691 | 0 | 21 | 5.496840583 | UP |
| novel_mir1262 | 57 | 15 | -1.821476258 | DOWN |
| gga-miR-1563 | 53 | 108 | 1.131490208 | UP |
| novel_mir1240 | 22 | 0 | -5.354908459 | DOWN |
| novel_mir128 | 22 | 0 | -5.354908459 | DOWN |
| novel_mir425 | 22 | 0 | -5.354908459 | DOWN |
| novel_mir475 | 22 | 0 | -5.354908459 | DOWN |
| novel_mir90 | 22 | 0 | -5.354908459 | DOWN |
| novel_mir924 | 22 | 0 | -5.354908459 | DOWN |
| novel_mir142 | 46 | 10 | -2.097110701 | DOWN |
| novel_mir156 | 46 | 10 | -2.097110701 | DOWN |
| novel_mir247 | 60 | 17 | -1.714904594 | DOWN |
| novel_mir414 | 85 | 31 | -1.350671466 | DOWN |
| novel_mir1143 | 11 | 43 | 2.071356296 | UP |
| novel_mir316 | 0 | 20 | 5.426451255 | UP |
| novel_mir843 | 72 | 24 | -1.480439341 | DOWN |
| novel_mir1148 | 21 | 0 | -5.287794263 | DOWN |
| novel_mir356 | 21 | 0 | -5.287794263 | DOWN |
| novel_mir765 | 21 | 0 | -5.287794263 | DOWN |
| novel_mir131 | 45 | 10 | -2.065401841 | DOWN |
| novel_mir456 | 55 | 15 | -1.769945958 | DOWN |
| novel_mir500 | 92 | 36 | -1.249113795 | DOWN |
| novel_mir197 | 11 | 42 | 2.037408964 | UP |
| novel_mir79 | 32 | 75 | 1.333341851 | UP |
| novel_mir117 | 26 | 66 | 1.448477561 | UP |
| novel_mir570 | 44 | 10 | -2.032980364 | DOWN |
| novel_mir1075 | 20 | 0 | -5.217404935 | DOWN |
| novel_mir715 | 20 | 0 | -5.217404935 | DOWN |
| novel_mir874 | 20 | 0 | -5.217404935 | DOWN |
| novel_mir199 | 38 | 83 | 1.231635078 | UP |
| novel_mir226 | 99 | 42 | -1.132516037 | DOWN |
| gga-miR-181b-2-3p | 63 | 21 | -1.480439341 | DOWN |
| novel_mir411 | 13 | 43 | 1.830348197 | UP |
| gga-miR-15c-3p | 81 | 32 | -1.235326843 | DOWN |
| gga-miR-383-5p | 20 | 54 | 1.537482567 | UP |
| gga-miR-6544-5p | 37 | 79 | 1.198850543 | UP |
| novel_mir712 | 18 | 0 | -5.065401841 | DOWN |
| novel_mir735 | 77 | 30 | -1.255372785 | DOWN |
| novel_mir847 | 47 | 13 | -1.749625973 | DOWN |
| novel_mir667 | 43 | 11 | -1.862309976 | DOWN |
| novel_mir244 | 0 | 16 | 5.10452316 | UP |
| novel_mir687 | 0 | 16 | 5.10452316 | UP |
| novel_mir903 | 0 | 16 | 5.10452316 | UP |
| novel_mir1135 | 17 | 0 | -4.982939681 | DOWN |
| novel_mir450 | 17 | 0 | -4.982939681 | DOWN |
| novel_mir542 | 17 | 0 | -4.982939681 | DOWN |
| novel_mir990 | 17 | 0 | -4.982939681 | DOWN |
| novel_mir627 | 46 | 13 | -1.718599078 | DOWN |
| novel_mir1018 | 60 | 21 | -1.410050013 | DOWN |
| novel_mir1205 | 12 | 39 | 1.804962878 | UP |
| novel_mir209 | 73 | 29 | -1.227320404 | DOWN |
| novel_mir1044 | 0 | 15 | 5.011413756 | UP |
| novel_mir815 | 0 | 15 | 5.011413756 | UP |
| novel_mir819 | 0 | 15 | 5.011413756 | UP |
| novel_mir978 | 0 | 15 | 5.011413756 | UP |
| novel_mir158 | 41 | 11 | -1.793597226 | DOWN |
| novel_mir119 | 41 | 81 | 1.086821158 | UP |
| novel_mir1095 | 16 | 0 | -4.89547684 | DOWN |
| novel_mir1248 | 16 | 0 | -4.89547684 | DOWN |
| novel_mir133 | 16 | 0 | -4.89547684 | DOWN |
| novel_mir505 | 16 | 0 | -4.89547684 | DOWN |
| novel_mir555 | 16 | 0 | -4.89547684 | DOWN |
| gga-miR-6570-5p | 23 | 3 | -2.834076295 | DOWN |
| gga-miR-1b-5p | 23 | 55 | 1.362320918 | UP |
| novel_mir137 | 40 | 11 | -1.757973316 | DOWN |
| novel_mir424 | 40 | 11 | -1.757973316 | DOWN |
| novel_mir196 | 49 | 16 | -1.510186684 | DOWN |
| novel_mir1113 | 0 | 14 | 4.911878082 | UP |
| novel_mir1213 | 0 | 14 | 4.911878082 | UP |
| novel_mir1232 | 0 | 14 | 4.911878082 | UP |
| novel_mir321 | 0 | 14 | 4.911878082 | UP |
| novel_mir1124 | 15 | 0 | -4.802367436 | DOWN |
| novel_mir1237 | 15 | 0 | -4.802367436 | DOWN |
| novel_mir564 | 15 | 0 | -4.802367436 | DOWN |
| novel_mir673 | 15 | 0 | -4.802367436 | DOWN |
| novel_mir991 | 11 | 35 | 1.774374558 | UP |
| novel_mir458 | 57 | 21 | -1.336049431 | DOWN |
| novel_mir1037 | 12 | 36 | 1.689485661 | UP |
| novel_mir1014 | 0 | 13 | 4.804962878 | UP |
| novel_mir251 | 0 | 13 | 4.804962878 | UP |
| novel_mir364 | 0 | 13 | 4.804962878 | UP |
| novel_mir386 | 0 | 13 | 4.804962878 | UP |
| novel_mir675 | 0 | 13 | 4.804962878 | UP |
| novel_mir980 | 0 | 13 | 4.804962878 | UP |
| novel_mir1009 | 14 | 0 | -4.702831762 | DOWN |
| novel_mir1051 | 14 | 0 | -4.702831762 | DOWN |
| novel_mir400 | 14 | 0 | -4.702831762 | DOWN |
| novel_mir523 | 14 | 0 | -4.702831762 | DOWN |
| novel_mir583 | 14 | 0 | -4.702831762 | DOWN |
| novel_mir648 | 14 | 0 | -4.702831762 | DOWN |
| novel_mir701 | 14 | 0 | -4.702831762 | DOWN |
| novel_mir714 | 14 | 0 | -4.702831762 | DOWN |
| novel_mir718 | 14 | 0 | -4.702831762 | DOWN |
| novel_mir825 | 14 | 0 | -4.702831762 | DOWN |
| novel_mir419 | 29 | 61 | 1.177279503 | UP |
| novel_mir707 | 11 | 34 | 1.732554383 | UP |
| novel_mir276 | 38 | 11 | -1.683972735 | DOWN |
| novel_mir304 | 38 | 11 | -1.683972735 | DOWN |
| novel_mir141 | 57 | 22 | -1.268935235 | DOWN |
| novel_mir699 | 79 | 36 | -1.029332587 | DOWN |
| novel_mir1021 | 0 | 12 | 4.689485661 | UP |
| novel_mir1066 | 0 | 12 | 4.689485661 | UP |
| novel_mir1170 | 0 | 12 | 4.689485661 | UP |
| novel_mir1171 | 0 | 12 | 4.689485661 | UP |
| novel_mir1207 | 0 | 12 | 4.689485661 | UP |
| novel_mir174 | 0 | 12 | 4.689485661 | UP |
| novel_mir253 | 0 | 12 | 4.689485661 | UP |
| novel_mir289 | 0 | 12 | 4.689485661 | UP |
| novel_mir420 | 0 | 12 | 4.689485661 | UP |
| novel_mir711 | 0 | 12 | 4.689485661 | UP |
| novel_mir776 | 0 | 12 | 4.689485661 | UP |
| novel_mir782 | 0 | 12 | 4.689485661 | UP |
| novel_mir785 | 0 | 12 | 4.689485661 | UP |
| novel_mir918 | 0 | 12 | 4.689485661 | UP |
| novel_mir439 | 13 | 0 | -4.595916558 | DOWN |
| novel_mir659 | 13 | 0 | -4.595916558 | DOWN |
| novel_mir801 | 13 | 0 | -4.595916558 | DOWN |
| novel_mir805 | 13 | 0 | -4.595916558 | DOWN |
| novel_mir838 | 13 | 0 | -4.595916558 | DOWN |
| novel_mir842 | 13 | 0 | -4.595916558 | DOWN |
| gga-miR-1463 | 20 | 3 | -2.632442434 | DOWN |
| gga-miR-6654-3p | 20 | 3 | -2.632442434 | DOWN |
| gga-miR-6590-5p | 31 | 62 | 1.10452316 | UP |
| novel_mir272 | 45 | 16 | -1.387329936 | DOWN |
| novel_mir788 | 34 | 10 | -1.661011586 | DOWN |
| novel_mir1057 | 12 | 0 | -4.480439341 | DOWN |
| novel_mir107 | 12 | 0 | -4.480439341 | DOWN |
| novel_mir1091 | 12 | 0 | -4.480439341 | DOWN |
| novel_mir1101 | 12 | 0 | -4.480439341 | DOWN |
| novel_mir1102 | 12 | 0 | -4.480439341 | DOWN |
| novel_mir1105 | 12 | 0 | -4.480439341 | DOWN |
| novel_mir1187 | 12 | 0 | -4.480439341 | DOWN |
| novel_mir170 | 12 | 0 | -4.480439341 | DOWN |
| novel_mir177 | 12 | 0 | -4.480439341 | DOWN |
| novel_mir200 | 12 | 0 | -4.480439341 | DOWN |
| novel_mir202 | 12 | 0 | -4.480439341 | DOWN |
| novel_mir296 | 12 | 0 | -4.480439341 | DOWN |
| novel_mir303 | 12 | 0 | -4.480439341 | DOWN |
| novel_mir518 | 12 | 0 | -4.480439341 | DOWN |
| novel_mir527 | 12 | 0 | -4.480439341 | DOWN |
| novel_mir633 | 12 | 0 | -4.480439341 | DOWN |
| novel_mir792 | 12 | 0 | -4.480439341 | DOWN |
| novel_mir828 | 12 | 0 | -4.480439341 | DOWN |
| novel_mir933 | 12 | 0 | -4.480439341 | DOWN |
| novel_mir936 | 12 | 0 | -4.480439341 | DOWN |
| novel_mir977 | 12 | 0 | -4.480439341 | DOWN |
| novel_mir1008 | 0 | 11 | 4.563954779 | UP |
| novel_mir1040 | 0 | 11 | 4.563954779 | UP |
| novel_mir1134 | 0 | 11 | 4.563954779 | UP |
| novel_mir1167 | 0 | 11 | 4.563954779 | UP |
| novel_mir1182 | 0 | 11 | 4.563954779 | UP |
| novel_mir1217 | 0 | 11 | 4.563954779 | UP |
| novel_mir1250 | 0 | 11 | 4.563954779 | UP |
| novel_mir282 | 0 | 11 | 4.563954779 | UP |
| novel_mir759 | 0 | 11 | 4.563954779 | UP |
