# Supplementary material for: Interactions Among Expressed MicroRNAs and mRNAs in the Early Stages of Fowl Adenovirus Aerotype 4-Infected Leghorn Male Hepatocellular Cells
Source: Front Microbiol. 2020 May 19;11:831. doi: 10.3389/fmicb.2020.00831 (PMC7248314; doi:10.3389/fmicb.2020.00831)
Supplement: FIGURE S1 — Hierarchical clustering analysis of 784 differentially expressed miRNAs at three time points. The color in the heat map represents the gene expression changes. Red indicates the upregulation of gene expression, blue indicates the downregulation of expression, the darker color indicates a notable degree of differential gene expression, and white indicates no activity. [file Data_Sheet_1.ZIP › supplementary files/Table S5.doc]

TABLE S5 Differentially expressed mRNAs between mock and infected LMHs at 60 min

| **gene id** | **Average fpkm**  **(Mock)** | **Average fpkm**  **(FAdV4_30)** | **log2Ratio**  **(FAdV4_30/Mock)** | **Up down regulation** |
| --- | --- | --- | --- | --- |
| BGI_novel_G000918 | 0 | 1.61 | 9.36 | up |
| BGI_novel_G000681 | 0 | 0.63 | 7.73 | up |
| 100857910 | 0 | 0.93 | 7.00 | up |
| BGI_novel_G000767 | 0 | 0.54 | 6.42 | up |
| 107056385 | 0 | 0.40 | 6.26 | up |
| 107055887 | 0 | 0.38 | 5.70 | up |
| 422950 | 0.07 | 3.59 | 5.62 | up |
| 107049080 | 0.00 | 1.09 | 5.54 | up |
| 419933 | 0.01 | 0.27 | 5.46 | up |
| BGI_novel_G000967 | 0 | 0.43 | 5.34 | up |
| BGI_novel_G000667 | 0 | 1.66 | 5.27 | up |
| 100858035 | 0 | 0.28 | 5.22 | up |
| BGI_novel_G000834 | 0 | 0.22 | 5.11 | up |
| 103214197 | 0 | 0.21 | 4.95 | up |
| 417553 | 0 | 0.19 | 4.84 | up |
| 395335 | 2.17 | 50.74 | 4.54 | up |
| 107056190 | 0.06 | 1.25 | 4.38 | up |
| 424562 | 0.00 | 0.07 | 4.12 | up |
| 424409 | 5.24 | 89.73 | 4.10 | up |
| 396216 | 0.19 | 3.22 | 4.06 | up |
| 107055390 | 4.70 | 77.96 | 4.05 | up |
| 107049603 | 0.56 | 7.27 | 3.69 | up |
| 102725543 | 0.83 | 10.43 | 3.64 | up |
| 420896 | 1.29 | 12.29 | 3.56 | up |
| 107050017 | 2.18 | 25.26 | 3.53 | up |
| 107054209 | 0.01 | 0.11 | 3.52 | up |
| 420486 | 0.01 | 0.09 | 3.40 | up |
| 396384 | 1.37 | 13.81 | 3.32 | up |
| 395299 | 4.54 | 44.63 | 3.29 | up |
| 395190 | 0.03 | 0.24 | 3.28 | up |
| 100857380 | 1.69 | 15.70 | 3.21 | up |
| 107055630 | 0.02 | 0.19 | 3.17 | up |
| 395594 | 0.24 | 2.20 | 3.16 | up |
| 107054668 | 0.30 | 2.60 | 3.11 | up |
| 373931 | 62.63 | 538.67 | 3.10 | up |
| 420479 | 1.23 | 10.33 | 3.09 | up |
| 419364 | 15.71 | 127.88 | 3.02 | up |
| 423890 | 6.52 | 52.32 | 3.00 | up |
| 418479 | 1.76 | 14.06 | 3.00 | up |
| BGI_novel_G000959 | 0.12 | 0.89 | 2.97 | up |
| 420463 | 9.37 | 72.48 | 2.95 | up |
| 107056144 | 0.05 | 0.39 | 2.94 | up |
| 101750341 | 0.05 | 0.35 | 2.91 | up |
| 395872 | 0.45 | 3.34 | 2.88 | up |
| 396512 | 24.20 | 169.02 | 2.80 | up |
| 428386 | 25.75 | 173.14 | 2.75 | up |
| 395210 | 1.33 | 8.89 | 2.74 | up |
| BGI_novel_G000250 | 0.91 | 6.04 | 2.73 | up |
| 100857983 | 0.03 | 0.18 | 2.71 | up |
| BGI_novel_G000078 | 0.08 | 0.50 | 2.69 | up |
| 396495 | 0.57 | 3.64 | 2.68 | up |
| 420996 | 6.34 | 36.97 | 2.58 | up |
| 418247 | 14.66 | 89.76 | 2.55 | up |
| 107056355 | 0.15 | 0.86 | 2.54 | up |
| 424320 | 1.71 | 9.64 | 2.49 | up |
| 101749459 | 2.26 | 12.34 | 2.46 | up |
| 418741 | 0.06 | 0.29 | 2.44 | up |
| BGI_novel_G000177 | 0.91 | 4.95 | 2.44 | up |
| 107057304 | 0.21 | 1.18 | 2.42 | up |
| 107051858 | 0.14 | 0.77 | 2.40 | up |
| 395973 | 0.14 | 0.73 | 2.38 | up |
| 395663 | 1.84 | 7.01 | 2.33 | up |
| 421416 | 7.59 | 38.33 | 2.33 | up |
| 107057513 | 0.18 | 0.87 | 2.31 | up |
| 419932 | 23.42 | 114.95 | 2.29 | up |
| 101749400 | 0.35 | 1.69 | 2.29 | up |
| 101751828 | 42.99 | 199.08 | 2.21 | up |
| BGI_novel_G000472 | 0.37 | 1.68 | 2.21 | up |
| 424883 | 74.72 | 336.45 | 2.17 | up |
| 432368 | 0.36 | 1.59 | 2.16 | up |
| 428070 | 0.05 | 0.20 | 2.13 | up |
| 378903 | 14.01 | 65.54 | 2.11 | up |
| 100857932 | 5.12 | 21.99 | 2.10 | up |
| 396385 | 54.89 | 231.33 | 2.07 | up |
| 421600 | 3.44 | 14.18 | 2.06 | up |
| 427107 | 4.97 | 19.96 | 2.00 | up |
| 427805 | 0.06 | 0.24 | 1.98 | up |
| 429089 | 3.58 | 14.11 | 1.97 | up |
| 426820 | 0.17 | 0.61 | 1.97 | up |
| 395354 | 21.13 | 82.23 | 1.96 | up |
| 429152 | 0.31 | 1.21 | 1.95 | up |
| BGI_novel_G000088 | 0.07 | 0.26 | 1.95 | up |
| 395219 | 6.86 | 26.69 | 1.93 | up |
| 408036 | 2.52 | 9.68 | 1.93 | up |
| 431604 | 0.04 | 0.15 | 1.91 | up |
| 424912 | 7.22 | 27.33 | 1.91 | up |
| 417561 | 0.20 | 0.77 | 1.88 | up |
| 417960 | 3.31 | 11.80 | 1.85 | up |
| 396119 | 6.81 | 23.96 | 1.83 | up |
| 107056753 | 0.88 | 3.13 | 1.82 | up |
| 100379662 | 0.44 | 1.54 | 1.79 | up |
| 107055895 | 1.58 | 5.57 | 1.78 | up |
| 395939 | 29.09 | 100.09 | 1.78 | up |
| 768811 | 0.06 | 0.18 | 1.76 | up |
| 424312 | 14.70 | 49.73 | 1.76 | up |
| 418963 | 3.31 | 11.07 | 1.73 | up |
| 428752 | 9.25 | 30.87 | 1.73 | up |
| BGI_novel_G000182 | 0.55 | 1.79 | 1.72 | up |
| 107052968 | 0.36 | 1.18 | 1.71 | up |
| 423088 | 53.67 | 174.73 | 1.69 | up |
| 107052805 | 0.77 | 2.51 | 1.69 | up |
| 425270 | 0.14 | 0.41 | 1.69 | up |
| 417411 | 0.05 | 0.18 | 1.68 | up |
| BGI_novel_G000275 | 0.06 | 0.20 | 1.67 | up |
| 416630 | 1.86 | 5.95 | 1.67 | up |
| 771550 | 6.05 | 19.21 | 1.67 | up |
| 378914 | 2.09 | 6.64 | 1.66 | up |
| 107049468 | 5.89 | 18.61 | 1.65 | up |
| 768821 | 0.12 | 0.37 | 1.59 | up |
| 101748744 | 0.13 | 0.35 | 1.59 | up |
| BGI_novel_G000362 | 0.20 | 0.62 | 1.58 | up |
| 768536 | 2.96 | 8.87 | 1.58 | up |
| 100858838 | 8.18 | 24.33 | 1.57 | up |
| 378919 | 8.58 | 25.16 | 1.56 | up |
| 396185 | 48.16 | 140.49 | 1.54 | up |
| 417567 | 0.67 | 1.94 | 1.54 | up |
| 396481 | 4.23 | 12.18 | 1.53 | up |
| BGI_novel_G000788 | 0.70 | 1.99 | 1.52 | up |
| 378912 | 13.37 | 38.31 | 1.51 | up |
| 101750986 | 49.29 | 140.86 | 1.51 | up |
| 422106 | 0.72 | 2.08 | 1.51 | up |
| 419753 | 3.72 | 10.45 | 1.50 | up |
| 417807 | 42.81 | 121.56 | 1.50 | up |
| 768353 | 0.58 | 1.63 | 1.49 | up |
| 374059 | 9.48 | 26.19 | 1.46 | up |
| 419618 | 5.62 | 15.07 | 1.42 | up |
| 420854 | 1.55 | 4.12 | 1.41 | up |
| 418404 | 7.30 | 19.20 | 1.40 | up |
| 100858060 | 22.39 | 58.06 | 1.38 | up |
| 107052930 | 23.49 | 61.30 | 1.38 | up |
| 396451 | 0.90 | 2.39 | 1.38 | up |
| 421333 | 5.84 | 14.94 | 1.37 | up |
| 374135 | 0.74 | 1.91 | 1.36 | up |
| 100859777 | 1.51 | 3.83 | 1.35 | up |
| 107052623 | 0.86 | 2.17 | 1.34 | up |
| BGI_novel_G000866 | 0.95 | 2.36 | 1.33 | up |
| 107049140 | 6.48 | 15.36 | 1.32 | up |
| 422487 | 0.73 | 1.81 | 1.30 | up |
| 420097 | 0.22 | 0.54 | 1.29 | up |
| 107057253 | 0.61 | 1.47 | 1.28 | up |
| 378803 | 2.37 | 5.81 | 1.28 | up |
| 421276 | 17.23 | 41.27 | 1.26 | up |
| 768655 | 49.14 | 116.96 | 1.25 | up |
| BGI_novel_G000575 | 1.64 | 3.91 | 1.24 | up |
| 430666 | 0.11 | 0.25 | 1.24 | up |
| 107050448 | 0.78 | 1.95 | 1.23 | up |
| 107055314 | 1.29 | 3.01 | 1.23 | up |
| 100859283 | 0.79 | 1.82 | 1.20 | up |
| 396339 | 46.74 | 107.80 | 1.20 | up |
| 101751930 | 0.26 | 0.60 | 1.20 | up |
| 423225 | 0.23 | 0.52 | 1.19 | up |
| BGI_novel_G000130 | 0.47 | 1.08 | 1.19 | up |
| BGI_novel_G000108 | 0.31 | 0.72 | 1.18 | up |
| 419411 | 0.34 | 0.78 | 1.18 | up |
| 426211 | 5.30 | 11.83 | 1.18 | up |
| 395326 | 8.33 | 18.76 | 1.17 | up |
| 100113359 | 2.15 | 3.93 | 1.17 | up |
| 428906 | 3.58 | 7.96 | 1.17 | up |
| 417636 | 10.70 | 18.81 | 1.17 | up |
| 101749609 | 0.37 | 0.80 | 1.15 | up |
| 396393 | 1.00 | 2.23 | 1.15 | up |
| 428445 | 6.72 | 13.97 | 1.13 | up |
| 101750511 | 0.74 | 1.62 | 1.13 | up |
| 428930 | 1.05 | 2.28 | 1.12 | up |
| 395559 | 1.94 | 4.36 | 1.12 | up |
| BGI_novel_G000517 | 0.27 | 0.58 | 1.12 | up |
| 418818 | 17.03 | 37.13 | 1.12 | up |
| 396043 | 4.54 | 9.87 | 1.12 | up |
| 423079 | 1.26 | 2.86 | 1.11 | up |
| 423931 | 17.22 | 36.94 | 1.11 | up |
| 395483 | 0.71 | 1.53 | 1.10 | up |
| 396489 | 20.16 | 42.55 | 1.08 | up |
| 107050064 | 7.20 | 15.70 | 1.08 | up |
| 416507 | 50.58 | 107.05 | 1.08 | up |
| 428897 | 1.45 | 3.07 | 1.08 | up |
| 107052763 | 11.08 | 23.37 | 1.07 | up |
| 421369 | 11.55 | 24.15 | 1.06 | up |
| 100538348 | 36.29 | 49.42 | 1.06 | up |
| 417230 | 12.57 | 26.24 | 1.06 | up |
| 101750587 | 0.85 | 1.78 | 1.05 | up |
| 415686 | 0.35 | 0.73 | 1.05 | up |
| BGI_novel_G000008 | 4.41 | 9.20 | 1.05 | up |
| 775985 | 6.85 | 14.33 | 1.05 | up |
| BGI_novel_G000685 | 0.42 | 0.87 | 1.05 | up |
| 395583 | 9.62 | 19.09 | 1.04 | up |
| 422230 | 2.28 | 4.67 | 1.03 | up |
| 414893 | 0.08 | 0.15 | 1.03 | up |
| 101751870 | 0.72 | 1.48 | 1.03 | up |
| 420673 | 4.74 | 9.65 | 1.02 | up |
| 395837 | 4.05 | 8.16 | 1.01 | up |
| 428642 | 0.64 | 0.32 | -1.02 | down |
| 101747455 | 0.98 | 0.47 | -1.06 | down |
| 420370 | 1.78 | 0.86 | -1.09 | down |
| BGI_novel_G000116 | 0.35 | 0.16 | -1.09 | down |
| 395133 | 9.51 | 4.42 | -1.12 | down |
| BGI_novel_G000339 | 0.62 | 0.27 | -1.18 | down |
| 107057014 | 1.63 | 0.64 | -1.18 | down |
| 419029 | 2.28 | 1.00 | -1.20 | down |
| BGI_novel_G000156 | 2.15 | 0.94 | -1.22 | down |
| 426897 | 0.42 | 0.18 | -1.22 | down |
| 426754 | 0.76 | 0.33 | -1.22 | down |
| 107057143 | 17.78 | 7.30 | -1.28 | down |
| 395465 | 18.82 | 7.77 | -1.28 | down |
| BGI_novel_G000787 | 2.32 | 0.94 | -1.28 | down |
| 107055989 | 2.19 | 0.89 | -1.30 | down |
| 107056507 | 19.06 | 7.74 | -1.31 | down |
| BGI_novel_G000220 | 0.39 | 0.15 | -1.32 | down |
| 415756 | 2.54 | 1.03 | -1.33 | down |
| 424555 | 0.50 | 0.19 | -1.34 | down |
| 425623 | 0.62 | 0.24 | -1.37 | down |
| 420841 | 4.40 | 1.70 | -1.37 | down |
| BGI_novel_G000865 | 2.43 | 0.92 | -1.40 | down |
| 428413 | 64.80 | 24.31 | -1.43 | down |
| BGI_novel_G000102 | 0.69 | 0.26 | -1.43 | down |
| 101750988 | 16.50 | 6.35 | -1.43 | down |
| BGI_novel_G000764 | 2.81 | 1.05 | -1.46 | down |
| 100857744 | 0.34 | 0.12 | -1.46 | down |
| 107052854 | 1.97 | 0.70 | -1.49 | down |
| 107056386 | 0.83 | 0.23 | -1.52 | down |
| BGI_novel_G000216 | 0.52 | 0.18 | -1.52 | down |
| 107057082 | 0.62 | 0.20 | -1.60 | down |
| 107056188 | 1.22 | 0.41 | -1.61 | down |
| 107057451 | 2.07 | 0.62 | -1.70 | down |
| BGI_novel_G000281 | 0.31 | 0.10 | -1.72 | down |
| BGI_novel_G000719 | 0.75 | 0.22 | -1.78 | down |
| 422630 | 7.70 | 2.16 | -1.83 | down |
| BGI_novel_G000714 | 0.72 | 0.20 | -1.88 | down |
| 101751836 | 0.58 | 0.15 | -1.92 | down |
| 107056344 | 2.15 | 0.44 | -2.00 | down |
| 107056478 | 0.74 | 0.17 | -2.11 | down |
| 419862 | 0.26 | 0.07 | -2.14 | down |
| 107055935 | 3.21 | 0.72 | -2.15 | down |
| 425328 | 2.72 | 0.59 | -2.21 | down |
| 107057181 | 1.60 | 0.34 | -2.23 | down |
| 101748210 | 0.68 | 0.14 | -2.26 | down |
| BGI_novel_G000033 | 0.43 | 0.09 | -2.31 | down |
| BGI_novel_G000340 | 0.22 | 0.05 | -2.31 | down |
| 428970 | 0.11 | 0.02 | -2.33 | down |
| 423566 | 0.56 | 0.11 | -2.37 | down |
| 107056867 | 0.58 | 0.13 | -2.37 | down |
| 421298 | 0.27 | 0.03 | -2.97 | down |
| 107049604 | 0.44 | 0.04 | -3.56 | down |
| 107057291 | 0.25 | 0.03 | -3.62 | down |
| BGI_novel_G000087 | 0.38 | 0.03 | -3.74 | down |
| BGI_novel_G000588 | 0.73 | 0.04 | -4.04 | down |
| BGI_novel_G000618 | 3.04 | 0.19 | -4.05 | down |
| BGI_novel_G000280 | 0.47 | 0.02 | -4.35 | down |
| BGI_novel_G000671 | 1.14 | 0 | -4.75 | down |
| BGI_novel_G000448 | 0.13 | 0 | -4.79 | down |
| BGI_novel_G000493 | 0.13 | 0 | -4.90 | down |
| 422750 | 0.19 | 0 | -5.11 | down |
| BGI_novel_G000616 | 0.27 | 0 | -5.21 | down |
| 107057211 | 0.44 | 0 | -5.36 | down |
| BGI_novel_G000444 | 1.15 | 0 | -5.54 | down |
| 428689 | 0.32 | 0 | -5.55 | down |
| 107057069 | 0.86 | 0 | -6.39 | down |
| BGI_novel_G000771 | 9.95 | 0 | -8.08 | down |
| 107057377 | 2.64 | 0 | -8.59 | down |
| 421150 | 3.69 | 0 | -10.26 | down |
