# Supplementary material for: Interactions Among Expressed MicroRNAs and mRNAs in the Early Stages of Fowl Adenovirus Aerotype 4-Infected Leghorn Male Hepatocellular Cells
Source: Front Microbiol. 2020 May 19;11:831. doi: 10.3389/fmicb.2020.00831 (PMC7248314; doi:10.3389/fmicb.2020.00831)
Supplement: FIGURE S1 — Hierarchical clustering analysis of 784 differentially expressed miRNAs at three time points. The color in the heat map represents the gene expression changes. Red indicates the upregulation of gene expression, blue indicates the downregulation of expression, the darker color indicates a notable degree of differential gene expression, and white indicates no activity. [file Data_Sheet_1.ZIP › supplementary files/Table S6.doc]

TABLE S6 Differentially expressed mRNAs between mock and infected LMHs at 120 min

| **gene id** | **Average fpkm**  **(Mock)** | **Average fpkm**  **(FAdV4_30)** | **log2Ratio**  **(FAdV4_30/Mock)** | **Up down regulation** |
| --- | --- | --- | --- | --- |
| BGI_novel_G000122 | 1.68 | 0 | -9.90 | down |
| BGI_novel_G000618 | 3.04 | 0 | -8.59 | down |
| 107057144 | 1.73 | 0 | -7.99 | down |
| 420028 | 0.83 | 0 | -7.97 | down |
| 101748210 | 0.68 | 0 | -7.76 | down |
| 107057451 | 2.07 | 0 | -7.08 | down |
| 107050024 | 0.70 | 0 | -6.07 | down |
| 107049120 | 0.44 | 0 | -5.81 | down |
| 107056202 | 1.95 | 0.04 | -5.62 | down |
| 100858554 | 0.67 | 0.01 | -5.56 | down |
| 100857331 | 0.32 | 0 | -5.56 | down |
| BGI_novel_G000616 | 0.27 | 0 | -5.25 | down |
| 422750 | 0.19 | 0 | -5.15 | down |
| 107054963 | 0.12 | 0 | -5.13 | down |
| 107049197 | 0.13 | 0 | -4.96 | down |
| BGI_novel_G000493 | 0.13 | 0 | -4.93 | down |
| 107055980 | 0.16 | 0 | -4.87 | down |
| 107055712 | 0.39 | 0.01 | -4.85 | down |
| BGI_novel_G000448 | 0.13 | 0 | -4.83 | down |
| BGI_novel_G000671 | 1.14 | 0 | -4.79 | down |
| 416682 | 0.18 | 0 | -4.59 | down |
| BGI_novel_G000761 | 0.96 | 0 | -4.56 | down |
| BGI_novel_G000591 | 0.49 | 0.02 | -4.47 | down |
| 427689 | 0.63 | 0.04 | -4.00 | down |
| BGI_novel_G000740 | 2.30 | 0.20 | -3.66 | down |
| BGI_novel_G000135 | 0.23 | 0.03 | -3.01 | down |
| BGI_novel_G000809 | 0.40 | 0.06 | -2.88 | down |
| 100857405 | 0.82 | 0.14 | -2.63 | down |
| 107053956 | 1.69 | 0.32 | -2.59 | down |
| 421298 | 0.27 | 0.04 | -2.59 | down |
| BGI_novel_G000397 | 1.20 | 0.20 | -2.58 | down |
| 101751836 | 0.58 | 0.10 | -2.46 | down |
| 419862 | 0.26 | 0.05 | -2.44 | down |
| 107049310 | 1.25 | 0.24 | -2.42 | down |
| 107056404 | 0.42 | 0.06 | -2.41 | down |
| 100858381 | 0.81 | 0.16 | -2.33 | down |
| 395282 | 49.13 | 10.64 | -2.24 | down |
| 100857744 | 0.34 | 0.07 | -2.17 | down |
| 428689 | 0.32 | 0.07 | -2.13 | down |
| 428413 | 64.80 | 15.61 | -2.07 | down |
| 427983 | 0.06 | 0.01 | -2.06 | down |
| 769704 | 1.15 | 0.37 | -2.04 | down |
| 107056344 | 2.15 | 0.42 | -2.03 | down |
| BGI_novel_G000073 | 0.32 | 0.08 | -2.02 | down |
| 395465 | 18.82 | 4.76 | -1.99 | down |
| 101749510 | 0.98 | 0.24 | -1.98 | down |
| 417139 | 0.73 | 0.19 | -1.94 | down |
| BGI_novel_G000087 | 0.38 | 0.10 | -1.93 | down |
| 100857573 | 0.09 | 0.02 | -1.90 | down |
| BGI_novel_G000150 | 1.73 | 0.46 | -1.90 | down |
| 770405 | 1.09 | 0.31 | -1.85 | down |
| 429012 | 0.26 | 0.07 | -1.84 | down |
| 418924 | 0.99 | 0.29 | -1.84 | down |
| 420859 | 0.19 | 0.05 | -1.83 | down |
| 107055935 | 3.21 | 0.90 | -1.81 | down |
| 107054817 | 0.85 | 0.26 | -1.68 | down |
| 107055332 | 1.60 | 0.51 | -1.66 | down |
| 373885 | 1.20 | 0.38 | -1.66 | down |
| BGI_novel_G000235 | 1.09 | 0.35 | -1.65 | down |
| BGI_novel_G000156 | 2.15 | 0.69 | -1.64 | down |
| 428383 | 0.34 | 0.11 | -1.61 | down |
| 395790 | 6.74 | 2.23 | -1.60 | down |
| 768736 | 1.44 | 0.49 | -1.60 | down |
| 107057134 | 1.49 | 0.53 | -1.59 | down |
| 107056507 | 19.06 | 6.45 | -1.59 | down |
| 395852 | 97.79 | 33.40 | -1.56 | down |
| 107057400 | 0.66 | 0.21 | -1.56 | down |
| 395322 | 0.41 | 0.14 | -1.55 | down |
| 107056125 | 3.30 | 1.15 | -1.55 | down |
| 107057031 | 9.79 | 3.44 | -1.52 | down |
| BGI_novel_G000766 | 1.82 | 0.63 | -1.50 | down |
| 107056386 | 0.83 | 0.26 | -1.49 | down |
| BGI_novel_G000228 | 3.69 | 1.34 | -1.49 | down |
| 427260 | 1.65 | 0.59 | -1.48 | down |
| 395281 | 143.47 | 52.32 | -1.47 | down |
| 107057508 | 1.81 | 0.61 | -1.47 | down |
| 101750381 | 0.59 | 0.22 | -1.46 | down |
| 771537 | 0.80 | 0.29 | -1.46 | down |
| BGI_novel_G000865 | 2.43 | 0.86 | -1.46 | down |
| 423175 | 0.11 | 0.04 | -1.45 | down |
| 422630 | 7.70 | 2.84 | -1.44 | down |
| BGI_novel_G000148 | 1.16 | 0.43 | -1.44 | down |
| 419937 | 1.22 | 0.47 | -1.41 | down |
| 107057069 | 0.86 | 0.32 | -1.40 | down |
| 107053848 | 3.13 | 1.20 | -1.40 | down |
| 408042 | 2.21 | 0.84 | -1.36 | down |
| 101749507 | 0.59 | 0.23 | -1.35 | down |
| 416967 | 21.59 | 8.48 | -1.35 | down |
| 418746 | 0.71 | 0.28 | -1.34 | down |
| BGI_novel_G000596 | 0.48 | 0.19 | -1.31 | down |
| BGI_novel_G000724 | 1.01 | 0.40 | -1.31 | down |
| 420642 | 0.13 | 0.05 | -1.31 | down |
| 771538 | 0.55 | 0.23 | -1.31 | down |
| 425623 | 0.62 | 0.25 | -1.29 | down |
| 101752215 | 1.48 | 0.61 | -1.28 | down |
| BGI_novel_G000281 | 0.31 | 0.13 | -1.28 | down |
| 420030 | 0.24 | 0.10 | -1.28 | down |
| 107056500 | 1.77 | 0.75 | -1.26 | down |
| BGI_novel_G000635 | 1.78 | 0.75 | -1.25 | down |
| 420094 | 0.24 | 0.10 | -1.25 | down |
| 107057163 | 0.78 | 0.34 | -1.24 | down |
| 107051097 | 7.05 | 3.09 | -1.23 | down |
| 428477 | 4.19 | 1.78 | -1.23 | down |
| 425328 | 2.72 | 1.17 | -1.23 | down |
| 107054719 | 1.72 | 0.75 | -1.23 | down |
| BGI_novel_G000107 | 1.88 | 0.81 | -1.21 | down |
| 100858896 | 0.85 | 0.37 | -1.20 | down |
| 428769 | 1.46 | 0.62 | -1.20 | down |
| 419029 | 2.28 | 1.02 | -1.18 | down |
| 427588 | 11.95 | 5.28 | -1.17 | down |
| 422251 | 0.51 | 0.27 | -1.17 | down |
| 417126 | 1.16 | 0.52 | -1.17 | down |
| BGI_novel_G000601 | 1.06 | 0.47 | -1.16 | down |
| 101749447 | 0.73 | 0.33 | -1.16 | down |
| BGI_novel_G000956 | 2.47 | 1.12 | -1.16 | down |
| BGI_novel_G000062 | 0.93 | 0.41 | -1.16 | down |
| 418976 | 0.35 | 0.16 | -1.15 | down |
| 107052854 | 1.97 | 0.89 | -1.14 | down |
| BGI_novel_G000031 | 0.47 | 0.22 | -1.13 | down |
| 107056065 | 1.06 | 0.49 | -1.12 | down |
| 420510 | 1.98 | 0.92 | -1.12 | down |
| 107057088 | 1.09 | 0.50 | -1.12 | down |
| 101748084 | 1.12 | 0.51 | -1.11 | down |
| 395447 | 0.52 | 0.24 | -1.11 | down |
| 107052502 | 1.19 | 0.56 | -1.11 | down |
| 100858354 | 33.15 | 15.70 | -1.10 | down |
| 100858777 | 0.48 | 0.23 | -1.09 | down |
| 395700 | 0.50 | 0.28 | -1.09 | down |
| 424561 | 14.74 | 6.80 | -1.08 | down |
| 101752023 | 2.03 | 0.97 | -1.07 | down |
| 100859106 | 0.71 | 0.34 | -1.07 | down |
| 416922 | 1.88 | 0.93 | -1.06 | down |
| BGI_novel_G000069 | 1.40 | 0.68 | -1.06 | down |
| 424892 | 0.71 | 0.34 | -1.05 | down |
| BGI_novel_G000504 | 1.84 | 0.90 | -1.05 | down |
| 428837 | 1.35 | 0.68 | -1.05 | down |
| 416256 | 0.74 | 0.35 | -1.05 | down |
| 417504 | 0.27 | 0.13 | -1.04 | down |
| 424821 | 0.63 | 0.31 | -1.04 | down |
| BGI_novel_G000151 | 1.94 | 0.95 | -1.03 | down |
| 428388 | 0.23 | 0.12 | -1.03 | down |
| 427756 | 1.47 | 0.77 | -1.03 | down |
| 416335 | 4.96 | 2.43 | -1.03 | down |
| 425491 | 2.32 | 1.22 | -1.02 | down |
| 107050026 | 7.25 | 3.62 | -1.02 | down |
| 768936 | 0.44 | 0.22 | -1.02 | down |
| 420556 | 7.11 | 3.74 | -1.01 | down |
| BGI_novel_G000339 | 0.62 | 0.31 | -1.01 | down |
| 426725 | 1.23 | 0.60 | -1.00 | down |
| 395346 | 0.43 | 0.21 | -1.00 | down |
| 107057199 | 2.91 | 1.47 | -1.00 | down |
| 424389 | 2.61 | 4.83 | 1.00 | up |
| 423083 | 24.73 | 49.88 | 1.01 | up |
| BGI_novel_G000338 | 2.52 | 5.10 | 1.01 | up |
| 429010 | 2.76 | 5.51 | 1.01 | up |
| 424523 | 6.15 | 12.41 | 1.01 | up |
| 415748 | 1.01 | 2.00 | 1.01 | up |
| 414143 | 0.75 | 1.73 | 1.01 | up |
| 107056140 | 3.77 | 7.12 | 1.01 | up |
| 107052763 | 11.08 | 22.49 | 1.01 | up |
| 417582 | 5.66 | 11.45 | 1.01 | up |
| 420815 | 7.85 | 15.82 | 1.01 | up |
| 421037 | 35.79 | 73.00 | 1.01 | up |
| 419209 | 2.84 | 5.81 | 1.01 | up |
| 396379 | 54.32 | 108.80 | 1.02 | up |
| 427146 | 2.06 | 4.18 | 1.02 | up |
| 395415 | 0.55 | 1.15 | 1.02 | up |
| 417794 | 2.28 | 4.88 | 1.02 | up |
| 101749888 | 10.30 | 21.01 | 1.03 | up |
| 424238 | 13.06 | 26.70 | 1.03 | up |
| 420082 | 4.41 | 9.02 | 1.03 | up |
| 427236 | 5.90 | 12.03 | 1.03 | up |
| 420389 | 0.15 | 0.31 | 1.03 | up |
| 418110 | 4.02 | 8.23 | 1.03 | up |
| 395133 | 9.51 | 19.72 | 1.03 | up |
| BGI_novel_G000032 | 0.19 | 0.38 | 1.03 | up |
| 425403 | 0.74 | 1.53 | 1.03 | up |
| 418724 | 2.88 | 5.90 | 1.03 | up |
| BGI_novel_G000316 | 5.34 | 11.13 | 1.03 | up |
| 373907 | 52.49 | 108.92 | 1.04 | up |
| 423603 | 0.67 | 1.29 | 1.04 | up |
| 101749375 | 9.79 | 20.08 | 1.04 | up |
| 419265 | 5.08 | 10.45 | 1.04 | up |
| 416579 | 1.72 | 3.55 | 1.04 | up |
| BGI_novel_G000299 | 1.07 | 2.22 | 1.04 | up |
| 416286 | 2.30 | 4.77 | 1.05 | up |
| 414831 | 1.61 | 3.27 | 1.05 | up |
| 418074 | 3.22 | 6.68 | 1.05 | up |
| 415992 | 5.65 | 11.72 | 1.05 | up |
| 417809 | 23.99 | 49.50 | 1.05 | up |
| 418589 | 12.37 | 25.71 | 1.05 | up |
| 771523 | 6.31 | 12.96 | 1.05 | up |
| 422375 | 10.33 | 21.02 | 1.05 | up |
| 420740 | 28.21 | 58.91 | 1.06 | up |
| 419932 | 23.42 | 48.76 | 1.06 | up |
| 396102 | 6.81 | 14.48 | 1.06 | up |
| 100858328 | 0.71 | 1.47 | 1.06 | up |
| BGI_novel_G000277 | 0.45 | 0.94 | 1.06 | up |
| 100858582 | 0.68 | 1.43 | 1.06 | up |
| 421689 | 0.31 | 0.64 | 1.06 | up |
| 107056025 | 0.27 | 0.57 | 1.06 | up |
| 427031 | 0.44 | 0.93 | 1.06 | up |
| 416102 | 0.73 | 1.63 | 1.06 | up |
| 427203 | 9.00 | 18.80 | 1.07 | up |
| 396134 | 9.90 | 20.93 | 1.07 | up |
| 768655 | 49.14 | 103.56 | 1.07 | up |
| 416693 | 48.13 | 102.26 | 1.07 | up |
| 395470 | 5.14 | 11.32 | 1.07 | up |
| 423541 | 9.16 | 19.35 | 1.08 | up |
| 426054 | 1.50 | 3.17 | 1.08 | up |
| 100857244 | 11.79 | 25.27 | 1.08 | up |
| 418761 | 15.36 | 34.43 | 1.08 | up |
| 395991 | 0.34 | 0.75 | 1.08 | up |
| 421747 | 2.91 | 6.19 | 1.09 | up |
| 395143 | 18.03 | 38.49 | 1.09 | up |
| 422829 | 11.57 | 20.44 | 1.09 | up |
| 427567 | 0.17 | 0.37 | 1.09 | up |
| 421948 | 1.64 | 3.92 | 1.09 | up |
| 415996 | 0.13 | 0.28 | 1.10 | up |
| 423432 | 0.45 | 0.98 | 1.10 | up |
| 427252 | 7.75 | 17.11 | 1.11 | up |
| 107050448 | 0.78 | 1.73 | 1.11 | up |
| 417147 | 12.27 | 26.42 | 1.11 | up |
| 417919 | 12.05 | 26.10 | 1.11 | up |
| 425900 | 10.62 | 23.00 | 1.11 | up |
| 100034745 | 3.43 | 7.48 | 1.11 | up |
| 420299 | 14.59 | 29.70 | 1.11 | up |
| 395925 | 0.80 | 1.69 | 1.12 | up |
| 421656 | 3.90 | 8.42 | 1.12 | up |
| 423241 | 1.37 | 3.11 | 1.12 | up |
| 421276 | 17.23 | 37.35 | 1.12 | up |
| 418939 | 0.09 | 0.20 | 1.12 | up |
| 416352 | 5.16 | 12.26 | 1.12 | up |
| 100857105 | 5.09 | 10.85 | 1.12 | up |
| 423516 | 0.03 | 0.07 | 1.13 | up |
| 396029 | 10.06 | 22.06 | 1.13 | up |
| 418406 | 3.57 | 7.83 | 1.13 | up |
| BGI_novel_G000788 | 0.70 | 1.53 | 1.13 | up |
| BGI_novel_G000574 | 17.42 | 38.26 | 1.13 | up |
| 428277 | 0.11 | 0.24 | 1.13 | up |
| 420673 | 4.74 | 10.47 | 1.14 | up |
| 396474 | 3.30 | 7.05 | 1.14 | up |
| 107049317 | 1.27 | 2.81 | 1.14 | up |
| 408027 | 162.22 | 363.39 | 1.15 | up |
| 424917 | 1.74 | 3.87 | 1.15 | up |
| 396390 | 22.24 | 49.93 | 1.15 | up |
| 417586 | 2.68 | 5.95 | 1.15 | up |
| 100857227 | 1.52 | 3.31 | 1.16 | up |
| 101749610 | 9.92 | 23.29 | 1.16 | up |
| 107057079 | 0.70 | 1.58 | 1.17 | up |
| 414893 | 0.08 | 0.18 | 1.17 | up |
| 769109 | 0.58 | 1.30 | 1.17 | up |
| BGI_novel_G000108 | 0.31 | 0.70 | 1.17 | up |
| 395674 | 25.71 | 58.84 | 1.17 | up |
| 418013 | 3.99 | 9.03 | 1.17 | up |
| 771032 | 23.17 | 52.67 | 1.17 | up |
| 770254 | 1.96 | 4.47 | 1.18 | up |
| 107055280 | 0.65 | 1.49 | 1.18 | up |
| 769506 | 0.70 | 1.65 | 1.18 | up |
| 373904 | 19.06 | 45.52 | 1.18 | up |
| 426897 | 0.42 | 0.30 | 1.18 | up |
| 770260 | 0.27 | 0.62 | 1.18 | up |
| 422832 | 2.11 | 4.81 | 1.18 | up |
| 100858576 | 16.35 | 36.79 | 1.18 | up |
| 420570 | 139.83 | 319.80 | 1.19 | up |
| 425711 | 31.76 | 72.86 | 1.19 | up |
| 395411 | 11.03 | 25.37 | 1.20 | up |
| 395519 | 36.06 | 83.32 | 1.20 | up |
| 420309 | 3.36 | 7.76 | 1.20 | up |
| 420419 | 15.65 | 36.13 | 1.20 | up |
| 427990 | 0.06 | 0.13 | 1.20 | up |
| 421845 | 5.13 | 11.60 | 1.20 | up |
| 418496 | 1.24 | 2.99 | 1.20 | up |
| 422884 | 6.92 | 16.05 | 1.20 | up |
| 423978 | 8.69 | 20 | 1.21 | up |
| BGI_novel_G000177 | 0.91 | 2.12 | 1.21 | up |
| 100538348 | 36.29 | 66.08 | 1.21 | up |
| 396105 | 1.04 | 2.44 | 1.21 | up |
| 423435 | 0.57 | 1.20 | 1.21 | up |
| 420104 | 7.78 | 18.14 | 1.21 | up |
| 419255 | 1.16 | 2.84 | 1.22 | up |
| 396126 | 1.98 | 4.57 | 1.22 | up |
| 416206 | 1.67 | 4.26 | 1.22 | up |
| 776013 | 5.29 | 12.41 | 1.23 | up |
| BGI_novel_G000090 | 0.59 | 1.41 | 1.23 | up |
| 422765 | 9.01 | 20.95 | 1.23 | up |
| 101751930 | 0.26 | 0.61 | 1.23 | up |
| 770435 | 2.45 | 5.80 | 1.23 | up |
| 416818 | 1.88 | 4.13 | 1.23 | up |
| 428166 | 15.77 | 40.33 | 1.24 | up |
| 421424 | 0.60 | 1.40 | 1.24 | up |
| 419327 | 3.52 | 8.32 | 1.24 | up |
| 430595 | 1.60 | 3.83 | 1.24 | up |
| 422347 | 28.75 | 68.12 | 1.24 | up |
| 418502 | 29.35 | 69.75 | 1.24 | up |
| 395750 | 7.63 | 18.16 | 1.25 | up |
| 107055705 | 0.43 | 1.05 | 1.25 | up |
| 769261 | 1.43 | 2.69 | 1.25 | up |
| 416009 | 2.15 | 5.20 | 1.26 | up |
| BGI_novel_G000517 | 0.27 | 0.64 | 1.26 | up |
| 101750986 | 49.29 | 118.80 | 1.26 | up |
| 107049140 | 6.48 | 16.32 | 1.26 | up |
| 775985 | 6.85 | 17.14 | 1.27 | up |
| 100857974 | 1.49 | 3.60 | 1.27 | up |
| 101750587 | 0.85 | 2.09 | 1.27 | up |
| 429484 | 3.92 | 10.62 | 1.27 | up |
| 417230 | 12.57 | 30.35 | 1.27 | up |
| 427150 | 7.59 | 18.38 | 1.27 | up |
| 421369 | 11.55 | 28.01 | 1.27 | up |
| BGI_novel_G000575 | 1.64 | 4.00 | 1.28 | up |
| BGI_novel_G000458 | 0.79 | 1.94 | 1.28 | up |
| 417460 | 0.15 | 0.38 | 1.28 | up |
| 422065 | 1.86 | 4.61 | 1.28 | up |
| 417567 | 0.67 | 1.63 | 1.28 | up |
| 100859777 | 1.51 | 3.67 | 1.29 | up |
| 422284 | 1.28 | 3.15 | 1.29 | up |
| 101747338 | 10.72 | 26.26 | 1.29 | up |
| 769469 | 2.90 | 7.33 | 1.29 | up |
| 417992 | 16.63 | 40.76 | 1.29 | up |
| 422230 | 2.28 | 5.60 | 1.29 | up |
| 100859250 | 0.09 | 0.22 | 1.30 | up |
| 427250 | 2.05 | 5.04 | 1.30 | up |
| 107055352 | 23.70 | 58.68 | 1.30 | up |
| 373902 | 21.84 | 54.41 | 1.30 | up |
| 100857774 | 1.40 | 3.51 | 1.30 | up |
| 419428 | 0.20 | 0.50 | 1.31 | up |
| 418404 | 7.30 | 18.10 | 1.31 | up |
| 107055314 | 1.29 | 3.22 | 1.32 | up |
| 422740 | 0.67 | 1.69 | 1.32 | up |
| 445340 | 4.34 | 11.71 | 1.32 | up |
| 420185 | 22.99 | 60.51 | 1.32 | up |
| 107055808 | 0.26 | 0.71 | 1.32 | up |
| 396005 | 42.16 | 119.83 | 1.32 | up |
| 100499189 | 3.25 | 7.91 | 1.32 | up |
| 420856 | 6.41 | 16.09 | 1.33 | up |
| 107056374 | 0.29 | 0.72 | 1.33 | up |
| 768353 | 0.58 | 1.48 | 1.33 | up |
| 416800 | 5.27 | 15.52 | 1.34 | up |
| 420261 | 1.32 | 3.36 | 1.35 | up |
| 415530 | 0.98 | 1.96 | 1.35 | up |
| 776024 | 1.26 | 3.20 | 1.35 | up |
| 424191 | 0.15 | 0.43 | 1.35 | up |
| 395910 | 0.44 | 1.15 | 1.36 | up |
| 425840 | 8.31 | 21.38 | 1.36 | up |
| 428460 | 1.86 | 4.82 | 1.37 | up |
| 422752 | 0.12 | 0.29 | 1.37 | up |
| 423499 | 2.57 | 6.66 | 1.37 | up |
| 378803 | 2.37 | 6.09 | 1.37 | up |
| 395093 | 0.17 | 0.43 | 1.37 | up |
| 107057396 | 0.13 | 0.33 | 1.38 | up |
| 395491 | 0.70 | 1.85 | 1.38 | up |
| 427183 | 0.28 | 0.77 | 1.38 | up |
| 374135 | 0.74 | 1.96 | 1.39 | up |
| 416159 | 9.77 | 25.75 | 1.39 | up |
| 100859913 | 2.56 | 6.77 | 1.39 | up |
| 395634 | 2.67 | 6.93 | 1.40 | up |
| 101749609 | 0.37 | 0.98 | 1.40 | up |
| 428790 | 0.97 | 2.54 | 1.41 | up |
| 770026 | 0.18 | 0.46 | 1.41 | up |
| 396472 | 0.22 | 0.56 | 1.41 | up |
| 396489 | 20.16 | 54.15 | 1.43 | up |
| 417228 | 5.13 | 11.90 | 1.43 | up |
| 428171 | 1.18 | 3.19 | 1.43 | up |
| 100857346 | 0.22 | 0.58 | 1.43 | up |
| 431167 | 1.25 | 3.58 | 1.44 | up |
| 769474 | 0.20 | 0.56 | 1.44 | up |
| 423932 | 2.54 | 6.74 | 1.44 | up |
| 416150 | 0.15 | 0.41 | 1.45 | up |
| 419411 | 0.34 | 0.94 | 1.45 | up |
| 423931 | 17.22 | 47.20 | 1.45 | up |
| 396152 | 0.63 | 1.75 | 1.45 | up |
| 419805 | 12.64 | 33.70 | 1.45 | up |
| 395873 | 0.63 | 1.66 | 1.46 | up |
| 107057463 | 0.18 | 0.51 | 1.47 | up |
| 408036 | 2.52 | 7.09 | 1.47 | up |
| 772006 | 0.19 | 0.52 | 1.47 | up |
| 107055350 | 0.22 | 0.61 | 1.48 | up |
| 770453 | 2.74 | 6.81 | 1.49 | up |
| 100859173 | 2.73 | 7.67 | 1.49 | up |
| 396185 | 48.16 | 136.21 | 1.49 | up |
| 429363 | 0.43 | 1.22 | 1.49 | up |
| 419309 | 0.05 | 0.15 | 1.49 | up |
| 101752124 | 0.21 | 0.58 | 1.50 | up |
| 422166 | 5.29 | 15.10 | 1.51 | up |
| 428970 | 0.11 | 0.32 | 1.51 | up |
| 427979 | 0.12 | 0.39 | 1.52 | up |
| 107057253 | 0.61 | 1.74 | 1.52 | up |
| 418325 | 9.21 | 26.61 | 1.52 | up |
| 423079 | 1.26 | 3.94 | 1.53 | up |
| 101750341 | 0.05 | 0.14 | 1.53 | up |
| 101750892 | 0.16 | 0.48 | 1.53 | up |
| 100858838 | 8.18 | 24.27 | 1.53 | up |
| 424826 | 22.80 | 66.02 | 1.53 | up |
| 374192 | 6.89 | 19.99 | 1.53 | up |
| 424435 | 0.20 | 0.58 | 1.53 | up |
| 107049350 | 0.67 | 1.96 | 1.53 | up |
| 107053339 | 0.23 | 0.65 | 1.54 | up |
| 426211 | 5.30 | 15.31 | 1.54 | up |
| 421012 | 0.23 | 0.68 | 1.54 | up |
| 101749060 | 22.08 | 66.16 | 1.55 | up |
| 374187 | 0.58 | 1.72 | 1.56 | up |
| BGI_novel_G000637 | 0.44 | 1.29 | 1.56 | up |
| 428930 | 1.05 | 3.08 | 1.56 | up |
| 424984 | 4.02 | 12.43 | 1.57 | up |
| 107057150 | 0.43 | 1.30 | 1.57 | up |
| 419068 | 2.33 | 6.90 | 1.57 | up |
| 101751827 | 27.25 | 81.43 | 1.57 | up |
| 100858332 | 1.58 | 4.82 | 1.58 | up |
| 420097 | 0.22 | 0.67 | 1.58 | up |
| 419882 | 0.22 | 0.67 | 1.58 | up |
| 428172 | 0.77 | 1.99 | 1.58 | up |
| 396043 | 4.54 | 13.74 | 1.59 | up |
| BGI_novel_G000472 | 0.37 | 1.10 | 1.60 | up |
| 428649 | 0.21 | 0.78 | 1.60 | up |
| 429054 | 0.32 | 0.97 | 1.61 | up |
| 395799 | 0.10 | 0.40 | 1.61 | up |
| 395528 | 14.55 | 44.89 | 1.61 | up |
| 415790 | 0.31 | 0.97 | 1.62 | up |
| 107054668 | 0.30 | 1.32 | 1.62 | up |
| 417793 | 0.42 | 1.30 | 1.62 | up |
| 424586 | 4.75 | 14.71 | 1.63 | up |
| 419753 | 3.72 | 11.52 | 1.63 | up |
| 417783 | 5.78 | 18.25 | 1.65 | up |
| 425270 | 0.14 | 0.36 | 1.65 | up |
| 396235 | 0.85 | 2.56 | 1.66 | up |
| 403121 | 0.45 | 1.16 | 1.66 | up |
| 396165 | 0.16 | 0.52 | 1.66 | up |
| 429152 | 0.31 | 0.99 | 1.66 | up |
| 418411 | 0.13 | 0.25 | 1.67 | up |
| 768977 | 0.71 | 2.36 | 1.67 | up |
| 428435 | 0.75 | 2.21 | 1.68 | up |
| 427564 | 0.12 | 0.35 | 1.69 | up |
| 395625 | 0.85 | 2.75 | 1.69 | up |
| 424198 | 0.03 | 0.11 | 1.69 | up |
| 427643 | 0.34 | 1.17 | 1.69 | up |
| 418616 | 2.49 | 8.07 | 1.70 | up |
| 396332 | 0.12 | 0.35 | 1.70 | up |
| 378779 | 7.91 | 26.37 | 1.70 | up |
| 395583 | 9.62 | 31.82 | 1.71 | up |
| 418963 | 3.31 | 10.91 | 1.71 | up |
| 422934 | 5.57 | 16.68 | 1.72 | up |
| 395768 | 4.74 | 15.66 | 1.72 | up |
| 428445 | 6.72 | 22.72 | 1.72 | up |
| 428906 | 3.58 | 11.78 | 1.72 | up |
| BGI_novel_G000725 | 0.17 | 0.57 | 1.73 | up |
| 373987 | 2.19 | 7.33 | 1.74 | up |
| 422487 | 0.73 | 2.47 | 1.75 | up |
| 107052930 | 23.49 | 79.06 | 1.75 | up |
| 107049003 | 0.04 | 0.11 | 1.75 | up |
| 419618 | 5.62 | 19.13 | 1.76 | up |
| 428752 | 9.25 | 31.67 | 1.76 | up |
| 396434 | 0.52 | 1.77 | 1.77 | up |
| 374059 | 9.48 | 32.55 | 1.77 | up |
| 771148 | 0.13 | 0.47 | 1.78 | up |
| 415686 | 0.35 | 1.22 | 1.78 | up |
| BGI_novel_G000892 | 0.52 | 1.83 | 1.79 | up |
| 428897 | 1.45 | 5.01 | 1.79 | up |
| 430666 | 0.11 | 0.37 | 1.80 | up |
| 107054848 | 0.19 | 0.67 | 1.80 | up |
| 396481 | 4.23 | 15.19 | 1.81 | up |
| 417960 | 3.31 | 11.53 | 1.81 | up |
| 396093 | 18.98 | 67.46 | 1.82 | up |
| 395326 | 8.33 | 29.48 | 1.82 | up |
| 100873158 | 6.85 | 24.48 | 1.83 | up |
| 100859283 | 0.79 | 2.84 | 1.84 | up |
| 101748744 | 0.13 | 0.42 | 1.84 | up |
| 419806 | 0.23 | 0.86 | 1.85 | up |
| 421340 | 3.17 | 11.43 | 1.85 | up |
| 429089 | 3.58 | 12.99 | 1.85 | up |
| 424004 | 5.30 | 19.19 | 1.85 | up |
| 101748386 | 0.05 | 0.19 | 1.85 | up |
| 100379662 | 0.44 | 1.61 | 1.85 | up |
| 421333 | 5.84 | 21.20 | 1.87 | up |
| 101751348 | 0.08 | 0.27 | 1.87 | up |
| 374272 | 2.66 | 9.80 | 1.88 | up |
| 424402 | 0.88 | 3.23 | 1.88 | up |
| 100858060 | 22.39 | 82.62 | 1.89 | up |
| 422540 | 3.07 | 11.54 | 1.89 | up |
| 425507 | 0.12 | 0.45 | 1.90 | up |
| 395219 | 6.86 | 26.83 | 1.91 | up |
| 426312 | 0.18 | 0.67 | 1.91 | up |
| 421815 | 0.09 | 0.33 | 1.92 | up |
| 107049320 | 0.29 | 1.07 | 1.92 | up |
| 418818 | 17.03 | 65.12 | 1.93 | up |
| 395707 | 6.72 | 24.09 | 1.93 | up |
| 426108 | 0.52 | 2.10 | 1.93 | up |
| 378912 | 13.37 | 51.31 | 1.93 | up |
| 416630 | 1.86 | 7.40 | 1.95 | up |
| 395329 | 3.48 | 12.88 | 1.96 | up |
| 768536 | 2.96 | 11.71 | 1.97 | up |
| 395939 | 29.09 | 115.67 | 1.98 | up |
| 395584 | 0.57 | 2.32 | 2.01 | up |
| 771278 | 2.17 | 9.73 | 2.01 | up |
| 421684 | 0.89 | 3.50 | 2.02 | up |
| 107052968 | 0.36 | 1.47 | 2.03 | up |
| 396385 | 54.89 | 225.06 | 2.03 | up |
| 374212 | 0.10 | 0.40 | 2.04 | up |
| 101752065 | 0.05 | 0.20 | 2.05 | up |
| 395354 | 21.13 | 87.60 | 2.05 | up |
| 395453 | 0.50 | 2.56 | 2.06 | up |
| 101751828 | 42.99 | 179.40 | 2.06 | up |
| 424312 | 14.70 | 61.51 | 2.06 | up |
| 418529 | 0.13 | 0.55 | 2.07 | up |
| BGI_novel_G000959 | 0.12 | 0.49 | 2.08 | up |
| 417461 | 0.09 | 0.37 | 2.08 | up |
| 107056392 | 0.36 | 1.63 | 2.09 | up |
| 396216 | 0.19 | 0.82 | 2.09 | up |
| 396204 | 0.07 | 0.31 | 2.10 | up |
| 427971 | 1.03 | 4.44 | 2.10 | up |
| 426820 | 0.17 | 0.67 | 2.10 | up |
| 378903 | 14.01 | 66.88 | 2.12 | up |
| 395827 | 0.08 | 0.39 | 2.13 | up |
| 378914 | 2.09 | 9.24 | 2.13 | up |
| 431604 | 0.04 | 0.18 | 2.14 | up |
| 417807 | 42.81 | 191.11 | 2.15 | up |
| 418126 | 0.05 | 0.20 | 2.15 | up |
| 395973 | 0.14 | 0.63 | 2.15 | up |
| 107056873 | 0.07 | 0.33 | 2.16 | up |
| 395559 | 1.94 | 9.21 | 2.17 | up |
| 422389 | 2.53 | 11.51 | 2.17 | up |
| 421672 | 1.89 | 8.67 | 2.19 | up |
| 768438 | 0.05 | 0.14 | 2.19 | up |
| 768922 | 0.82 | 3.76 | 2.20 | up |
| 107057333 | 0.17 | 0.76 | 2.20 | up |
| 395654 | 5.63 | 26.37 | 2.21 | up |
| 427937 | 4.04 | 19.01 | 2.21 | up |
| BGI_novel_G000078 | 0.08 | 0.36 | 2.22 | up |
| 107056699 | 0.55 | 2.52 | 2.22 | up |
| 770670 | 1.63 | 7.66 | 2.22 | up |
| 395483 | 0.71 | 3.37 | 2.23 | up |
| 107049019 | 0.03 | 0.15 | 2.23 | up |
| 424912 | 7.22 | 34.73 | 2.25 | up |
| 396338 | 0.10 | 0.46 | 2.26 | up |
| 100857932 | 5.12 | 24.78 | 2.26 | up |
| 373931 | 62.63 | 301.99 | 2.26 | up |
| 771550 | 6.05 | 29.64 | 2.29 | up |
| 423444 | 0.83 | 4.37 | 2.33 | up |
| 424883 | 74.72 | 378.34 | 2.33 | up |
| 100859744 | 0.22 | 1.10 | 2.35 | up |
| 378919 | 8.58 | 43.97 | 2.35 | up |
| 768907 | 0.01 | 0.06 | 2.37 | up |
| 423088 | 53.67 | 284.82 | 2.39 | up |
| 418736 | 2.03 | 10.75 | 2.40 | up |
| 101750112 | 0.01 | 0.05 | 2.40 | up |
| 395663 | 1.84 | 7.37 | 2.41 | up |
| 101749459 | 2.26 | 12.22 | 2.44 | up |
| 420479 | 1.23 | 6.92 | 2.50 | up |
| 416012 | 0.05 | 0.28 | 2.50 | up |
| 107049198 | 0.06 | 0.30 | 2.50 | up |
| 107056060 | 0.14 | 0.77 | 2.50 | up |
| 418247 | 14.66 | 89.68 | 2.51 | up |
| BGI_novel_G000088 | 0.07 | 0.41 | 2.60 | up |
| 427107 | 4.97 | 30.52 | 2.62 | up |
| 107049468 | 5.89 | 37.71 | 2.66 | up |
| 107049127 | 0.09 | 0.55 | 2.66 | up |
| 107056685 | 0.14 | 0.91 | 2.67 | up |
| 768821 | 0.12 | 0.79 | 2.69 | up |
| 404539 | 0.06 | 0.42 | 2.72 | up |
| 396250 | 3.90 | 26.00 | 2.73 | up |
| 101751752 | 0.02 | 0.12 | 2.73 | up |
| 107056986 | 0.18 | 1.24 | 2.77 | up |
| 421600 | 3.44 | 23.36 | 2.78 | up |
| 396119 | 6.81 | 48.55 | 2.83 | up |
| 423602 | 0.30 | 2.21 | 2.86 | up |
| 107050017 | 2.18 | 15.90 | 2.86 | up |
| 107055630 | 0.02 | 0.15 | 2.86 | up |
| 432368 | 0.36 | 2.63 | 2.87 | up |
| 423890 | 6.52 | 48.17 | 2.88 | up |
| BGI_novel_G000250 | 0.91 | 6.76 | 2.89 | up |
| 421416 | 7.59 | 57.31 | 2.90 | up |
| 427805 | 0.06 | 0.45 | 2.90 | up |
| 396384 | 1.37 | 10.71 | 2.95 | up |
| 423225 | 0.23 | 1.82 | 2.99 | up |
| 428386 | 25.75 | 213.18 | 3.05 | up |
| 419364 | 15.71 | 135.85 | 3.11 | up |
| 420996 | 6.34 | 56.20 | 3.17 | up |
| 396451 | 0.90 | 8.39 | 3.19 | up |
| 424320 | 1.71 | 15.86 | 3.19 | up |
| 395190 | 0.03 | 0.24 | 3.32 | up |
| 107056031 | 0.02 | 0.22 | 3.35 | up |
| 107056190 | 0.06 | 0.61 | 3.38 | up |
| 107055390 | 4.70 | 50.65 | 3.42 | up |
| 395210 | 1.33 | 14.55 | 3.44 | up |
| 395299 | 4.54 | 49.72 | 3.45 | up |
| 769132 | 0.01 | 0.12 | 3.52 | up |
| 107057045 | 0.03 | 0.32 | 3.55 | up |
| 418479 | 1.76 | 21.94 | 3.64 | up |
| 395075 | 0 | 0.08 | 3.70 | up |
| 428070 | 0.05 | 0.61 | 3.76 | up |
| 424562 | 0 | 0.06 | 3.76 | up |
| 428665 | 0.01 | 0.21 | 3.80 | up |
| 395335 | 2.17 | 31.05 | 3.83 | up |
| 424409 | 5.24 | 74.80 | 3.83 | up |
| 395594 | 0.24 | 3.69 | 3.91 | up |
| 100857380 | 1.69 | 25.75 | 3.92 | up |
| 420486 | 0.01 | 0.12 | 3.94 | up |
| 420463 | 9.37 | 150.16 | 4.00 | up |
| 102725543 | 0.83 | 15.19 | 4.18 | up |
| 107056355 | 0.15 | 2.76 | 4.23 | up |
| 427249 | 0 | 0.04 | 4.24 | up |
| 107049603 | 0.56 | 11.54 | 4.36 | up |
| 396495 | 0.57 | 13.35 | 4.55 | up |
| 107056104 | 0.02 | 0.42 | 4.64 | up |
| BGI_novel_G000055 | 0 | 0.06 | 4.69 | up |
| 395872 | 0.45 | 12.00 | 4.72 | up |
| 422950 | 0.07 | 2.17 | 4.89 | up |
| BGI_novel_G000860 | 0 | 0.11 | 5.20 | up |
| 107053337 | 0 | 0.20 | 5.20 | up |
| BGI_novel_G000681 | 0 | 0.11 | 5.27 | up |
| 107053497 | 0 | 0.22 | 5.34 | up |
| 107057357 | 0 | 0.26 | 5.39 | up |
| 101750527 | 0 | 0.08 | 5.58 | up |
| 103214197 | 0 | 0.32 | 5.62 | up |
| 107054654 | 0 | 0.12 | 5.69 | up |
| 417553 | 0 | 0.44 | 6.01 | up |
| BGI_novel_G000967 | 0 | 0.71 | 6.05 | up |
| 419933 | 0.01 | 0.41 | 6.06 | up |
| 100857910 | 0 | 0.52 | 6.19 | up |
| 107056385 | 0 | 0.62 | 6.90 | up |
| 420896 | 1.29 | 146.19 | 6.93 | up |
| 107049080 | 0 | 3.01 | 6.99 | up |
| 100859224 | 0 | 0.60 | 7.24 | up |
| 420505 | 0 | 0.35 | 7.26 | up |
| BGI_novel_G000767 | 0 | 1.12 | 7.45 | up |
| 426193 | 0 | 2.91 | 9.83 | up |
