# Supplementary material for: Interactions Among Expressed MicroRNAs and mRNAs in the Early Stages of Fowl Adenovirus Aerotype 4-Infected Leghorn Male Hepatocellular Cells
Source: Front Microbiol. 2020 May 19;11:831. doi: 10.3389/fmicb.2020.00831 (PMC7248314; doi:10.3389/fmicb.2020.00831)
Supplement: FIGURE S1 — Hierarchical clustering analysis of 784 differentially expressed miRNAs at three time points. The color in the heat map represents the gene expression changes. Red indicates the upregulation of gene expression, blue indicates the downregulation of expression, the darker color indicates a notable degree of differential gene expression, and white indicates no activity. [file Data_Sheet_1.ZIP › supplementary files/Table S8.doc]

TABLE S8 The negative miRNA-mRNA pairs between mock and infected LMHs at 60 min.

| **miRNA id** | **Target id** | **Symbol** | **miRNA Fold change** | **Target Fold change** | **Correlation (pearson)** |
| --- | --- | --- | --- | --- | --- |
| novel_mir271 | NM_204136.2 | EGR1 | -6.57 | 3.10 | -0.87 |
| novel_mir271 | XM_418053.5 | BTG2 | -6.57 | 2.29 | -0.86 |
| novel_mir276 | NM_001031037.1 | SERTAD2 | -6.29 | 1.26 | -0.82 |
| novel_mir120 | XM_015283427.1 | BCL2L11 | 3.30 | -1.49 | -0.80 |
| novel_mir569 | XM_015302340.1 | LOC107057304 | -1.27 | 2.42 | -0.78 |
| novel_mir820 | XM_418053.5 | BTG2 | -6.44 | 2.29 | -0.78 |
| novel_mir585 | XM_004946108.2 | LOC101747455 | 5.54 | -1.06 | -0.77 |
| novel_mir975 | XM_015297790.1 | EVA1B | -1.14 | 1.15 | -0.77 |
| novel_mir261 | XM_015301841.1 | LOC107056867 | 2.31 | -2.37 | -0.77 |
| gga-miR-6651-5p | XM_004937095.2 | LOC100857744 | 2.84 | -1.46 | -0.76 |
| novel_mir304 | XM_003640342.3 | PIM3 | -6.29 | 1.25 | -0.75 |
| novel_mir120 | XM_422391.5 | AK5 | 3.30 | -1.34 | -0.74 |
| novel_mir822 | XM_015290490.1 | IER5 | -1.35 | 1.51 | -0.74 |
| novel_mir822 | NM_001101037.1 | CENPA | -1.35 | 1.17 | -0.74 |
| novel_mir304 | XM_421754.5 | DUSP5 | -6.29 | 3.00 | -0.73 |
| novel_mir205 | XM_015297790.1 | EVA1B | -1.77 | 1.15 | -0.73 |
| gga-miR-12223-3p | XM_015278045.1 | SERTAD2 | -1.13 | 1.26 | -0.72 |
| novel_mir569 | XM_418053.5 | BTG2 | -1.27 | 2.29 | -0.72 |
| novel_mir735 | NM_205508.1 | FOS | -1.85 | 2.80 | -0.72 |
| novel_mir82 | XM_015286835.1 | LOC768811 | -1.74 | 1.76 | -0.72 |
| novel_mir271 | XM_003640342.3 | PIM3 | -6.57 | 1.25 | -0.72 |
| novel_mir709 | XM_015300578.1 | LOC426820 | -1.34 | 1.97 | -0.71 |
| novel_mir304 | XM_015300220.1 | LOC107055390 | -6.29 | 4.05 | -0.71 |
| novel_mir822 | XM_015300220.1 | LOC107055390 | -1.35 | 4.05 | -0.71 |
| novel_mir822 | XM_015282155.1 | PTF1A | -1.35 | 1.34 | -0.70 |
| novel_mir692 | XM_418053.5 | BTG2 | -6.66 | 2.29 | -0.70 |
| novel_mir975 | NM_204274.1 | CTGF | -1.14 | 1.36 | -0.70 |
| novel_mir975 | XM_415813.5 | SEBOX | -1.14 | 1.54 | -0.70 |
| novel_mir261 | XM_430279.5 | LOC425623 | 2.31 | -1.37 | -0.70 |
| novel_mir247 | NM_205422.1 | LCN8 | -1.37 | 1.15 | -0.70 |
| novel_mir304 | XM_015282088.1 | MAP3K8 | -6.29 | 3.09 | -0.70 |
| novel_mir881 | XM_003640342.3 | PIM3 | -1.02 | 1.25 | -0.70 |
| novel_mir692 | XM_004937677.2 | GPRC5A | -6.66 | 1.85 | -0.69 |
| novel_mir271 | XM_015290490.1 | IER5 | -6.57 | 1.51 | -0.69 |
| novel_mir1143 | NM_001012561.1 | MYLIP | 2.42 | -1.37 | -0.69 |
| novel_mir881 | XM_420888.3 | EGR4 | -1.02 | 5.62 | -0.69 |
| novel_mir304 | XM_015273822.1 | LOC107050017 | -6.29 | 3.53 | -0.69 |
| novel_mir261 | XM_423102.4 | PPP1R3C | 2.31 | -2.21 | -0.68 |
| novel_mir699 | NM_001101037.1 | CENPA | -1.65 | 1.17 | -0.68 |
| novel_mir720 | NM_001044636.1 | RASD1 | -1.09 | 1.08 | -0.68 |
| gga-miR-7475-5p | XM_420586.5 | PPP1R3B | 3.15 | -1.83 | -0.68 |
| novel_mir629 | XM_015290490.1 | IER5 | -6.57 | 1.51 | -0.68 |
| novel_mir262 | XM_015287598.1 | ZFP36L1 | -1.80 | 2.21 | -0.67 |
| novel_mir735 | XM_015290490.1 | IER5 | -1.85 | 1.51 | -0.67 |
| novel_mir975 | NM_001031537.1 | AREG | -1.14 | 1.73 | -0.67 |
| novel_mir822 | XM_015294014.1 | IRF1 | -1.35 | 3.32 | -0.67 |
| novel_mir304 | NM_205253.2 | CEBPB | -6.29 | 1.54 | -0.67 |
| novel_mir822 | XM_015273822.1 | LOC107050017 | -1.35 | 3.53 | -0.67 |
| novel_mir629 | XM_015302340.1 | LOC107057304 | -6.57 | 2.42 | -0.67 |
| novel_mir629 | XM_003640342.3 | PIM3 | -6.57 | 1.25 | -0.67 |
| novel_mir262 | XM_004938532.2 | ADPRHL1 | -1.80 | 2.44 | -0.67 |
| novel_mir205 | XM_015282088.1 | MAP3K8 | -1.77 | 3.09 | -0.67 |
| novel_mir666 | XM_015300578.1 | LOC426820 | -1.93 | 1.97 | -0.67 |
| novel_mir822 | NM_001137648.1 | SOCS1 | -1.35 | 1.67 | -0.67 |
| novel_mir508 | NM_001031037.1 | SERTAD2 | -1.43 | 1.26 | -0.66 |
| novel_mir82 | XM_420888.3 | EGR4 | -1.74 | 5.62 | -0.66 |
| novel_mir264 | XM_004938821.2 | ZC3H12C | -6.18 | 1.73 | -0.66 |
| novel_mir304 | XM_015282405.1 | NR4A3 | -6.29 | 2.58 | -0.66 |
| novel_mir495 | NM_001293287.1 | KCNJ8 | -4.51 | 2.38 | -0.66 |
| novel_mir205 | XM_015300220.1 | LOC107055390 | -1.77 | 4.05 | -0.66 |
| novel_mir304 | XM_015283145.1 | TRIB1 | -6.29 | 2.75 | -0.66 |
| novel_mir205 | XM_421754.5 | DUSP5 | -1.77 | 3.00 | -0.66 |
| novel_mir304 | NM_204642.1 | CBX4 | -6.29 | 1.96 | -0.66 |
| novel_mir247 | XM_004947403.2 | TMEM240 | -1.37 | 1.18 | -0.66 |
| novel_mir546 | XM_015277070.1 | LOC100857983 | -3.06 | 2.71 | -0.65 |
| novel_mir624 | XM_015300114.1 | CILP2 | -5.85 | 1.24 | -0.65 |
| novel_mir304 | NM_001031537.1 | AREG | -6.29 | 1.73 | -0.65 |
| novel_mir333 | XM_015293536.1 | DND1 | -1.51 | 1.69 | -0.65 |
| novel_mir881 | NM_001031037.1 | SERTAD2 | -1.02 | 1.26 | -0.65 |
| novel_mir569 | NM_205416.1 | IRF8 | -1.27 | 2.07 | -0.65 |
| novel_mir1240 | NM_204136.2 | EGR1 | -5.51 | 3.10 | -0.65 |
| novel_mir271 | NM_001044636.1 | RASD1 | -6.57 | 1.08 | -0.65 |
| novel_mir881 | NM_001278133.1 | CREM | -1.02 | 2.11 | -0.65 |
| novel_mir205 | NM_001278133.1 | CREM | -1.77 | 2.11 | -0.65 |
| novel_mir333 | XM_015297450.1 | ARID5A | -1.51 | 2.46 | -0.65 |
| novel_mir629 | NM_001317736.1 | TMEM136-1 | -6.57 | 1.50 | -0.65 |
| novel_mir205 | XM_015290968.1 | PFKFB3 | -1.77 | 2.55 | -0.65 |
| novel_mir82 | NM_204519.1 | P2RX1 | -1.74 | 3.28 | -0.65 |
| novel_mir333 | XM_015281780.1 | LOC107049140 | -1.51 | 1.32 | -0.64 |
| novel_mir146 | NM_001317736.1 | TMEM136-1 | -1.58 | 1.50 | -0.64 |
| novel_mir874 | NM_204540.1 | SOCS2 | -5.37 | 1.93 | -0.64 |
| novel_mir881 | XM_421754.5 | DUSP5 | -1.02 | 3.00 | -0.64 |
| novel_mir356 | NM_001030893.2 | NR0B2 | -5.44 | 1.49 | -0.64 |
| novel_mir304 | XM_418054.5 | PRELP | -6.29 | 5.46 | -0.64 |
| novel_mir237 | XM_015282088.1 | MAP3K8 | -6.51 | 3.09 | -0.64 |
| novel_mir330 | XM_015282086.1 | MAP3K8 | -1.23 | 3.09 | -0.64 |
| novel_mir237 | NM_001031537.1 | AREG | -6.51 | 1.73 | -0.64 |
| novel_mir975 | XM_015290275.1 | RGS16 | -1.14 | 4.10 | -0.64 |
| novel_mir304 | XM_015302587.1 | LOC107057513 | -6.29 | 2.31 | -0.63 |
| novel_mir237 | XM_015290911.1 | PFKFB3 | -6.51 | 2.55 | -0.63 |
| novel_mir205 | XM_015273822.1 | LOC107050017 | -1.77 | 3.53 | -0.63 |
| novel_mir262 | XM_015282942.1 | LOC107052763 | -1.80 | 1.07 | -0.63 |
| novel_mir1018 | XM_417765.5 | ZC3H12A | -2.05 | 1.42 | -0.63 |
| novel_mir1084 | XM_015302340.1 | LOC107057304 | -1.15 | 2.42 | -0.63 |
| gga-miR-12229-5p | XM_004936517.2 | FRMD6 | -1.09 | 1.12 | -0.63 |
| novel_mir205 | NM_205253.2 | CEBPB | -1.77 | 1.54 | -0.63 |
| novel_mir46 | XM_015277070.1 | LOC100857983 | -1.81 | 2.71 | -0.63 |
| novel_mir82 | XM_015297790.1 | EVA1B | -1.74 | 1.15 | -0.63 |
| novel_mir264 | XM_418054.5 | PRELP | -6.18 | 5.46 | -0.63 |
| novel_mir264 | XM_015291573.1 | CYP2J6L3 | -6.18 | 1.95 | -0.63 |
| novel_mir874 | XM_015281780.1 | LOC107049140 | -5.37 | 1.32 | -0.63 |
| novel_mir642 | NM_001277544.1 | RGS16 | -2.01 | 4.10 | -0.63 |
| novel_mir822 | XM_015282088.1 | MAP3K8 | -1.35 | 3.09 | -0.62 |
| novel_mir205 | NM_001031537.1 | AREG | -1.77 | 1.73 | -0.62 |
| novel_mir601 | XM_004937677.2 | GPRC5A | -5.63 | 1.85 | -0.62 |
| novel_mir237 | XM_015300220.1 | LOC107055390 | -6.51 | 4.05 | -0.62 |
| novel_mir203 | XM_004935303.2 | DISP1 | -6.09 | 1.37 | -0.62 |
| novel_mir975 | XM_015290201.1 | NR4A2 | -1.14 | 2.49 | -0.62 |
| novel_mir601 | NM_204274.1 | CTGF | -5.63 | 1.36 | -0.62 |
| novel_mir945 | XM_418053.5 | BTG2 | -1.92 | 2.29 | -0.62 |
| novel_mir82 | XM_015279916.1 | RHBDL3 | -1.74 | 1.68 | -0.62 |
| novel_mir205 | XM_003640342.3 | PIM3 | -1.77 | 1.25 | -0.62 |
| novel_mir955 | XM_015300220.1 | LOC107055390 | -1.12 | 4.05 | -0.62 |
| novel_mir914 | XM_015297790.1 | EVA1B | -5.69 | 1.15 | -0.62 |
| novel_mir264 | XM_418566.5 | FZD8 | -6.18 | 1.46 | -0.62 |
| novel_mir304 | XM_015282942.1 | LOC107052763 | -6.29 | 1.07 | -0.62 |
| novel_mir987 | NM_001293287.1 | KCNJ8 | -1.71 | 2.38 | -0.62 |
| novel_mir264 | XM_015299654.1 | TNS4 | -6.18 | 1.23 | -0.62 |
| novel_mir569 | NM_001044636.1 | RASD1 | -1.27 | 1.08 | -0.62 |
| novel_mir496 | XM_004946108.2 | LOC101747455 | 4.54 | -1.06 | -0.62 |
| novel_mir333 | XM_015283837.1 | ZFP36L2 | -1.51 | 1.38 | -0.62 |
| novel_mir412 | XM_015282155.1 | PTF1A | -1.69 | 1.34 | -0.62 |
| novel_mir271 | XM_003642712.3 | PIM1 | -6.57 | 1.57 | -0.62 |
| novel_mir205 | XM_015283145.1 | TRIB1 | -1.77 | 2.75 | -0.61 |
| novel_mir237 | XM_015282405.1 | NR4A3 | -6.51 | 2.58 | -0.61 |
| novel_mir261 | XM_004934986.2 | TRAF3IP3 | 2.31 | -2.14 | -0.61 |
| novel_mir330 | XM_015282942.1 | LOC107052763 | -1.23 | 1.07 | -0.61 |
| novel_mir330 | XM_015283145.1 | TRIB1 | -1.23 | 2.75 | -0.61 |
| novel_mir237 | XM_015273822.1 | LOC107050017 | -6.51 | 3.53 | -0.61 |
| novel_mir569 | XM_004936517.2 | FRMD6 | -1.27 | 1.12 | -0.61 |
| novel_mir818 | XM_015281184.1 | ZNF746 | 1.70 | -1.09 | -0.61 |
| gga-miR-12229-3p | XM_015300616.1 | LOC100859777 | -1.45 | 1.35 | -0.61 |
| gga-miR-205c-3p | XM_004937677.2 | GPRC5A | -1.05 | 1.85 | -0.61 |
| novel_mir237 | XM_421754.5 | DUSP5 | -6.51 | 3.00 | -0.61 |
| novel_mir624 | XM_416200.4 | GPRC5A | -5.85 | 1.85 | -0.61 |
| novel_mir1107 | XM_004937095.2 | LOC100857744 | 7.28 | -1.46 | -0.61 |
| novel_mir720 | XM_015291307.1 | ARTN | -1.09 | 7.00 | -0.61 |
| novel_mir788 | XM_015299654.1 | TNS4 | -6.13 | 1.23 | -0.61 |
| novel_mir202 | XM_015297790.1 | EVA1B | -4.63 | 1.15 | -0.61 |
| novel_mir518 | XM_015297790.1 | EVA1B | -4.63 | 1.15 | -0.61 |
| novel_mir304 | XM_015283837.1 | ZFP36L2 | -6.29 | 1.38 | -0.61 |
| novel_mir1128 | NM_001277837.1 | KCTD14 | 7.82 | -1.20 | -0.61 |
| novel_mir203 | XM_003640342.3 | PIM3 | -6.09 | 1.25 | -0.60 |
| novel_mir945 | XM_004936517.2 | FRMD6 | -1.92 | 1.12 | -0.60 |
| novel_mir624 | NM_205253.2 | CEBPB | -5.85 | 1.54 | -0.60 |
| novel_mir237 | NM_204642.1 | CBX4 | -6.51 | 1.96 | -0.60 |
| novel_mir955 | NM_205379.1 | TGIF1 | -1.12 | 1.20 | -0.60 |
| novel_mir820 | XM_015290911.1 | PFKFB3 | -6.44 | 2.55 | -0.60 |
| novel_mir796 | NM_204274.1 | CTGF | -5.57 | 1.36 | -0.60 |
| novel_mir140 | XM_004937095.2 | LOC100857744 | 5.41 | -1.46 | -0.60 |
| novel_mir796 | XM_004940646.2 | LOC101748744 | -5.57 | 1.59 | -0.60 |
| novel_mir304 | XM_418566.5 | FZD8 | -6.29 | 1.46 | -0.60 |
| novel_mir955 | XM_421754.5 | DUSP5 | -1.12 | 3.00 | -0.60 |
