# Supplementary material for: Interactions Among Expressed MicroRNAs and mRNAs in the Early Stages of Fowl Adenovirus Aerotype 4-Infected Leghorn Male Hepatocellular Cells
Source: Front Microbiol. 2020 May 19;11:831. doi: 10.3389/fmicb.2020.00831 (PMC7248314; doi:10.3389/fmicb.2020.00831)
Supplement: FIGURE S1 — Hierarchical clustering analysis of 784 differentially expressed miRNAs at three time points. The color in the heat map represents the gene expression changes. Red indicates the upregulation of gene expression, blue indicates the downregulation of expression, the darker color indicates a notable degree of differential gene expression, and white indicates no activity. [file Data_Sheet_1.ZIP › supplementary files/Table S9.doc]

TABLE S9 The negative miRNA-mRNA pairs between mock and infected LMHs at 120 min.

| **miRNA id** | **Target id** | **Symbol** | **miRNA Fold change** | **Target Fold change** | **Correlation (pearson)** |
| --- | --- | --- | --- | --- | --- |
| gga-miR-128-2-5p | XM_015297790.1 | EVA1B | -1.10 | 1.40 | -0.94 |
| novel_mir909 | XM_004938920.2 | CREBZF | 3.28 | -1.07 | -0.90 |
| novel_mir326 | XM_004947281.2 | HELZ2 | -1.08 | 1.04 | -0.89 |
| novel_mir1107 | NM_001305385.1 | HIST1H103 | 8.77 | -1.10 | -0.88 |
| gga-miR-7475-5p | NM_001305385.1 | HIST1H103 | 4.05 | -1.10 | -0.88 |
| novel_mir636 | XM_015283933.1 | LOC107052968 | -1.02 | 2.03 | -0.87 |
| novel_mir142 | XM_015283234.1 | DENND3 | -2.10 | 1.20 | -0.86 |
| novel_mir326 | XM_004935937.2 | EVA1A | -1.08 | 1.28 | -0.86 |
| novel_mir1117 | XM_004945812.2 | SLC25A25 | -1.82 | 1.27 | -0.86 |
| novel_mir1128 | XM_015278478.1 | ZIC3 | 8.06 | -1.17 | -0.85 |
| gga-miR-128-2-5p | XM_015277319.1 | PTGER4 | -1.10 | 1.38 | -0.85 |
| novel_mir655 | XM_015299419.1 | LOC107055280 | -5.42 | 1.18 | -0.85 |
| gga-miR-128-2-5p | XM_015291556.1 | LOC101747338 | -1.10 | 1.29 | -0.84 |
| novel_mir326 | XM_015287724.1 | ISM2 | -1.08 | 1.79 | -0.84 |
| gga-miR-128-2-5p | XM_015277004.1 | IRS1 | -1.10 | 1.03 | -0.84 |
| novel_mir1107 | XM_015299133.1 | MTCH1 | 8.77 | -1.02 | -0.83 |
| novel_mir326 | NM_205493.1 | GLUL | -1.08 | 1.43 | -0.83 |
| novel_mir636 | NM_001079726.1 | LOC420419 | -1.02 | 1.20 | -0.83 |
| gga-miR-128-2-5p | XM_425376.5 | EVPL | -1.10 | 2.90 | -0.83 |
| novel_mir509 | XM_417765.5 | ZC3H12A | -1.35 | 1.76 | -0.82 |
| novel_mir276 | NM_001031037.1 | SERTAD2 | -1.68 | 1.12 | -0.82 |
| novel_mir142 | XM_015286842.1 | LOC101751752 | -2.10 | 2.73 | -0.82 |
| novel_mir142 | NM_205043.1 | PADI3 | -2.10 | 1.36 | -0.82 |
| novel_mir636 | XM_004947965.2 | KCNJ5 | -1.02 | 1.12 | -0.82 |
| gga-miR-128-2-5p | XM_015291070.1 | PTCH2 | -1.10 | 1.56 | -0.82 |
| novel_mir636 | XM_015273318.1 | LOC107049603 | -1.02 | 4.36 | -0.82 |
| novel_mir142 | XM_001232082.4 | FAM102B | -2.10 | 2.20 | -0.82 |
| novel_mir1107 | XM_015279809.1 | CARD9 | 8.77 | -1.03 | -0.82 |
| novel_mir909 | NM_001305385.1 | HIST1H103 | 3.28 | -1.10 | -0.82 |
| gga-miR-128-2-5p | XM_015290492.1 | LHX4 | -1.10 | 1.35 | -0.82 |
| novel_mir142 | XM_015294739.1 | LOC107054654 | -2.10 | 5.69 | -0.82 |
| novel_mir344 | XM_004935096.2 | NFATC1 | -1.28 | 1.01 | -0.82 |
| novel_mir1107 | XM_003641535.3 | LOC100857573 | 8.77 | -1.90 | -0.82 |
| novel_mir636 | XM_015293755.1 | GRIA1 | -1.02 | 1.17 | -0.81 |
| gga-miR-128-2-5p | XM_003641641.3 | OBSL1 | -1.10 | 1.69 | -0.81 |
| novel_mir636 | XM_015273184.1 | EGR3 | -1.02 | 4.18 | -0.81 |
| novel_mir1117 | XM_015287724.1 | ISM2 | -1.82 | 1.79 | -0.81 |
| gga-miR-7475-5p | XM_015276359.1 | NDST3 | 4.05 | -1.20 | -0.81 |
| novel_mir509 | NM_205298.2 | ETS1 | -1.35 | 1.66 | -0.81 |
| novel_mir261 | XM_004948741.2 | ATCAY | 2.94 | -1.25 | -0.81 |
| gga-miR-7475-5p | XM_015294089.1 | LOC771537 | 4.05 | -1.46 | -0.81 |
| gga-miR-128-2-5p | XM_015277882.1 | LOC101749060 | -1.10 | 1.55 | -0.81 |
| novel_mir142 | XM_015272984.1 | NHS | -2.10 | 1.70 | -0.81 |
| novel_mir1117 | XM_015283874.1 | ATF3 | -1.82 | 1.27 | -0.81 |
| gga-miR-128-2-5p | NM_205206.1 | INHBB | -1.10 | 1.22 | -0.81 |
| novel_mir326 | XM_015277651.1 | ENC1 | -1.08 | 1.07 | -0.81 |
| novel_mir636 | XM_015273518.1 | LOC100857380 | -1.02 | 3.92 | -0.81 |
| gga-miR-128-2-5p | XM_015276044.1 | MYCN | -1.10 | 1.09 | -0.80 |
| novel_mir509 | NM_001033641.1 | TNFRSF11B | -1.35 | 1.37 | -0.80 |
| novel_mir326 | XM_417497.5 | TFAP2C | -1.08 | 1.24 | -0.80 |
| novel_mir326 | XM_003641084.3 | IRS4 | -1.08 | 1.24 | -0.80 |
| novel_mir683 | XM_426034.4 | NHLRC1 | 7.90 | -1.23 | -0.80 |
| novel_mir326 | XM_015290201.1 | NR4A2 | -1.08 | 3.19 | -0.80 |
| novel_mir326 | NM_001037275.2 | TRIM71 | -1.08 | 1.72 | -0.80 |
| novel_mir120 | XM_015283427.1 | BCL2L11 | 3.10 | -1.14 | -0.80 |
| novel_mir261 | XM_015278478.1 | ZIC3 | 2.94 | -1.17 | -0.80 |
| novel_mir1117 | XM_015283529.1 | JAG1 | -1.82 | 1.59 | -0.80 |
| novel_mir847 | XM_015300578.1 | LOC426820 | -1.75 | 2.10 | -0.80 |
| novel_mir326 | XM_425541.5 | ADAMTS5 | -1.08 | 2.10 | -0.80 |
| novel_mir1117 | XM_015283145.1 | TRIB1 | -1.82 | 3.05 | -0.80 |
| novel_mir1117 | XM_015299177.1 | ADAMTS1 | -1.82 | 3.64 | -0.80 |
| novel_mir326 | NM_001199453.1 | THBS1 | -1.08 | 1.74 | -0.80 |
| novel_mir636 | XM_015280288.1 | ABHD17B | -1.02 | 1.11 | -0.80 |
| novel_mir683 | XM_015281606.1 | SKIDA1 | 7.90 | -1.12 | -0.80 |
| novel_mir326 | XM_015299148.1 | SLC6A17 | -1.08 | 1.45 | -0.79 |
| novel_mir326 | NM_001030779.1 | TNFRSF1A | -1.08 | 1.52 | -0.79 |
| gga-miR-128-2-5p | XM_015301388.1 | LOC107056355 | -1.10 | 4.23 | -0.79 |
| novel_mir636 | XM_425376.5 | EVPL | -1.02 | 2.90 | -0.79 |
| novel_mir326 | XM_015291453.1 | LOC424917 | -1.08 | 1.15 | -0.79 |
| novel_mir509 | XM_015291070.1 | PTCH2 | -1.35 | 1.56 | -0.79 |
| gga-miR-128-2-5p | NM_204757.1 | MAFF | -1.10 | 1.20 | -0.79 |
| gga-miR-128-2-5p | NM_204680.1 | ZBTB7A | -1.10 | 1.20 | -0.79 |
| gga-miR-128-2-5p | XM_015273318.1 | LOC107049603 | -1.10 | 4.36 | -0.79 |
| gga-miR-128-2-5p | XM_015293935.1 | MAML1 | -1.10 | 1.05 | -0.79 |
| novel_mir142 | XM_004945037.2 | SPRY4 | -2.10 | 1.69 | -0.79 |
| novel_mir636 | XM_003641038.3 | PRDM1 | -1.02 | 1.32 | -0.79 |
| novel_mir636 | XM_015272640.1 | CLCF1 | -1.02 | 1.89 | -0.79 |
| gga-miR-128-2-5p | XM_015272640.1 | CLCF1 | -1.10 | 1.89 | -0.79 |
| gga-miR-128-2-5p | NM_205420.1 | GATA6 | -1.10 | 1.15 | -0.79 |
| novel_mir326 | NM_204316.3 | ADORA1 | -1.08 | 2.04 | -0.79 |
| novel_mir636 | XM_015299177.1 | ADAMTS1 | -1.02 | 3.64 | -0.79 |
| novel_mir272 | NM_001030765.1 | CHRM2 | -1.39 | 2.15 | -0.79 |
| gga-miR-128-2-5p | XM_015286678.1 | LOC101751827 | -1.10 | 1.57 | -0.79 |
| novel_mir683 | NM_204098.2 | FZD10 | 7.90 | -1.66 | -0.78 |
| novel_mir326 | XM_015290010.1 | ETV6 | -1.08 | 1.25 | -0.78 |
| novel_mir636 | XM_015291070.1 | PTCH2 | -1.02 | 1.56 | -0.78 |
| novel_mir636 | XM_015283145.1 | TRIB1 | -1.02 | 3.05 | -0.78 |
| novel_mir326 | XM_418838.4 | EEPD1 | -1.08 | 1.06 | -0.78 |
| novel_mir326 | NM_001012534.1 | TOB2 | -1.08 | 1.29 | -0.78 |
| gga-miR-128-2-5p | XM_015278663.1 | LOC100858332 | -1.10 | 1.58 | -0.78 |
| novel_mir458 | XM_015295904.1 | TRIM50 | -1.34 | 2.08 | -0.78 |
| novel_mir326 | XM_015295882.1 | NUFIP2 | -1.08 | 1.01 | -0.78 |
| novel_mir1117 | NM_001037275.2 | TRIM71 | -1.82 | 1.72 | -0.78 |
| novel_mir636 | NM_001031037.1 | SERTAD2 | -1.02 | 1.12 | -0.78 |
| gga-miR-6654-3p | XM_015291448.1 | MUC4 | -2.63 | 1.50 | -0.78 |
| novel_mir1117 | XM_003641641.3 | OBSL1 | -1.82 | 1.69 | -0.78 |
| novel_mir326 | NM_205212.2 | MET | -1.08 | 1.07 | -0.78 |
| novel_mir1117 | XM_418566.5 | FZD8 | -1.82 | 1.77 | -0.78 |
| novel_mir272 | XM_004947281.2 | HELZ2 | -1.39 | 1.04 | -0.78 |
| novel_mir509 | XM_003641901.3 | SLC9A5 | -1.35 | 1.03 | -0.78 |
| gga-miR-128-2-5p | XM_015290201.1 | NR4A2 | -1.10 | 3.19 | -0.77 |
| gga-miR-128-2-5p | XM_015273184.1 | EGR3 | -1.10 | 4.18 | -0.77 |
| novel_mir636 | NM_001122648.1 | DIO3 | -1.02 | 1.98 | -0.77 |
| novel_mir509 | XM_015285195.1 | LOC101752065 | -1.35 | 2.05 | -0.77 |
| novel_mir142 | XM_015298341.1 | RAB2B | -2.10 | 1.06 | -0.77 |
| novel_mir326 | XM_417013.5 | KLF5 | -1.08 | 1.93 | -0.77 |
| novel_mir198 | XM_015287724.1 | ISM2 | -5.65 | 1.79 | -0.77 |
| novel_mir636 | NM_204642.1 | CBX4 | -1.02 | 2.05 | -0.77 |
| novel_mir636 | XM_015299654.1 | TNS4 | -1.02 | 1.32 | -0.77 |
| novel_mir636 | NM_001031166.2 | TNIP2 | -1.02 | 1.20 | -0.77 |
| novel_mir458 | NM_001252254.1 | SLC26A6 | -1.34 | 2.50 | -0.77 |
| gga-miR-7475-5p | XM_015281606.1 | SKIDA1 | 4.05 | -1.12 | -0.77 |
| novel_mir509 | NM_001031012.1 | TUBB6 | -1.35 | 1.01 | -0.77 |
| novel_mir326 | NM_001037274.1 | WNT7B | -1.08 | 2.21 | -0.77 |
| novel_mir326 | NM_001257283.1 | MCL1 | -1.08 | 1.17 | -0.77 |
| novel_mir636 | NM_204891.1 | ATP2A3 | -1.02 | 1.93 | -0.77 |
| gga-miR-128-2-5p | NM_205498.1 | IL8L2 | -1.10 | 4.55 | -0.77 |
| novel_mir636 | NM_001008676.2 | IFNGR2 | -1.02 | 1.24 | -0.77 |
| novel_mir636 | XM_418054.5 | PRELP | -1.02 | 6.06 | -0.77 |
| novel_mir261 | XM_015280876.1 | AQP11 | 2.94 | -1.00 | -0.77 |
| novel_mir326 | NM_001012930.1 | BCL6 | -1.08 | 2.25 | -0.77 |
| novel_mir636 | NM_001031492.1 | PMEPA1 | -1.02 | 1.24 | -0.77 |
| novel_mir636 | NM_001083365.1 | PTGER2 | -1.02 | 1.11 | -0.77 |
| novel_mir326 | NM_001199549.1 | SLC38A4 | -1.08 | 1.05 | -0.77 |
| gga-miR-6654-3p | XM_015298695.1 | LOC101749375 | -2.63 | 1.04 | -0.77 |
| novel_mir636 | XM_015293658.1 | HTR4 | -1.02 | 1.45 | -0.76 |
| novel_mir1117 | XM_015297000.1 | ERRFI1 | -1.82 | 3.11 | -0.76 |
| gga-miR-6651-5p | XM_004937095.2 | LOC100857744 | 3.64 | -2.17 | -0.76 |
| gga-miR-128-2-5p | NM_001257283.1 | MCL1 | -1.10 | 1.17 | -0.76 |
| novel_mir261 | XM_015281598.1 | LOC107052502 | 2.94 | -1.11 | -0.76 |
| gga-miR-128-2-5p | NM_001012693.1 | MGAT3 | -1.10 | 1.17 | -0.76 |
| novel_mir636 | NM_001193295.1 | CSF1 | -1.02 | 1.32 | -0.76 |
| gga-miR-7475-5p | XM_004938920.2 | CREBZF | 4.05 | -1.07 | -0.76 |
| novel_mir326 | NM_001045836.1 | FOSL2 | -1.08 | 2.90 | -0.76 |
| novel_mir326 | XM_001231573.4 | CREBRF | -1.08 | 1.22 | -0.76 |
| novel_mir326 | XM_003642861.3 | SBNO2 | -1.08 | 1.21 | -0.76 |
| novel_mir509 | NM_001030779.1 | TNFRSF1A | -1.35 | 1.52 | -0.76 |
| novel_mir326 | XM_015283874.1 | ATF3 | -1.08 | 1.27 | -0.76 |
| novel_mir636 | NM_001037275.2 | TRIM71 | -1.02 | 1.72 | -0.76 |
| novel_mir142 | XM_003642861.3 | SBNO2 | -2.10 | 1.21 | -0.76 |
| novel_mir272 | XM_003641641.3 | OBSL1 | -1.39 | 1.69 | -0.76 |
| novel_mir326 | XM_015277882.1 | LOC101749060 | -1.08 | 1.55 | -0.76 |
| novel_mir636 | XM_015300401.1 | NAB2 | -1.02 | 1.83 | -0.76 |
| novel_mir142 | XM_015284930.1 | FAM110C | -2.10 | 1.39 | -0.76 |
| novel_mir898 | XM_015283874.1 | ATF3 | -6.45 | 1.27 | -0.76 |
| novel_mir142 | XM_418838.4 | EEPD1 | -2.10 | 1.06 | -0.76 |
| gga-miR-128-2-5p | NM_001171771.1 | APC2 | -1.10 | 1.49 | -0.76 |
| novel_mir1117 | XM_015284930.1 | FAM110C | -1.82 | 1.39 | -0.76 |
| novel_mir636 | XM_015291448.1 | MUC4 | -1.02 | 1.50 | -0.76 |
| novel_mir427 | XM_015292715.1 | CHST4 | -5.80 | 1.09 | -0.76 |
| novel_mir636 | XM_015283874.1 | ATF3 | -1.02 | 1.27 | -0.75 |
| novel_mir636 | XM_015283837.1 | ZFP36L2 | -1.02 | 1.75 | -0.75 |
| novel_mir636 | XM_417765.5 | ZC3H12A | -1.02 | 1.76 | -0.75 |
| novel_mir636 | XM_015286678.1 | LOC101751827 | -1.02 | 1.57 | -0.75 |
| novel_mir636 | XM_015297450.1 | ARID5A | -1.02 | 2.44 | -0.75 |
| gga-miR-128-2-5p | XM_004945812.2 | SLC25A25 | -1.10 | 1.27 | -0.75 |
| novel_mir1117 | XM_015290010.1 | ETV6 | -1.82 | 1.25 | -0.75 |
| novel_mir636 | XM_004947494.2 | KAZN | -1.02 | 1.54 | -0.75 |
| novel_mir636 | NM_205298.2 | ETS1 | -1.02 | 1.66 | -0.75 |
| novel_mir142 | NM_001079726.1 | LOC420419 | -2.10 | 1.20 | -0.75 |
| novel_mir326 | XM_015293007.1 | LOC395991 | -1.08 | 1.08 | -0.75 |
| novel_mir1117 | XM_015282406.1 | NR4A3 | -1.82 | 3.17 | -0.75 |
| novel_mir198 | XM_015299148.1 | SLC6A17 | -5.65 | 1.45 | -0.75 |
| novel_mir636 | NM_001317736.1 | TMEM136-1 | -1.02 | 1.63 | -0.75 |
| gga-miR-6654-3p | XM_004936919.2 | FOXE3 | -2.63 | 1.47 | -0.75 |
| novel_mir142 | XM_015277651.1 | ENC1 | -2.10 | 1.07 | -0.75 |
| novel_mir1117 | XM_425541.5 | ADAMTS5 | -1.82 | 2.10 | -0.75 |
| novel_mir304 | XM_003640342.3 | PIM3 | -1.68 | 1.07 | -0.75 |
| gga-miR-128-2-5p | XM_418566.5 | FZD8 | -1.10 | 1.77 | -0.75 |
| novel_mir272 | XM_015299150.1 | SLC6A17 | -1.39 | 1.45 | -0.75 |
| gga-miR-128-2-5p | XM_420218.4 | IL13RA1 | -1.10 | 1.29 | -0.75 |
| novel_mir326 | XM_015290492.1 | LHX4 | -1.08 | 1.35 | -0.75 |
| novel_mir326 | NM_001012693.1 | MGAT3 | -1.08 | 1.17 | -0.75 |
| novel_mir261 | XM_003641535.3 | LOC100857573 | 2.94 | -1.90 | -0.75 |
| novel_mir1117 | XM_015300401.1 | NAB2 | -1.82 | 1.83 | -0.75 |
| novel_mir272 | XM_015275647.1 | ULK1 | -1.39 | 1.34 | -0.75 |
| novel_mir304 | XM_015280288.1 | ABHD17B | -1.68 | 1.11 | -0.74 |
| novel_mir142 | XM_425541.5 | ADAMTS5 | -2.10 | 2.10 | -0.74 |
| novel_mir326 | XM_001231361.4 | ASB10 | -1.08 | 2.19 | -0.74 |
| novel_mir326 | XM_418054.5 | PRELP | -1.08 | 6.06 | -0.74 |
| gga-miR-7475-5p | XM_015278478.1 | ZIC3 | 4.05 | -1.17 | -0.74 |
| novel_mir226 | NM_001252254.1 | SLC26A6 | -1.13 | 2.50 | -0.74 |
| novel_mir636 | NM_204731.1 | SOX8 | -1.02 | 2.23 | -0.74 |
| novel_mir509 | NM_001012534.1 | TOB2 | -1.35 | 1.29 | -0.74 |
| novel_mir636 | XM_015290492.1 | LHX4 | -1.02 | 1.35 | -0.74 |
| novel_mir636 | XM_015291556.1 | LOC101747338 | -1.02 | 1.29 | -0.74 |
| novel_mir120 | XM_004938920.2 | CREBZF | 3.10 | -1.07 | -0.74 |
| novel_mir142 | XM_015279230.1 | MLKL | -2.10 | 1.78 | -0.74 |
| gga-miR-6654-3p | XM_015286577.1 | TPH1 | -2.63 | 1.61 | -0.74 |
| novel_mir636 | XM_015276814.1 | EPHA4 | -1.02 | 2.17 | -0.74 |
| gga-miR-7475-5p | NM_204948.1 | LFNG | 4.05 | -1.60 | -0.74 |
| novel_mir509 | XM_015279209.1 | MET | -1.35 | 1.07 | -0.73 |
| novel_mir636 | NM_001257283.1 | MCL1 | -1.02 | 1.17 | -0.73 |
| novel_mir509 | XM_015295905.1 | FKBP6 | -1.35 | 1.28 | -0.73 |
| novel_mir326 | XM_015296101.1 | SSH2 | -1.08 | 1.15 | -0.73 |
| gga-miR-128-2-5p | XM_015286842.1 | LOC101751752 | -1.10 | 2.73 | -0.73 |
| novel_mir636 | XM_015286894.1 | LOC107049198 | -1.02 | 2.50 | -0.73 |
| novel_mir1117 | XM_421754.5 | DUSP5 | -1.82 | 2.88 | -0.73 |
| novel_mir304 | XM_421754.5 | DUSP5 | -1.68 | 2.88 | -0.73 |
| novel_mir326 | NM_001199504.1 | PHYHIPL | -1.08 | 1.04 | -0.73 |
| novel_mir636 | XM_015277004.1 | IRS1 | -1.02 | 1.03 | -0.73 |
| novel_mir636 | XM_015282942.1 | LOC107052763 | -1.02 | 1.01 | -0.73 |
| novel_mir198 | NM_001001530.1 | LEPREL1 | -5.65 | 1.01 | -0.73 |
| novel_mir1117 | NM_205206.1 | INHBB | -1.82 | 1.22 | -0.73 |
| novel_mir326 | XM_015289308.1 | KANSL1L | -1.08 | 1.85 | -0.73 |
| novel_mir509 | XM_003642861.3 | SBNO2 | -1.35 | 1.21 | -0.73 |
| gga-miR-12245-3p | XM_015293773.1 | FAXDC2 | 2.23 | -1.05 | -0.73 |
| novel_mir142 | XM_015300161.1 | LOC101748386 | -2.10 | 1.85 | -0.73 |
| novel_mir509 | XM_015277883.1 | LOC101749060 | -1.35 | 1.55 | -0.73 |
| novel_mir636 | XM_015300578.1 | LOC426820 | -1.02 | 2.10 | -0.73 |
| novel_mir1045 | NM_204589.1 | ID3 | 5.50 | -1.47 | -0.73 |
| gga-miR-128-2-5p | NM_001317736.1 | TMEM136-1 | -1.10 | 1.63 | -0.73 |
| novel_mir1107 | XM_015294067.1 | SHROOM1 | 8.77 | -1.03 | -0.73 |
| novel_mir636 | NM_001171771.1 | APC2 | -1.02 | 1.49 | -0.73 |
| novel_mir636 | XM_004939652.2 | HIVEP1 | -1.02 | 1.33 | -0.73 |
| novel_mir458 | XM_015285528.1 | LNX1 | -1.34 | 1.37 | -0.73 |
| gga-miR-128-2-5p | XM_015286894.1 | LOC107049198 | -1.10 | 2.50 | -0.73 |
| gga-miR-7475-5p | XM_426034.4 | NHLRC1 | 4.05 | -1.23 | -0.73 |
| novel_mir142 | XM_015296568.1 | LOC107054848 | -2.10 | 1.80 | -0.73 |
| novel_mir142 | NM_001031356.1 | PLEKHO1 | -2.10 | 1.93 | -0.73 |
| novel_mir120 | XM_004947488.2 | FAM43B | 3.10 | -1.20 | -0.73 |
| novel_mir326 | XM_015296040.1 | SERPINF2 | -1.08 | 1.12 | -0.73 |
| novel_mir326 | NM_204928.1 | MNX1 | -1.08 | 1.72 | -0.73 |
| novel_mir636 | XM_015297790.1 | EVA1B | -1.02 | 1.40 | -0.72 |
| gga-miR-7475-5p | XM_004948609.2 | LOC420030 | 4.05 | -1.28 | -0.72 |
| novel_mir509 | XM_015296101.1 | SSH2 | -1.35 | 1.15 | -0.72 |
| novel_mir142 | XM_424739.5 | PLK2 | -2.10 | 1.27 | -0.72 |
| novel_mir636 | XM_422425.5 | PLK3 | -1.02 | 1.63 | -0.72 |
| novel_mir636 | NM_001012760.1 | TSC22D1 | -1.02 | 1.32 | -0.72 |
| gga-miR-6654-3p | NM_001030648.1 | NET1 | -2.63 | 1.07 | -0.72 |
| novel_mir636 | XM_425541.5 | ADAMTS5 | -1.02 | 2.10 | -0.72 |
| gga-miR-6654-3p | XM_015283837.1 | ZFP36L2 | -2.63 | 1.75 | -0.72 |
| novel_mir326 | XM_015299978.1 | MIDN | -1.08 | 1.30 | -0.72 |
| novel_mir636 | XM_015291453.1 | LOC424917 | -1.02 | 1.15 | -0.72 |
| gga-miR-128-2-5p | XM_015299978.1 | MIDN | -1.10 | 1.30 | -0.72 |
| novel_mir198 | XM_015277692.1 | IL1R1 | -5.65 | 1.81 | -0.72 |
| gga-miR-6654-3p | XM_421754.5 | DUSP5 | -2.63 | 2.88 | -0.72 |
| novel_mir1051 | XM_003640916.3 | SYNJ2 | -4.70 | 1.12 | -0.72 |
| novel_mir636 | NM_001171593.1 | WNT7B | -1.02 | 2.21 | -0.72 |
| novel_mir326 | NM_204354.1 | DUSP6 | -1.08 | 1.88 | -0.72 |
| novel_mir272 | XM_015296101.1 | SSH2 | -1.39 | 1.15 | -0.72 |
| novel_mir142 | XM_003642734.3 | PHLDA3 | -2.10 | 1.84 | -0.72 |
| novel_mir272 | XM_015294500.1 | SCNN1G | -1.39 | 1.04 | -0.72 |
| novel_mir796 | NM_204681.2 | MGAT4C | -5.42 | 1.02 | -0.71 |
| novel_mir909 | XM_004939681.2 | LOC101749447 | 3.28 | -1.16 | -0.71 |
| novel_mir400 | XM_003642304.3 | ADAMTSL2 | -4.70 | 1.11 | -0.71 |
| novel_mir304 | XM_015300220.1 | LOC107055390 | -1.68 | 3.42 | -0.71 |
| novel_mir326 | NM_205420.1 | GATA6 | -1.08 | 1.15 | -0.71 |
| gga-miR-7475-5p | NM_001039604.1 | NOG2 | 4.05 | -4.00 | -0.71 |
| gga-miR-128-2-5p | XM_015282955.1 | OSR2 | -1.10 | 1.05 | -0.71 |
| novel_mir1128 | XM_004948741.2 | ATCAY | 8.06 | -1.25 | -0.71 |
| novel_mir142 | XM_015297000.1 | ERRFI1 | -2.10 | 3.11 | -0.71 |
| novel_mir209 | XM_015291448.1 | MUC4 | -1.23 | 1.50 | -0.71 |
| gga-miR-128-2-5p | XM_015283837.1 | ZFP36L2 | -1.10 | 1.75 | -0.71 |
| novel_mir142 | XM_015300401.1 | NAB2 | -2.10 | 1.83 | -0.71 |
| novel_mir326 | XM_015297991.1 | TMEM136-1 | -1.08 | 1.63 | -0.71 |
| novel_mir1107 | XM_015302397.1 | LOC101749510 | 8.77 | -1.98 | -0.71 |
| novel_mir326 | XM_015286842.1 | LOC101751752 | -1.08 | 2.73 | -0.71 |
| novel_mir120 | XM_426034.4 | NHLRC1 | 3.10 | -1.23 | -0.71 |
| gga-miR-6654-3p | XM_001234813.4 | EPHA2 | -2.63 | 2.29 | -0.70 |
| novel_mir142 | XM_425376.5 | EVPL | -2.10 | 2.90 | -0.70 |
| novel_mir636 | NM_205206.1 | INHBB | -1.02 | 1.22 | -0.70 |
| novel_mir142 | XM_015277935.1 | GATA6 | -2.10 | 1.15 | -0.70 |
| novel_mir326 | XM_004936919.2 | FOXE3 | -1.08 | 1.47 | -0.70 |
| novel_mir636 | XM_015296994.1 | PADI2 | -1.02 | 1.58 | -0.70 |
| novel_mir1117 | XM_015298873.1 | CSF1 | -1.82 | 1.32 | -0.70 |
| novel_mir209 | NM_206993.1 | DMP1 | -1.23 | 2.72 | -0.70 |
| novel_mir636 | NM_205268.1 | NOV | -1.02 | 2.10 | -0.70 |
| novel_mir509 | XM_004947281.2 | HELZ2 | -1.35 | 1.04 | -0.70 |
| novel_mir544 | NM_001079726.1 | LOC420419 | -6.14 | 1.20 | -0.70 |
| novel_mir272 | XM_015293536.1 | DND1 | -1.39 | 1.65 | -0.70 |
| novel_mir847 | XM_015293536.1 | DND1 | -1.75 | 1.65 | -0.70 |
| novel_mir261 | XM_430279.5 | LOC425623 | 2.94 | -1.29 | -0.70 |
| gga-miR-128-2-5p | XM_004934900.2 | REN | -1.10 | 1.44 | -0.70 |
| novel_mir137 | XM_015273034.1 | TUT1 | -1.76 | 1.24 | -0.70 |
| novel_mir304 | XM_015282088.1 | MAP3K8 | -1.68 | 2.50 | -0.70 |
| novel_mir1117 | NM_205237.3 | BMP4 | -1.82 | 1.66 | -0.70 |
| novel_mir1117 | NM_001001530.1 | LEPREL1 | -1.82 | 1.01 | -0.70 |
| novel_mir142 | XM_015280026.1 | LOC101749610 | -2.10 | 1.16 | -0.70 |
| novel_mir326 | NM_204484.2 | SOX4 | -1.08 | 1.09 | -0.70 |
| gga-miR-6654-3p | XM_003640342.3 | PIM3 | -2.63 | 1.07 | -0.70 |
| novel_mir400 | XM_015299148.1 | SLC6A17 | -4.70 | 1.45 | -0.70 |
| novel_mir261 | XM_004946472.2 | COL26A1 | 2.94 | -1.04 | -0.69 |
| gga-miR-6654-3p | XM_015298341.1 | RAB2B | -2.63 | 1.06 | -0.69 |
| novel_mir261 | XM_015281606.1 | SKIDA1 | 2.94 | -1.12 | -0.69 |
| novel_mir1143 | XM_015302160.1 | LOC107057134 | 2.07 | -1.59 | -0.69 |
| gga-miR-6654-3p | NM_204535.4 | SPP1 | -2.63 | 3.44 | -0.69 |
| novel_mir683 | XM_004941244.2 | NDST3 | 7.90 | -1.20 | -0.69 |
| novel_mir1117 | NM_001031356.1 | PLEKHO1 | -1.82 | 1.93 | -0.69 |
| novel_mir272 | XM_004940080.2 | RHPN1 | -1.39 | 1.11 | -0.69 |
| novel_mir272 | NM_001012534.1 | TOB2 | -1.39 | 1.29 | -0.69 |
| novel_mir667 | XM_015286661.1 | HPS5 | -1.86 | 1.53 | -0.69 |
| gga-miR-6654-3p | XM_417765.5 | ZC3H12A | -2.63 | 1.76 | -0.69 |
| novel_mir898 | NM_001085359.1 | DUSP1 | -6.45 | 1.53 | -0.69 |
| novel_mir636 | XM_001232892.3 | DUSP8 | -1.02 | 1.23 | -0.69 |
| gga-miR-128-2-5p | XM_015298873.1 | CSF1 | -1.10 | 1.32 | -0.69 |
| gga-miR-6654-3p | NM_001033641.1 | TNFRSF11B | -2.63 | 1.37 | -0.69 |
| gga-miR-6654-3p | XM_001233590.3 | LOC770260 | -2.63 | 1.18 | -0.69 |
| gga-miR-6654-3p | XM_015291070.1 | PTCH2 | -2.63 | 1.56 | -0.69 |
| novel_mir133 | NM_001079726.1 | LOC420419 | -4.90 | 1.20 | -0.69 |
| novel_mir142 | NM_001199415.1 | PRRG4 | -2.10 | 2.78 | -0.69 |
| novel_mir1256 | NM_204891.1 | ATP2A3 | -5.48 | 1.93 | -0.69 |
| gga-miR-6654-3p | XM_003641038.3 | PRDM1 | -2.63 | 1.32 | -0.69 |
| novel_mir344 | XM_015286661.1 | HPS5 | -1.28 | 1.53 | -0.69 |
| novel_mir142 | XM_001234813.4 | EPHA2 | -2.10 | 2.29 | -0.69 |
| gga-miR-7475-5p | XM_015302015.1 | LOC107057031 | 4.05 | -1.52 | -0.69 |
| novel_mir636 | NM_204358.1 | BMP2 | -1.02 | 1.70 | -0.69 |
| gga-miR-6654-3p | XM_015290849.1 | PFKFB3 | -2.63 | 2.51 | -0.69 |
| novel_mir261 | XM_004948609.2 | LOC420030 | 2.94 | -1.28 | -0.69 |
| novel_mir326 | XM_015282406.1 | NR4A3 | -1.08 | 3.17 | -0.69 |
| novel_mir142 | XM_015286678.1 | LOC101751827 | -2.10 | 1.57 | -0.69 |
| novel_mir326 | XM_004934565.2 | RIPK4 | -1.08 | 2.07 | -0.69 |
| gga-miR-128-2-5p | XM_015283933.1 | LOC107052968 | -1.10 | 2.03 | -0.69 |
| novel_mir932 | XM_015283837.1 | ZFP36L2 | -6.02 | 1.75 | -0.69 |
| novel_mir304 | XM_015273822.1 | LOC107050017 | -1.68 | 2.86 | -0.69 |
| novel_mir959 | XM_004939293.2 | EFCAB1 | 2.35 | -1.01 | -0.68 |
| novel_mir636 | XM_015280026.1 | LOC101749610 | -1.02 | 1.16 | -0.68 |
| novel_mir146 | XM_015296040.1 | SERPINF2 | -2.91 | 1.12 | -0.68 |
| novel_mir636 | XM_015285803.1 | CPEB2 | -1.02 | 1.18 | -0.68 |
| novel_mir198 | XM_015300114.1 | CILP2 | -5.65 | 1.80 | -0.68 |
| novel_mir326 | NM_001171771.1 | APC2 | -1.08 | 1.49 | -0.68 |
| novel_mir1117 | XM_015295905.1 | FKBP6 | -1.82 | 1.28 | -0.68 |
| novel_mir500 | XM_015291448.1 | MUC4 | -1.25 | 1.50 | -0.68 |
| novel_mir198 | XM_001234813.4 | EPHA2 | -5.65 | 2.29 | -0.68 |
| novel_mir770 | XM_003641409.3 | BCL11B | -6.60 | 2.33 | -0.68 |
| novel_mir636 | NM_204519.1 | P2RX1 | -1.02 | 3.32 | -0.68 |
| novel_mir142 | XM_004940080.2 | RHPN1 | -2.10 | 1.11 | -0.68 |
| novel_mir272 | XM_015299959.1 | LOC107055350 | -1.39 | 1.48 | -0.68 |
| novel_mir636 | XM_015287724.1 | ISM2 | -1.02 | 1.79 | -0.68 |
| novel_mir458 | XM_004944697.2 | CHL1 | -1.34 | 1.06 | -0.68 |
| novel_mir636 | XM_004936919.2 | FOXE3 | -1.02 | 1.47 | -0.68 |
| novel_mir1170 | XM_015276359.1 | NDST3 | 4.69 | -1.20 | -0.68 |
| novel_mir272 | XM_003640986.3 | ARID4B | -1.39 | 1.36 | -0.68 |
| novel_mir146 | XM_004942607.2 | FAM126B | -2.91 | 1.01 | -0.68 |
| novel_mir636 | NM_001031563.1 | CYR61 | -1.02 | 1.85 | -0.68 |
| novel_mir212 | NM_001270577.1 | UCKL1 | -1.60 | 1.22 | -0.68 |
| gga-miR-6654-3p | XM_015300578.1 | LOC426820 | -2.63 | 2.10 | -0.68 |
| novel_mir333 | XM_015292682.1 | HAS3 | -7.00 | 1.69 | -0.68 |
| novel_mir1256 | NM_001037274.1 | WNT7B | -5.48 | 2.21 | -0.68 |
| novel_mir326 | XM_004939652.2 | HIVEP1 | -1.08 | 1.33 | -0.68 |
| novel_mir636 | NM_001031044.1 | DUSP10 | -1.02 | 1.85 | -0.68 |
| novel_mir261 | XM_423102.4 | PPP1R3C | 2.94 | -1.23 | -0.68 |
| novel_mir1117 | XM_015276224.1 | TET2 | -1.82 | 1.89 | -0.68 |
| novel_mir636 | NM_001170344.1 | OSR2 | -1.02 | 1.05 | -0.68 |
| novel_mir272 | XM_003641901.3 | SLC9A5 | -1.39 | 1.03 | -0.68 |
| novel_mir636 | XM_003640686.3 | ITGB8 | -1.02 | 1.07 | -0.68 |
| novel_mir142 | NM_204891.1 | ATP2A3 | -2.10 | 1.93 | -0.68 |
| novel_mir272 | XM_015279493.1 | FAM102A | -1.39 | 1.43 | -0.68 |
| novel_mir667 | XM_015300578.1 | LOC426820 | -1.86 | 2.10 | -0.68 |
| gga-miR-7475-5p | XM_420586.5 | PPP1R3B | 4.05 | -1.44 | -0.68 |
| novel_mir142 | XM_015295882.1 | NUFIP2 | -2.10 | 1.01 | -0.68 |
| novel_mir627 | XM_004936517.2 | FRMD6 | -1.72 | 1.56 | -0.67 |
| novel_mir735 | XM_015290490.1 | IER5 | -1.26 | 1.26 | -0.67 |
| gga-miR-128-2-5p | XM_015297000.1 | ERRFI1 | -1.10 | 3.11 | -0.67 |
| gga-miR-7475-5p | XM_004939681.2 | LOC101749447 | 4.05 | -1.16 | -0.67 |
| novel_mir272 | XM_419779.5 | FRK | -1.39 | 1.09 | -0.67 |
| novel_mir636 | XM_001234009.4 | ZSWIM6 | -1.02 | 2.22 | -0.67 |
| novel_mir272 | NM_204432.1 | ARC | -1.39 | 3.70 | -0.67 |
| novel_mir304 | NM_205253.2 | CEBPB | -1.68 | 1.49 | -0.67 |
| novel_mir198 | NM_204535.4 | SPP1 | -5.65 | 3.44 | -0.67 |
| novel_mir261 | XM_004939658.2 | MAK | 2.94 | -1.83 | -0.67 |
| gga-miR-6570-5p | XM_001232082.4 | FAM102B | -2.83 | 2.20 | -0.67 |
| novel_mir272 | XM_003641088.3 | MAMLD1 | -1.39 | 2.17 | -0.67 |
| novel_mir636 | XM_003642734.3 | PHLDA3 | -1.02 | 1.84 | -0.67 |
| novel_mir458 | NM_001171771.1 | APC2 | -1.34 | 1.49 | -0.67 |
| novel_mir272 | XM_004945037.2 | SPRY4 | -1.39 | 1.69 | -0.67 |
| gga-miR-128-2-5p | XM_004947494.2 | KAZN | -1.10 | 1.54 | -0.67 |
| novel_mir326 | XM_015278663.1 | LOC100858332 | -1.08 | 1.58 | -0.67 |
| novel_mir704 | XM_004938920.2 | CREBZF | 2.35 | -1.07 | -0.67 |
| novel_mir272 | XM_015282496.1 | ANKRD12 | -1.39 | 1.11 | -0.67 |
| novel_mir853 | XM_015292422.1 | MYLK3 | -5.48 | 1.01 | -0.67 |
| novel_mir198 | NM_205206.1 | INHBB | -5.65 | 1.22 | -0.67 |
| novel_mir655 | NM_001199504.1 | PHYHIPL | -5.42 | 1.04 | -0.67 |
| novel_mir272 | XM_015291070.1 | PTCH2 | -1.39 | 1.56 | -0.67 |
| gga-miR-6654-3p | NM_205200.1 | FST | -2.63 | 2.83 | -0.67 |
| novel_mir326 | NM_205237.3 | BMP4 | -1.08 | 1.66 | -0.67 |
| novel_mir1037 | XM_004944605.2 | KLF15 | 1.69 | -1.17 | -0.66 |
| gga-miR-128-2-5p | NM_204484.2 | SOX4 | -1.10 | 1.09 | -0.66 |
| novel_mir326 | XM_015292551.1 | IRF8 | -1.08 | 2.03 | -0.66 |
| gga-miR-6654-3p | XM_015275647.1 | ULK1 | -2.63 | 1.34 | -0.66 |
| novel_mir636 | XM_003641641.3 | OBSL1 | -1.02 | 1.69 | -0.66 |
| novel_mir1256 | XM_004938278.2 | CBLB | -5.48 | 1.13 | -0.66 |
| novel_mir512 | XM_015282444.1 | ADAMTS20 | -2.18 | 1.02 | -0.66 |
| gga-miR-128-2-5p | XM_015299654.1 | TNS4 | -1.10 | 1.32 | -0.66 |
| novel_mir272 | XM_015286678.1 | LOC101751827 | -1.39 | 1.57 | -0.66 |
| novel_mir264 | XM_004938821.2 | ZC3H12C | -6.02 | 1.71 | -0.66 |
| novel_mir636 | XM_015277882.1 | LOC101749060 | -1.02 | 1.55 | -0.66 |
| novel_mir198 | XM_015283529.1 | JAG1 | -5.65 | 1.59 | -0.66 |
| novel_mir400 | XM_003640824.3 | SULF1 | -4.70 | 1.91 | -0.66 |
| novel_mir326 | XM_015293536.1 | DND1 | -1.08 | 1.65 | -0.66 |
| novel_mir304 | XM_015282405.1 | NR4A3 | -1.68 | 3.17 | -0.66 |
| novel_mir1256 | XM_004938794.2 | FAM123A | -5.48 | 1.12 | -0.66 |
| novel_mir509 | XM_015283933.1 | LOC107052968 | -1.35 | 2.03 | -0.66 |
| novel_mir316 | XM_015281606.1 | SKIDA1 | 5.43 | -1.12 | -0.66 |
| gga-miR-6654-3p | XM_003642861.3 | SBNO2 | -2.63 | 1.21 | -0.66 |
| novel_mir142 | NM_204928.1 | MNX1 | -2.10 | 1.72 | -0.66 |
| novel_mir400 | XM_004947281.2 | HELZ2 | -4.70 | 1.04 | -0.66 |
| novel_mir304 | XM_015283145.1 | TRIB1 | -1.68 | 3.05 | -0.66 |
| novel_mir296 | XM_015299781.1 | EVA1C | -4.48 | 1.20 | -0.66 |
| novel_mir326 | XM_015290911.1 | PFKFB3 | -1.08 | 2.51 | -0.66 |
| novel_mir1117 | XM_015277935.1 | GATA6 | -1.82 | 1.15 | -0.66 |
| gga-miR-6654-3p | XM_015280026.1 | LOC101749610 | -2.63 | 1.16 | -0.66 |
| novel_mir226 | XM_004944586.2 | ABHD6 | -1.13 | 1.26 | -0.66 |
| novel_mir470 | XM_015299148.1 | SLC6A17 | -5.42 | 1.45 | -0.66 |
| novel_mir142 | XM_015299177.1 | ADAMTS1 | -2.10 | 3.64 | -0.66 |
| novel_mir272 | NM_204781.1 | EPHA4 | -1.39 | 2.17 | -0.66 |
| novel_mir272 | NM_001001472.2 | NFKBIA | -1.39 | 1.82 | -0.66 |
| novel_mir261 | NM_001199643.2 | SYT13 | 2.94 | -1.45 | -0.66 |
| novel_mir304 | NM_204642.1 | CBX4 | -1.68 | 2.05 | -0.66 |
| novel_mir1117 | XM_015272740.1 | LOC107049350 | -1.82 | 1.53 | -0.66 |
| novel_mir636 | XM_015300220.1 | LOC107055390 | -1.02 | 3.42 | -0.66 |
| novel_mir627 | XM_015283234.1 | DENND3 | -1.72 | 1.20 | -0.66 |
| novel_mir509 | XM_003641641.3 | OBSL1 | -1.35 | 1.69 | -0.66 |
| novel_mir344 | XM_015294014.1 | IRF1 | -1.28 | 2.95 | -0.66 |
| novel_mir326 | XM_015282428.1 | PRICKLE1 | -1.08 | 1.62 | -0.66 |
| novel_mir142 | XM_417765.5 | ZC3H12A | -2.10 | 1.76 | -0.66 |
| novel_mir636 | XM_004947281.2 | HELZ2 | -1.02 | 1.04 | -0.66 |
| novel_mir636 | XM_015295905.1 | FKBP6 | -1.02 | 1.28 | -0.66 |
| novel_mir131 | XM_004936518.2 | FRMD6 | -2.07 | 1.56 | -0.66 |
| novel_mir1117 | NM_204484.2 | SOX4 | -1.82 | 1.09 | -0.66 |
| novel_mir655 | XM_015299148.1 | SLC6A17 | -5.42 | 1.45 | -0.66 |
| novel_mir247 | XM_004947403.2 | TMEM240 | -1.71 | 1.45 | -0.66 |
| novel_mir636 | XM_418838.4 | EEPD1 | -1.02 | 1.06 | -0.66 |
| novel_mir764 | XM_015292682.1 | HAS3 | -5.98 | 1.69 | -0.66 |
| novel_mir226 | NM_204618.2 | NFIL3 | -1.13 | 1.82 | -0.66 |
| novel_mir898 | XM_015284484.1 | CEP85L | -6.45 | 1.30 | -0.66 |
| novel_mir509 | NM_205019.2 | K123 | -1.35 | 1.46 | -0.66 |
| novel_mir198 | XM_015276814.1 | EPHA4 | -5.65 | 2.17 | -0.66 |
| novel_mir198 | XM_015297790.1 | EVA1B | -5.65 | 1.40 | -0.66 |
| novel_mir509 | XM_004938821.2 | ZC3H12C | -1.35 | 1.71 | -0.65 |
| novel_mir1256 | NM_204118.2 | AHR | -5.48 | 1.04 | -0.65 |
| novel_mir209 | XM_003642720.3 | KCNC4 | -1.23 | 1.85 | -0.65 |
| novel_mir141 | XM_001233583.4 | KLF4 | -1.27 | 1.18 | -0.65 |
| novel_mir624 | XM_015300114.1 | CILP2 | -5.70 | 1.80 | -0.65 |
| gga-miR-6654-3p | NM_001199273.1 | ETV6 | -2.63 | 1.25 | -0.65 |
| novel_mir142 | XM_015300578.1 | LOC426820 | -2.10 | 2.10 | -0.65 |
| novel_mir1256 | NM_001037275.2 | TRIM71 | -5.48 | 1.72 | -0.65 |
| novel_mir542 | NM_204681.2 | MGAT4C | -4.98 | 1.02 | -0.65 |
| novel_mir326 | XM_004947403.2 | TMEM240 | -1.08 | 1.45 | -0.65 |
| novel_mir304 | NM_001031537.1 | AREG | -1.68 | 1.76 | -0.65 |
| novel_mir683 | XM_015280876.1 | AQP11 | 7.90 | -1.00 | -0.65 |
| novel_mir627 | XM_015277692.1 | IL1R1 | -1.72 | 1.81 | -0.65 |
| novel_mir333 | XM_015293536.1 | DND1 | -7.00 | 1.65 | -0.65 |
| novel_mir636 | XM_015293935.1 | MAML1 | -1.02 | 1.05 | -0.65 |
| novel_mir131 | XM_003642861.3 | SBNO2 | -2.07 | 1.21 | -0.65 |
| novel_mir512 | XM_004939028.2 | ASB10 | -2.18 | 2.19 | -0.65 |
| novel_mir198 | XM_422158.5 | RND3 | -5.65 | 2.06 | -0.65 |
| novel_mir137 | XM_003641641.3 | OBSL1 | -1.76 | 1.69 | -0.65 |
| gga-miR-6570-5p | XM_015272740.1 | LOC107049350 | -2.83 | 1.53 | -0.65 |
| novel_mir1240 | NM_204136.2 | EGR1 | -5.35 | 2.26 | -0.65 |
| novel_mir400 | XM_004935937.2 | EVA1A | -4.70 | 1.28 | -0.65 |
| novel_mir142 | XM_015272640.1 | CLCF1 | -2.10 | 1.89 | -0.65 |
| novel_mir458 | XM_015294500.1 | SCNN1G | -1.34 | 1.04 | -0.65 |
| novel_mir909 | XM_004944605.2 | KLF15 | 3.28 | -1.17 | -0.65 |
| novel_mir627 | NM_204891.1 | ATP2A3 | -1.72 | 1.93 | -0.65 |
| novel_mir272 | NM_001030749.1 | ELK3 | -1.39 | 1.11 | -0.65 |
| novel_mir333 | XM_015297450.1 | ARID5A | -7.00 | 2.44 | -0.65 |
| novel_mir918 | XM_004941244.2 | NDST3 | 4.69 | -1.20 | -0.65 |
| novel_mir1117 | XM_015282942.1 | LOC107052763 | -1.82 | 1.01 | -0.65 |
| novel_mir801 | XM_015282444.1 | ADAMTS20 | -4.60 | 1.02 | -0.65 |
| gga-miR-6654-3p | XM_015299148.1 | SLC6A17 | -2.63 | 1.45 | -0.65 |
| novel_mir1256 | XM_015290010.1 | ETV6 | -5.48 | 1.25 | -0.65 |
| novel_mir1256 | XM_003640686.3 | ITGB8 | -5.48 | 1.07 | -0.65 |
| gga-miR-6654-3p | NM_001199549.1 | SLC38A4 | -2.63 | 1.05 | -0.65 |
| novel_mir142 | XM_015282955.1 | OSR2 | -2.10 | 1.05 | -0.65 |
| novel_mir272 | XM_015280026.1 | LOC101749610 | -1.39 | 1.16 | -0.65 |
| novel_mir898 | NM_001270577.1 | UCKL1 | -6.45 | 1.22 | -0.65 |
| gga-miR-12245-3p | NM_001030828.1 | SLC46A3 | 2.23 | -1.84 | -0.64 |
| novel_mir1256 | XM_015287459.1 | KDM7A | -5.48 | 1.03 | -0.64 |
| novel_mir142 | XM_015300114.1 | CILP2 | -2.10 | 1.80 | -0.64 |
| novel_mir304 | NM_001199909.1 | PDK4 | -1.68 | 1.19 | -0.64 |
| novel_mir296 | NM_204681.2 | MGAT4C | -4.48 | 1.02 | -0.64 |
| novel_mir333 | XM_015281780.1 | LOC107049140 | -7.00 | 1.26 | -0.64 |
| novel_mir146 | NM_001317736.1 | TMEM136-1 | -2.91 | 1.63 | -0.64 |
| novel_mir261 | NM_001030668.2 | HNF1A | 2.94 | -1.35 | -0.64 |
| novel_mir874 | NM_204540.1 | SOCS2 | -5.22 | 1.91 | -0.64 |
| novel_mir142 | XM_015273518.1 | LOC100857380 | -2.10 | 3.92 | -0.64 |
| novel_mir142 | XM_001233342.3 | LOC770026 | -2.10 | 1.41 | -0.64 |
| novel_mir627 | NM_001012760.1 | TSC22D1 | -1.72 | 1.32 | -0.64 |
| gga-miR-6570-5p | XM_003641088.3 | MAMLD1 | -2.83 | 2.17 | -0.64 |
| novel_mir146 | NM_001270577.1 | UCKL1 | -2.91 | 1.22 | -0.64 |
| novel_mir636 | XM_015276223.1 | TET2 | -1.02 | 1.89 | -0.64 |
| novel_mir326 | XM_015299299.1 | RUNX1 | -1.08 | 1.45 | -0.64 |
| novel_mir272 | XM_015299978.1 | MIDN | -1.39 | 1.30 | -0.64 |
| novel_mir356 | NM_001030893.2 | NR0B2 | -5.29 | 1.33 | -0.64 |
| novel_mir344 | XM_015287767.1 | SERPINA10 | -1.28 | 1.10 | -0.64 |
| novel_mir261 | XM_015299133.1 | MTCH1 | 2.94 | -1.02 | -0.64 |
| novel_mir488 | XM_004947281.2 | HELZ2 | -1.57 | 1.04 | -0.64 |
| novel_mir847 | XM_015284449.1 | MAFF | -1.75 | 1.20 | -0.64 |
| gga-miR-6654-3p | XM_004936517.2 | FRMD6 | -2.63 | 1.56 | -0.64 |
| novel_mir304 | XM_418054.5 | PRELP | -1.68 | 6.06 | -0.64 |
| novel_mir655 | XM_015280026.1 | LOC101749610 | -5.42 | 1.16 | -0.64 |
| novel_mir488 | NM_001278132.1 | CREM | -1.57 | 2.12 | -0.64 |
| gga-miR-6654-3p | XM_015296040.1 | SERPINF2 | -2.63 | 1.12 | -0.64 |
| gga-miR-6654-3p | XM_015290526.1 | TNN | -2.63 | 1.53 | -0.64 |
| novel_mir636 | NM_205237.3 | BMP4 | -1.02 | 1.66 | -0.64 |
| novel_mir636 | XM_015295882.1 | NUFIP2 | -1.02 | 1.01 | -0.64 |
| novel_mir272 | NM_205420.1 | GATA6 | -1.39 | 1.15 | -0.64 |
| novel_mir627 | XM_424739.5 | PLK2 | -1.72 | 1.27 | -0.64 |
| novel_mir272 | NM_001012693.1 | MGAT3 | -1.39 | 1.17 | -0.64 |
| novel_mir272 | XM_003643105.3 | FRMPD1 | -1.39 | 1.30 | -0.64 |
| novel_mir141 | XM_015286661.1 | HPS5 | -1.27 | 1.53 | -0.64 |
| novel_mir636 | XM_004938794.2 | FAM123A | -1.02 | 1.12 | -0.64 |
| novel_mir326 | XM_015282060.1 | LOC101750112 | -1.08 | 2.40 | -0.64 |
| novel_mir261 | NM_205002.1 | ID2 | 2.94 | -1.56 | -0.64 |
| gga-miR-6654-3p | XM_420888.3 | EGR4 | -2.63 | 4.89 | -0.64 |
| novel_mir427 | XM_015282955.1 | OSR2 | -5.80 | 1.05 | -0.64 |
| novel_mir1117 | XM_004940457.2 | BEND6 | -1.82 | 1.27 | -0.64 |
| novel_mir272 | NM_001171771.1 | APC2 | -1.39 | 1.49 | -0.64 |
| novel_mir1263 | XM_004947281.2 | HELZ2 | -5.94 | 1.04 | -0.63 |
| novel_mir932 | NM_001171771.1 | APC2 | -6.02 | 1.49 | -0.63 |
| novel_mir272 | XM_015278663.1 | LOC100858332 | -1.39 | 1.58 | -0.63 |
| novel_mir1018 | XM_417765.5 | ZC3H12A | -1.41 | 1.76 | -0.63 |
| novel_mir198 | NM_204316.3 | ADORA1 | -5.65 | 2.04 | -0.63 |
| novel_mir715 | XM_004945058.2 | JAKMIP2 | -5.22 | 1.12 | -0.63 |
| novel_mir247 | XM_015291070.1 | PTCH2 | -1.71 | 1.56 | -0.63 |
| novel_mir488 | XM_418054.5 | PRELP | -1.57 | 6.06 | -0.63 |
| novel_mir326 | XM_001232150.4 | EMX1 | -1.08 | 1.67 | -0.63 |
| novel_mir458 | XM_015296994.1 | PADI2 | -1.34 | 1.58 | -0.63 |
| novel_mir788 | XM_015299781.1 | EVA1C | -1.66 | 1.20 | -0.63 |
| novel_mir1143 | XM_004941288.2 | PAK6 | 2.07 | -1.05 | -0.63 |
| gga-miR-7475-5p | XM_015291321.1 | C8H1ORF210 | 4.05 | -1.08 | -0.63 |
| novel_mir142 | XM_015287724.1 | ISM2 | -2.10 | 1.79 | -0.63 |
| novel_mir247 | XM_015284702.1 | GABRR2 | -1.71 | 1.92 | -0.63 |
| novel_mir226 | NM_001012760.1 | TSC22D1 | -1.13 | 1.32 | -0.63 |
| novel_mir627 | NM_001199415.1 | PRRG4 | -1.72 | 2.78 | -0.63 |
| gga-miR-128-2-5p | XM_015300578.1 | LOC426820 | -1.10 | 2.10 | -0.63 |
| novel_mir209 | XM_004947403.2 | TMEM240 | -1.23 | 1.45 | -0.63 |
| gga-miR-6570-5p | XM_425541.5 | ADAMTS5 | -2.83 | 2.10 | -0.63 |
| novel_mir264 | XM_418054.5 | PRELP | -6.02 | 6.06 | -0.63 |
| novel_mir142 | XM_004944481.2 | LOC101751348 | -2.10 | 1.87 | -0.63 |
| novel_mir333 | XM_015294500.1 | SCNN1G | -7.00 | 1.04 | -0.63 |
| novel_mir264 | XM_015291573.1 | CYP2J6L3 | -6.02 | 1.66 | -0.63 |
| gga-miR-6654-3p | XM_015296101.1 | SSH2 | -2.63 | 1.15 | -0.63 |
| novel_mir512 | XM_001232150.4 | EMX1 | -2.18 | 1.67 | -0.63 |
| novel_mir1117 | XM_015273034.1 | TUT1 | -1.82 | 1.24 | -0.63 |
| novel_mir1107 | XM_004939681.2 | LOC101749447 | 8.77 | -1.16 | -0.63 |
| novel_mir488 | NM_205298.2 | ETS1 | -1.57 | 1.66 | -0.63 |
| novel_mir333 | XM_015295882.1 | NUFIP2 | -7.00 | 1.01 | -0.63 |
| novel_mir326 | XM_421754.5 | DUSP5 | -1.08 | 2.88 | -0.63 |
| novel_mir874 | XM_015281780.1 | LOC107049140 | -5.22 | 1.26 | -0.63 |
| novel_mir488 | XM_004944808.2 | ATP10B | -1.57 | 1.39 | -0.63 |
| novel_mir1128 | XM_015281606.1 | SKIDA1 | 8.06 | -1.12 | -0.63 |
| novel_mir636 | NM_001001530.1 | LEPREL1 | -1.02 | 1.01 | -0.63 |
| novel_mir226 | XM_015296101.1 | SSH2 | -1.13 | 1.15 | -0.63 |
| novel_mir642 | NM_001277544.1 | RGS16 | -6.32 | 3.83 | -0.63 |
| novel_mir226 | NM_001031012.1 | TUBB6 | -1.13 | 1.01 | -0.62 |
| novel_mir601 | XM_004937677.2 | GPRC5A | -5.48 | 1.81 | -0.62 |
| novel_mir142 | NM_001171771.1 | APC2 | -2.10 | 1.49 | -0.62 |
| novel_mir142 | XM_015299978.1 | MIDN | -2.10 | 1.30 | -0.62 |
| novel_mir326 | XM_015282063.1 | LOC420486 | -1.08 | 3.94 | -0.62 |
| novel_mir209 | XM_415813.5 | SEBOX | -1.23 | 1.28 | -0.62 |
| novel_mir272 | XM_015272984.1 | NHS | -1.39 | 1.70 | -0.62 |
| novel_mir212 | XM_015299148.1 | SLC6A17 | -1.60 | 1.45 | -0.62 |
| novel_mir458 | NM_204316.3 | ADORA1 | -1.34 | 2.04 | -0.62 |
| novel_mir272 | NM_204401.2 | TRIB2 | -1.39 | 2.35 | -0.62 |
| novel_mir326 | XM_015298873.1 | CSF1 | -1.08 | 1.32 | -0.62 |
| novel_mir601 | NM_204274.1 | CTGF | -5.48 | 1.39 | -0.62 |
| novel_mir264 | XM_003641038.3 | PRDM1 | -6.02 | 1.32 | -0.62 |
| novel_mir655 | XM_004949164.2 | TMEM2 | -5.42 | 1.30 | -0.62 |
| novel_mir264 | XM_418566.5 | FZD8 | -6.02 | 1.77 | -0.62 |
| novel_mir304 | XM_015282942.1 | LOC107052763 | -1.68 | 1.01 | -0.62 |
| novel_mir458 | NM_001031311.1 | PIK3CB | -1.34 | 1.53 | -0.62 |
| novel_mir264 | XM_015299654.1 | TNS4 | -6.02 | 1.32 | -0.62 |
| novel_mir509 | XM_015300585.1 | LOC426820 | -1.35 | 2.10 | -0.62 |
| novel_mir142 | XM_004945054.2 | JAKMIP2 | -2.10 | 1.12 | -0.62 |
| novel_mir636 | XM_015298704.1 | LOC101749375 | -1.02 | 1.04 | -0.62 |
| novel_mir120 | XM_004943607.2 | ALPK3 | 3.10 | -1.02 | -0.62 |
| novel_mir636 | XM_417497.5 | TFAP2C | -1.02 | 1.24 | -0.62 |
| novel_mir198 | XM_004935937.2 | EVA1A | -5.65 | 1.28 | -0.62 |
| novel_mir1143 | XM_015278478.1 | ZIC3 | 2.07 | -1.17 | -0.62 |
| novel_mir1117 | XM_003642903.3 | ONECUT2 | -1.82 | 1.27 | -0.62 |
| novel_mir458 | XM_015272984.1 | NHS | -1.34 | 1.70 | -0.62 |
| novel_mir146 | XM_015287834.1 | NUAK1 | -2.91 | 1.05 | -0.62 |
| novel_mir198 | XM_004942150.2 | HECTD2 | -5.65 | 1.06 | -0.62 |
| novel_mir333 | XM_015283837.1 | ZFP36L2 | -7.00 | 1.75 | -0.62 |
| gga-miR-128-2-5p | XM_001232150.4 | EMX1 | -1.10 | 1.67 | -0.62 |
| novel_mir1117 | XM_015272984.1 | NHS | -1.82 | 1.70 | -0.62 |
| novel_mir682 | XM_015277004.1 | IRS1 | -1.21 | 1.03 | -0.62 |
| novel_mir636 | XM_003640531.2 | ARHGEF7 | -1.02 | 1.08 | -0.62 |
| novel_mir261 | XM_426034.4 | NHLRC1 | 2.94 | -1.23 | -0.62 |
| novel_mir636 | XM_003642903.3 | ONECUT2 | -1.02 | 1.27 | -0.62 |
| novel_mir458 | XM_004938794.2 | FAM123A | -1.34 | 1.12 | -0.61 |
| gga-miR-6654-3p | XM_015279230.1 | MLKL | -2.63 | 1.78 | -0.61 |
| novel_mir229 | XM_015277593.1 | PDE4D | -5.48 | 1.29 | -0.61 |
| novel_mir188 | XM_015282955.1 | OSR2 | -5.48 | 1.05 | -0.61 |
| novel_mir226 | NM_001031072.1 | MAP7 | -1.13 | 1.06 | -0.61 |
| novel_mir146 | XM_001231361.4 | ASB10 | -2.91 | 2.19 | -0.61 |
| novel_mir430 | XM_015299823.1 | LOC107055332 | 6.31 | -1.66 | -0.61 |
| novel_mir272 | NM_001252324.1 | FNDC3B | -1.39 | 1.57 | -0.61 |
| gga-miR-7475-5p | XM_015299132.1 | MTCH1 | 4.05 | -1.02 | -0.61 |
| novel_mir261 | XM_004934986.2 | TRAF3IP3 | 2.94 | -2.44 | -0.61 |
| gga-miR-128-2-5p | XM_004934869.2 | KCNC4 | -1.10 | 1.85 | -0.61 |
| novel_mir212 | XM_015298695.1 | LOC101749375 | -1.60 | 1.04 | -0.61 |
| novel_mir198 | NM_001037275.2 | TRIM71 | -5.65 | 1.72 | -0.61 |
| novel_mir304 | XM_015277255.1 | HMGCS1 | -1.68 | 1.02 | -0.61 |
| novel_mir1256 | NM_001079726.1 | LOC420419 | -5.48 | 1.20 | -0.61 |
| gga-miR-12245-3p | XM_015299823.1 | LOC107055332 | 2.23 | -1.66 | -0.61 |
| novel_mir1124 | XM_015288906.1 | BAG3 | -4.80 | 1.45 | -0.61 |
| novel_mir198 | XM_421754.5 | DUSP5 | -5.65 | 2.88 | -0.61 |
| gga-miR-128-2-5p | XM_001234009.4 | ZSWIM6 | -1.10 | 2.22 | -0.61 |
| novel_mir272 | XM_015276127.1 | SPRY1 | -1.39 | 1.71 | -0.61 |
| novel_mir564 | NM_001079726.1 | LOC420419 | -4.80 | 1.20 | -0.61 |
| novel_mir304 | NM_001257283.1 | MCL1 | -1.68 | 1.17 | -0.61 |
| novel_mir1051 | XM_015296101.1 | SSH2 | -4.70 | 1.15 | -0.61 |
| novel_mir272 | XM_001234445.3 | IFITM10 | -1.39 | 1.78 | -0.61 |
| novel_mir624 | XM_416200.4 | GPRC5A | -5.70 | 1.81 | -0.61 |
| novel_mir636 | XM_015286158.1 | PRRG4 | -1.02 | 2.78 | -0.61 |
| novel_mir226 | XM_015283435.1 | BMP2 | -1.13 | 1.70 | -0.61 |
| novel_mir272 | XM_015285158.1 | EVA1A | -1.39 | 1.28 | -0.61 |
| novel_mir226 | XM_015293658.1 | HTR4 | -1.13 | 1.45 | -0.61 |
| novel_mir898 | NM_001040018.1 | OLFML3 | -6.45 | 1.58 | -0.61 |
| novel_mir226 | XM_015287552.1 | BDKRB2 | -1.13 | 1.72 | -0.61 |
| novel_mir1256 | NM_001030969.1 | KLF6 | -5.48 | 4.00 | -0.61 |
| novel_mir655 | XM_422087.5 | TFCP2L1 | -5.42 | 1.03 | -0.61 |
| novel_mir624 | XM_015300024.1 | MEX3D | -5.70 | 1.03 | -0.61 |
| novel_mir1107 | XM_004937095.2 | LOC100857744 | 8.77 | -2.17 | -0.61 |
| novel_mir272 | XM_015286034.1 | SMOX | -1.39 | 1.72 | -0.61 |
| novel_mir198 | XM_015275936.1 | NFATC1 | -5.65 | 1.01 | -0.61 |
| novel_mir788 | XM_015299654.1 | TNS4 | -1.66 | 1.32 | -0.61 |
| novel_mir458 | XM_003642903.3 | ONECUT2 | -1.34 | 1.27 | -0.61 |
| novel_mir202 | XM_015297790.1 | EVA1B | -4.48 | 1.40 | -0.61 |
| novel_mir518 | XM_015297790.1 | EVA1B | -4.48 | 1.40 | -0.61 |
| novel_mir627 | XM_015275647.1 | ULK1 | -1.72 | 1.34 | -0.61 |
| novel_mir304 | XM_015283837.1 | ZFP36L2 | -1.68 | 1.75 | -0.61 |
| novel_mir141 | XM_004947050.2 | SULF2 | -1.27 | 1.01 | -0.61 |
| novel_mir272 | XM_003642861.3 | SBNO2 | -1.39 | 1.21 | -0.61 |
| novel_mir198 | XM_422425.5 | PLK3 | -5.65 | 1.63 | -0.61 |
| novel_mir1037 | NM_204098.2 | FZD10 | 1.69 | -1.66 | -0.61 |
| novel_mir1128 | NM_001277837.1 | KCTD14 | 8.06 | -1.18 | -0.61 |
| novel_mir427 | XM_003641038.3 | PRDM1 | -5.80 | 1.32 | -0.61 |
| gga-miR-6654-3p | XM_001231361.4 | ASB10 | -2.63 | 2.19 | -0.61 |
| novel_mir898 | XM_015284186.1 | TNFAIP3 | -6.45 | 2.02 | -0.61 |
| novel_mir458 | XM_004934565.2 | RIPK4 | -1.34 | 2.07 | -0.60 |
| novel_mir146 | XM_015285802.1 | FBXL5 | -2.91 | 1.09 | -0.60 |
| gga-miR-6654-3p | NM_001012825.1 | RASA3 | -2.63 | 2.40 | -0.60 |
| novel_mir1117 | XM_003640342.3 | PIM3 | -1.82 | 1.07 | -0.60 |
| novel_mir655 | NM_001001530.1 | LEPREL1 | -5.42 | 1.01 | -0.60 |
| novel_mir636 | NM_001277606.1 | RRAD | -1.02 | 1.62 | -0.60 |
| novel_mir624 | NM_205253.2 | CEBPB | -5.70 | 1.49 | -0.60 |
| gga-miR-12245-3p | XM_015280876.1 | AQP11 | 2.23 | -1.00 | -0.60 |
| novel_mir427 | XM_015291448.1 | MUC4 | -5.80 | 1.50 | -0.60 |
| novel_mir488 | NM_204781.1 | EPHA4 | -1.57 | 2.17 | -0.60 |
| novel_mir1124 | NM_001031356.1 | PLEKHO1 | -4.80 | 1.93 | -0.60 |
| novel_mir636 | XM_003640342.3 | PIM3 | -1.02 | 1.07 | -0.60 |
| gga-miR-6654-3p | NM_001252254.1 | SLC26A6 | -2.63 | 2.50 | -0.60 |
| novel_mir247 | XM_015297968.1 | ETS1 | -1.71 | 1.66 | -0.60 |
| novel_mir326 | XM_001232892.3 | DUSP8 | -1.08 | 1.23 | -0.60 |
| novel_mir796 | NM_204274.1 | CTGF | -5.42 | 1.39 | -0.60 |
| novel_mir544 | XM_015294233.1 | SOCS1 | -6.14 | 1.95 | -0.60 |
| novel_mir583 | XM_015299959.1 | LOC107055350 | -4.70 | 1.48 | -0.60 |
| novel_mir140 | XM_004937095.2 | LOC100857744 | 5.69 | -2.17 | -0.60 |
| novel_mir796 | XM_004940646.2 | LOC101748744 | -5.42 | 1.84 | -0.60 |
| novel_mir142 | NM_001257283.1 | MCL1 | -2.10 | 1.17 | -0.60 |
| novel_mir636 | XM_004940646.2 | LOC101748744 | -1.02 | 1.84 | -0.60 |
| novel_mir470 | XM_015300114.1 | CILP2 | -5.42 | 1.80 | -0.60 |
| gga-miR-6654-3p | XM_004935096.2 | NFATC1 | -2.63 | 1.01 | -0.60 |
| novel_mir304 | XM_418566.5 | FZD8 | -1.68 | 1.77 | -0.60 |
| novel_mir458 | NM_001270577.1 | UCKL1 | -1.34 | 1.22 | -0.60 |
| novel_mir326 | XM_004939382.2 | CREB5 | -1.08 | 1.68 | -0.60 |
